# An orally active dual CBP/p300 degrader targets core dependencies of multiple myeloma

**DOI:** 10.1016/j.celrep.2026.117464

**Published:** 2026-06-02

**Authors:** Praveen Kumar Tiwari, Bomin Ku, Drew A. Harrison, Sarah Rizvi, Samuel Ojeda, Jessica Duffy, Leonie Cluse, Jennifer R. Devlin, Nenad Bartonicek, Olga Motorna, Ann-Sophie Koglin, Barbara Karakyriakou, Kaitlyn Gagnon, Ramya S. Iyer, Regina Egan, Pat Greninger, Lauren Benz, Caroline Greco, Julia Norton, Amruth Kumar, Eric F. Zaniewski, Soroush Hajizadeh, Robert Morris, Genna Mullen, Ajinkya S. Kawale, Sangwon Min, Sai Reddy Doda, Raghu Vannam, Lee Zou, Wilhelm Haas, Abner Louissaint, Ricky W. Johnstone, Christopher J. Ott

**Affiliations:** 1 Krantz Family Center for Cancer Research, Massachusetts General Hospital, Charlestown, MA 02129, USA; 2 Department of Medicine, Harvard Medical School, Boston, MA 02115, USA; 3 Peter MacCallum Cancer Center, Laboratory Research Division, Parkville, VIC 3052, Australia; 4 The Sir Peter MacCallum Department of Oncology, University of Melbourne, Parkville, VIC 3052, Australia; 5 These authors contributed equally; 6 Lead contact

## Abstract

The enhancer lysine acetyltransferases CBP/p300 are compelling targets for multiple myeloma therapy. Chemical inhibition of these multidomain factors, either through the bromodomain or the catalytic acetyltransferase domain, show promising activity in pre-clinical models. Chemical degradation is the only modality that can completely disrupt all functional domains. Our previous attempts to induce CBP/p300 targeted degradation led to a potent tool compound, dCBP-1. Here we comprehensively demonstrate across a large panel of cell lines how CBP/p300 degradation compares to inhibition, with pronounced selective antiproliferative activity toward multiple myeloma. We use chemical linker optimization strategies to create a compound with better pharmacokinetic properties. Through these we define an advanced analog of dCBP-1, dCBP-30, that has improved potency and improved *in vivo* properties including oral bioavailability. dCBP-30 led to potent and sustained loss of CBP and p300, potent inhibition of several myeloma-specific dependency programs, and elicits tumor reduction in xenograft models.

## In brief

Tiwari, Ku et al. introduce dCBP-30, a new CBP/p300 heterobifunctional degrader featuring a near-linkerless design. This degrader is highly potent in models of multiple myeloma, with significant activity and efficacy when dosed orally in tumor-bearing mice. This work advances CBP/p300 degradation as a promising therapeutic strategy for multiple myeloma.

## Graphical Abstract

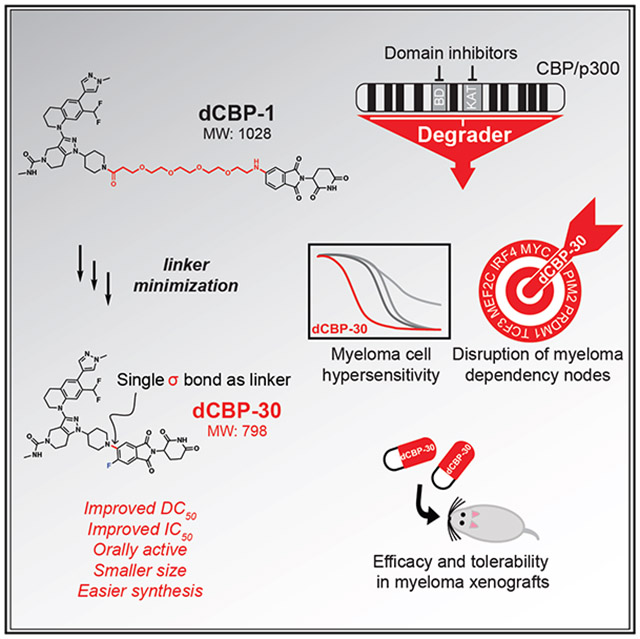

## INTRODUCTION

Inhibition of the enhancer acetyltransferases CBP and p300 is being intently pursued as a therapeutic strategy for cancer treatment. These highly homologous, multidomain regulators of transcriptional enhancer activity catalytically deposit acetylation marks on hundreds of substrate proteins.^1^ Chemical inhibitors of the lysine acetyltransferase (KAT) domain have been described, and they demonstrate promising preclinical activity in several distinct tumor types, including hormone receptor-driven cancers (breast and prostate) and multiple myeloma (MM).^2–6^ CBP/p300 also engage enhancers through a vast repertoire of protein-protein interactions facilitated by an array of functional domains that includes an acetyllysine reading bromodomain. Chemical inhibition of the CBP/p300 bromodomains can be achieved with a variety of structurally distinct chemo-types, several of which have advanced to human clinical investigation in solid and hematologic cancers, including MM.^7–9^ MM cells are particularly reliant on CBP/p300 activity for growth and survival, which has been demonstrated both with functional gene knockout studies and with targeted inhibitors, at least in part through direct downstream effects on the MYC/IRF4 transcriptional network.^5,10,11^ While promising, these inhibitors have limiting pharmacologic effects relatively restricted to their targeted domains. The large, multidomain nature of these factors should be most likely ablated using a chemical degradation strategy. Using a heterobifunctional design approach, we described dCBP-1, a proteolysis targeting chimera (PROTAC)-based chemical degrader of CBP/p300.^12^ With rapid and complete loss of both CBP and p300 in cells, we observed substantially increased cell killing effects *in vitro* when compared with established KAT and bromodomain inhibitors. Iterating on this heterobifunctional design strategy, we and others have described CBP/p300-targeting PROTACs with varying ligand and linker designs, establishing a class of pharmacologic tools for prosecuting CBP/p300 biological functions and with therapeutic potential.^13–24^

With this variety of chemical modulation emerging for CBP/p300-targeting cancer therapeutics, it is necessary to resolve their comparative effects and determine the contexts in which each modality may be maximally useful. Here we describe a comprehensive analysis of anticancer activity of CBP/p300 inhibitors and degraders, which demonstrate selective effects on a wide variety of cancer cell line models and generalized hypersensitivity of B lymphoid-derived tumor cells to CBP/p300 agents, with CBP/p300 degradation eliciting the most potent effects. We also substantially iterate on our original design of dCBP-1 to produce a more bioavailable and potent PROTAC that features a strikingly minimal linker design. Using this improved degrader, we demonstrate anti-myeloma activity via direct targeting of multiple established dependencies. Importantly, CBP/p300 degradation *in vivo* is tolerated at doses that elicit regression of MM xenografted tumors with oral administration, both alone and in combination with anti-myeloma standard of care therapy.

## RESULTS

### CBP/p300 degradation leads to distinct patterns of cancer cell killing

**Figure 1.**
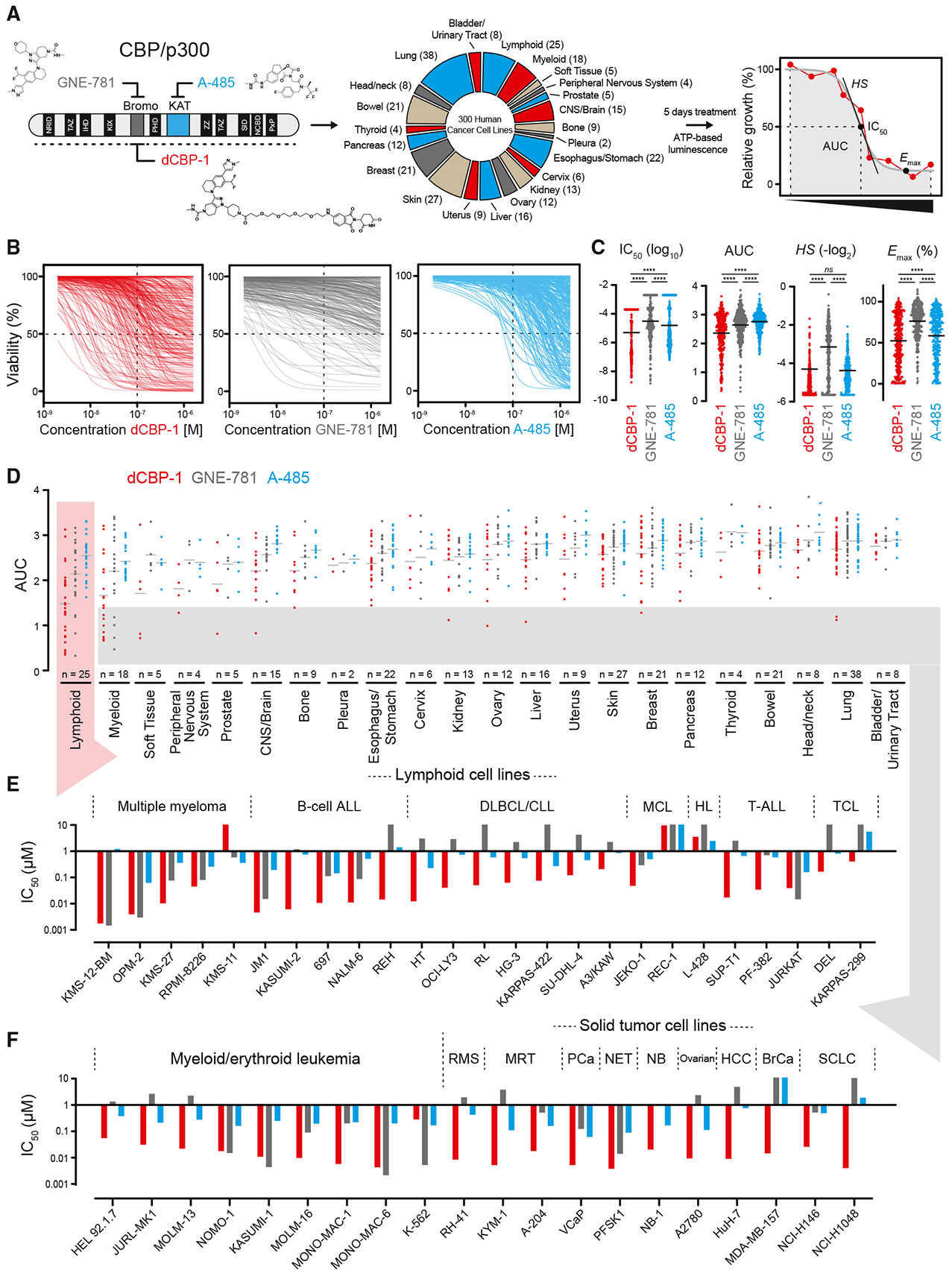
CBP/p300 degradation induces distinct pharmacologic cell killing effects compared with bromodomain and KAT domain inhibitors in cancer cell lines (A) Schematic of CBP/p300 domains and structures of relevant inhibitors GNE-781 (bromodomain), A-485 (catalytic KAT domain), and degrader dCBP-1 (left); cancer cell line panel used for comparative viability profiling (middle); and quantitative dose-response metrics derived from 5-day viability assay (right). (B) Compiled and overlaid dose-response curves across 300 cell lines for dCBP-1 (left), GNE-781 (middle), and A-485 (right). Each cell line was screened in duplicate; for clarity, a single replicate for each cell line is shown. (C) Quantification of dose-response IC_50_, area under the curve (AUC), Hill slope (*HS*), and maximum effect (*E*_max_); each dot represents the mean value for each cell line in the screen (*n* = 2 replicates per cell line); black line within each plot represents mean value across 300 cell lines. For IC_50_, if 50% inhibition was not determined within dose range tested, value was assigned at 100×the maximum dose tested; ****p < 0.0001, two-tailed paired *t* test; *ns*, not significant. (D) Average AUC values for each cell line categorized by lineage; gray bar within each plot represents mean value for each drug within lineage. (E) IC_50_ values for each lymphoid cell line in screen. MM, multiple myeloma; B-ALL, B cell acute lymphoblastic leukemia; DLBCL/CLL, diffuse large B cell lymphoma/chronic lymphocytic leukemia; MCL, mantle cell lymphoma; HL, Hodgkin’s lymphoma; T-ALL, T cell acute lymphoblastic leukemia; and TCL, T cell lymphoma; values represent mean of two replicate experiments. (F) IC_50_ values for most sensitive non-lymphoid cell lines in screen. RMS, rhabdomyosarcoma; MRT, malignant rhabdoid tumor; PCa, prostate cancer; NET, neuroendocrine tumor; NB, neuroblastoma; HCC, hepatocellular carcinoma; BrCa, breast cancer; and SCLC, small cell lung cancer; values represent mean of two replicate experiments. See also Table S1.

To determine the comparative pharmacologic effects of single-domain inhibition and complete degradation with a bifunctional PROTAC, we conducted a viability screen across 300 cancer cell lines treated with the bromodomain inhibitor GNE-781, the KAT domain inhibitor A-485, or dCBP-1 (Figure 1A). Cell lines were chosen from a broader set of lines as part of the Genomics of Drug Sensitivity in Cancer Consortium Project to enable rapid and robust viability profiling using ATP-based luminescence readouts after five days of treatment, with quantification of multi-parameter dose-response metrics (AUC, IC_50_, Hill slope [*HS*], and *E*_max_) that give a comprehensive picture of anti-cancer drug response *in vitro*.^25–27^ Broadly, dCBP-1 was more active than either bromodomain or KAT domain inhibition, with significantly lower AUC, IC_50_, and *E*_max_ across the cell line panel (Figures 1B and 1C; Table S1). Interestingly, the dose-response curve slopes of dCBP-1 and A-485 are not significantly different, while curve slope of the bromodomain inhibitor GNE-781 is significantly flatter for most cell lines tested. This is an indication that most cancer cell line dependency on CBP/p300 is a result of their KAT function, which may be blocked most readily through degradation, and that bromodomain inhibition in most cell contexts results in only partial attenuation of CBP/p300 activity, perhaps through allosteric effects on KAT activity or other mechanisms.^11^ By categorizing the cell lines by lineage origin, we observe cells derived from hematologic tumors with heightened sensitivity compared with non-hematologic lines, with the highest sensitivity in lymphoid-derived lines (Figure 1D). For every lineage, dCBP-1 was broadly more active than GNE-781 or A-485, demonstrating that increased potency is a general hallmark of chemical-induced protein degradation compared with discrete domain inhibition. Parsing the lymphoid-derived cell types in the screen into discrete tumor types, we find that MM-derived lines are broadly most sensitive to all three compounds (Figure 1E). Non-lymphoid-derived cell lines were also sensitive across each compound class, including myeloid-derived cells and a limited number of solid-tumor-derived models (Figure 1F). This included acute myeloid leukemia, prostate cancer, and neuroblastoma models, which have been explored by others as promising indications for anti-CBP/p300 agents including both inhibitors and degraders.^2,13,20,28,29^ Together, the dataset demonstrates that chemical-induced degradation of CBP/p300 more potently targets CBP/p300 activity across cancer cell contexts, tumor cell dependency on CBP/p300 expression across various tumor cells is most efficiently targeted with a degrader, and MM cell lines (more so than other tumor types) are at the leading edge of sensitivity to this agent class.

### Optimization of dCBP-1 leads to a potent bifunctional degrader with minimized linker

**Figure 2.**
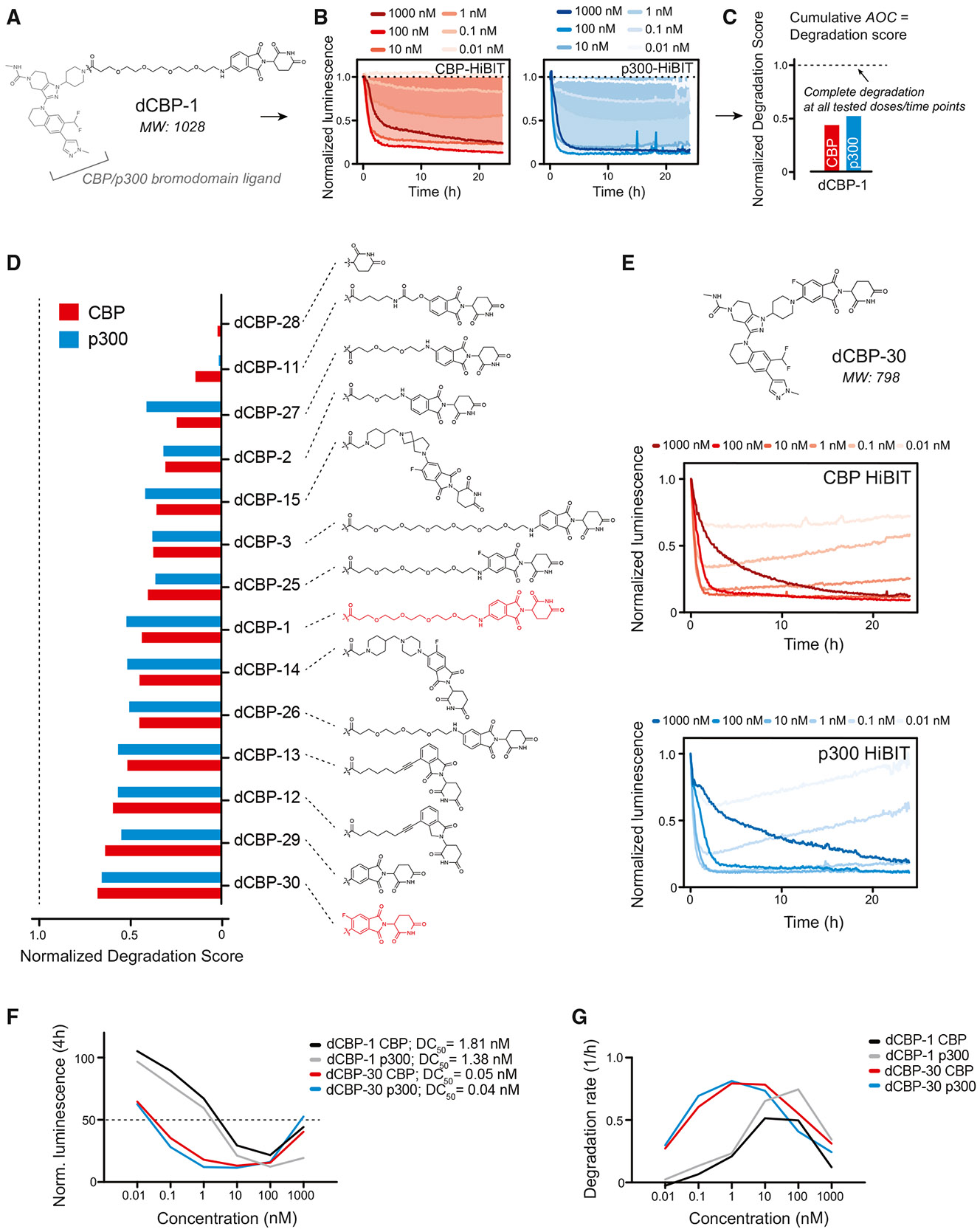
Development of an optimized, linker-minimized CBP/p300 degrader (A) Structure of dCBP-1. (B) Dynamic quantification of CBP and p300 degradation kinetics by dCBP-1 using area over the degradation curve (AOC) from HiBiT assay readout in HAP1 cells. (C) Derivation of a normalized CBP and p300 degradation score for dCBP-1 using cumulative AOC across the assayed dose range (0.01–1,000 nM). (D) Normalized degradation scores for CBP and p300 for dCBP-1 (red) and 13 analogs with varying linker and CRBN ligand structures. dCBP-30 displays superior dual degradation with minimal linker structure (also in red). (E) Structure of dCBP-30 (top); HAP1 HiBiT degradation curves for CBP and p300. Luminescence values are normalized to baseline time point and vehicle control (DMSO) treated cells. (F) Dose-response degradation of CBP and p300 after 4 h treatment comparing dCBP-1 with dCBP-30. (G) Dose-response degradation rate of CBP and p300 comparing dCBP-1 with dCBP-30. See also Figures S1 and S2 and Table S2.

Despite its potency in cell culture experiments, dCBP-1 was not designed as an optimized tool for *in vivo* studies. PROTAC optimization generally requires iterative refinement of chemical linker structures that can have significant effects on degradation activity, dynamics, cellular permeability, and *in vivo* pharmacokinetic properties. As medicinal chemistry approaches to PROTAC construction have matured over the last several years, there now exist numerous examples of refined structures that promote enhanced activity. In constructing a first generation CBP/p300 degrader, dCBP-1, we chose as a CBP/p300 targeted ligand GNE-781, a highly refined, potent, and selective bromodomain inhibitor.^30^ dCBP-1 was constructed by replacing the solvent-exposed tetrahydropyran ring of GNE-781 with a conjugatable piperidine, an exit vector predicted by *in silico* modeling and subsequently confirmed to be favorable for cereblon (CRBN) ternary complex formation (Figure 2A).^12,31^ dCBP-1 features a flexible PEG-4 linker attached to the C5 carbon of thalidomide, which affords effective and rapid degradation of both CBP and p300 in a variety of cell types. Using the same attachment site of the GNE-781 scaffold, we constructed a library of dCBP analogs featuring a variety of linker types and CRBN ligands. To assess degradation activity, we used a dynamic live-cell assay with endogenous HiBiT-tagged CBP and p300 in the haploid HAP1 cell line stably expressing LgBiT.^31^ From dose-responsive, dynamic degradation data we derived a normalized degradation score from the cumulative degrader activity between a dose range of 0.01–1,000 nM over 24 h (Figures 2B and 2C). The assays also allowed for derivation of traditional PROTAC metrics including maximal degradation percentage (D_max_), 50% maximal degradation concentration (DC_50_), and degradation rate (DR).

We constructed our library to include linker and CRBN-binding moieties that have been described by others as having the potential to confer increased potency, selectivity, and bioavailability in *in vivo* experiments. This included features such as spirocyclic and piperizine/piperidine heterocyclic linkers reported in clinical-grade PROTACs (dCBP-14 and dCBP-15) and rigidified alkyl/alkynl linkers (dCBP-12 and dCBP-13).^32–36^ We also synthesized thalidomide-based PROTACs with varying flexible PEG and alkyl chain lengths (dCBP-1, dCBP-2, dCBP-3, dCBP-11, dCBP-25, dCBP-26, and dCBP-27), some of which we have described previously.^31^ We also included compounds in which thalidomide or a glutarimide ring were conjugated directly to the piperidine of GNE-781 without any structured linker (dCBP-28, dCBP-29, and dCBP-30). We observed a wide range of CBP and p300 degradation activity across the library; to our surprise, thalidomide conjugation directly to GNE-781 (dCBP-29 and dCBP-30) showed the most activity (Figure 2D; Table S2). dCBP-30 features fluorine substitution adjacent to thalidomide linkage, a feature that increases lipophilicity, has been shown to decrease off-target degradation, and is slightly more active than the non-fluorinated dCBP-29.^37^ dCBP-30 has a molecular weight of 798 Daltons, a >22% reduction in size compared with dCBP-1 and lower than several other recently reported CBP/p300 PROTACs with *in vivo* activity (Figure 2E).^14,18,19^ dCBP-30 treatment displays rapid CBP/p300 degradation kinetics with DC_50_ at 4 h of 0.05 nM (CBP) and 0.04 nM (p300), and it shows a maximum DR (after 1 h treatment) of 0.79 with 1 nM (CBP) and 0.81 with 1 nM (p300) compared with 0.51 with 10 nM (CBP) and 0.74 with 100 nM (p300) for dCBP-1 (Figures 2F and 2G). We did observe a “hook effect” with dCBP-30 treatment at concentrations lower than that of dCBP-1; however, this was most pronounced only with micromolar dosing. Similar degradation dynamics in HAP1 cells were observed using capillary-based immunoassays, and degradation rescue was observed when cells were pre-treated with proteasome or neddylation inhibitors, or with excess free GNE-781 or pomalidomide, or in cells that lacked CRBN expression—confirming on-target, mechanism-based effects of dCBP-30 (Figure S1). To understand the mechanism by which dCBP-30 leads to increased degradation compared to dCBP-1, we quantified both membrane permeability and CRBN-bromodomain ternary complex formation in cell-free systems (PAMPA and AlphaScreen assays). dCBP-30 displayed a slightly attenuated ability to form a ternary complex between full-length CRBN compared with dCBP-1, yet had significantly more membrane permeability capability (Figure S2). Thus, while dramatically shortening the linker length to achieve a compound with more drug-like properties may slightly decrease the effects of dCBP-30 as a heterobifunctional inducer of protein-ligase proximity, this can be compensated for by increased membrane permeability and presumed increase in intracellular concentration, leading to more potent overall degradation activity.

### dCBP-30 has potent and selective effects on CBP and p300 in MM cells

**Figure 3.**
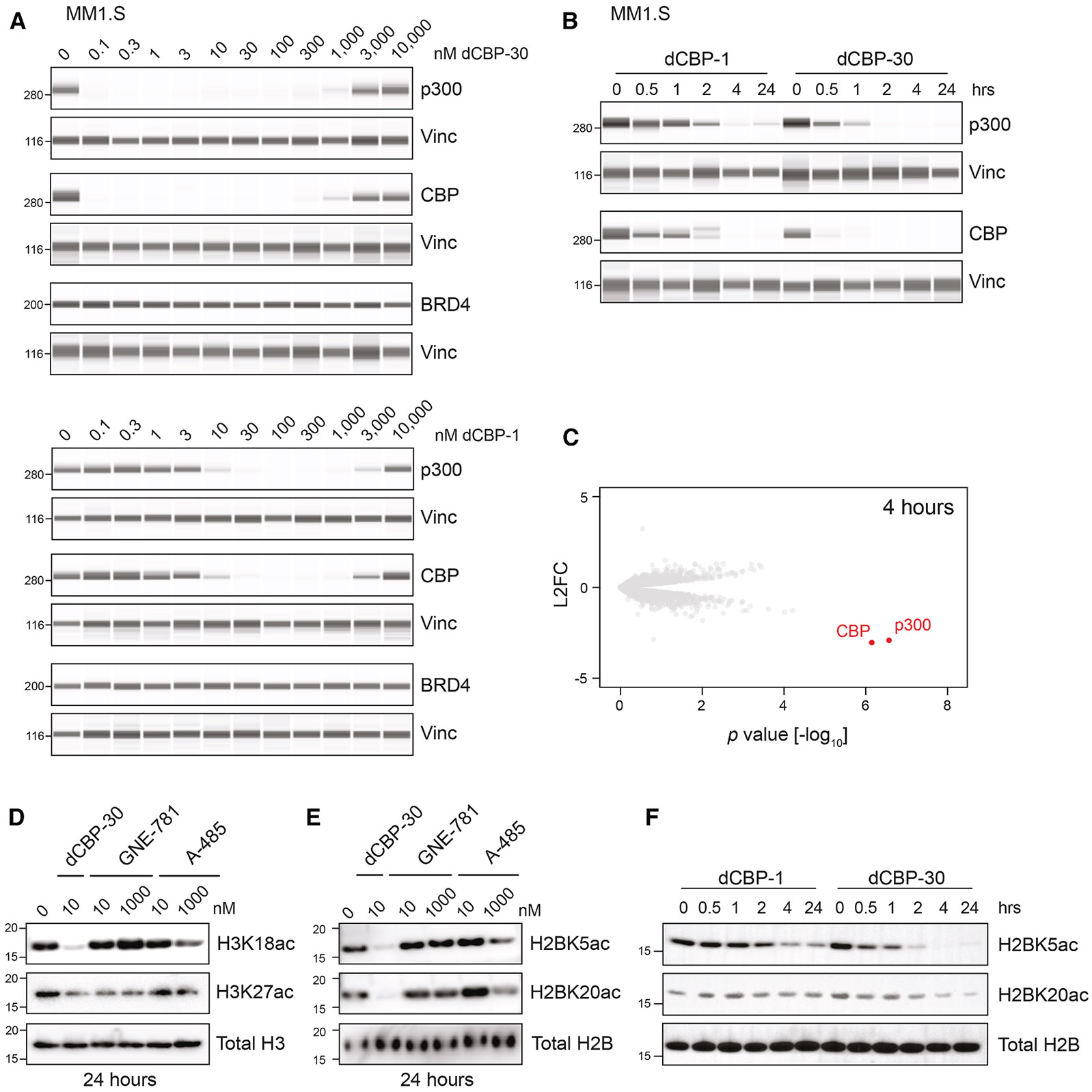
dCBP-30 induces potent and selective cellular degradation of CBP and p300 in multiple myeloma cells (A) Immunoassay measurements of CBP, p300, and BRD4 levels with dCBP-30 (top) or dCBP-1 (bottom) dose-response treatments with 4 h drug exposure in MM1.S cells; vinculin levels shown as loading controls. (B) Time-course immunoassay measurements of CBP and p300 levels with dCBP-30 or dCBP-1 treatment over 24 h in MM1.S cells, 10 nM. (C) Global proteomic assessment of dCBP-30 treatment (10 nM, 4 h) in MM1.S cells. (D and E) Immunoblotting results in MM1.S cells showing effects of dCBP-30, GNE-781, and A-485 on (D) histone H3 (H3K18ac and H3K27ac) and (E) histone H2B acetylation levels (H2BK5ac, H2BK20ac). (F) MM1.S cells were treated with 10 nM of either dCBP-1 or dCBP-30 over 24 h, followed by blotting for H2BK5ac and H2BK20ac. Total histone H3 or H2B levels are shown as control. See also Figure S3 and Table S3.

We next sought to characterize dCBP-30 effects on CBP/p300 in MM cells. Using capillary-based immunoassay analysis of p300 and CBP protein levels in the myeloma cell line MM1.S, we observed a dramatic potency shift in degradation activity of dCBP-30 compared with dCBP-1 (Figure 3A). Complete degradation of CBP and p300 was more rapid (2 h) compared with dCBP-1 with 10 nM dosing (Figure 3B). A methylated analog of dCBP-30, dCBP-30-bump, did not degrade CBP/p300, demonstrating the need for CRBN engagement (Figure S3). In a global proteomics assessment of MM1.S cells treated with dCBP-30 for 4 h, 10 nM CBP and p300 were the only proteins detected to be substantially decreased (Figure 3C; Table S3). To assess effects on CBP/p300 catalytic activity, we examined changes in the acetylation of known histone lysine substrates upon treatment with dCBP-1, dCBP-30, GNE-781, and A-485. Decreased acetylation of canonical substrates H3K27 and H3K18, and H2BK5 and H2BK20, acetylation of which directly correlates with transcriptional activity mediated by CBP/p300, were pronounced with dCBP-30 treatment (Figures 3D–3F).^38^ Interestingly, decreases in H2BK5ac and H3K18ac were observed only with catalytic inhibition whereas minor decreases in H2BK20ac and H3K27ac were also observed with bromodomain inhibition, confirming more selective effects of bromodomain inhibitors on CBP/p300 catalytic activity.^11^

**Figure 4.**
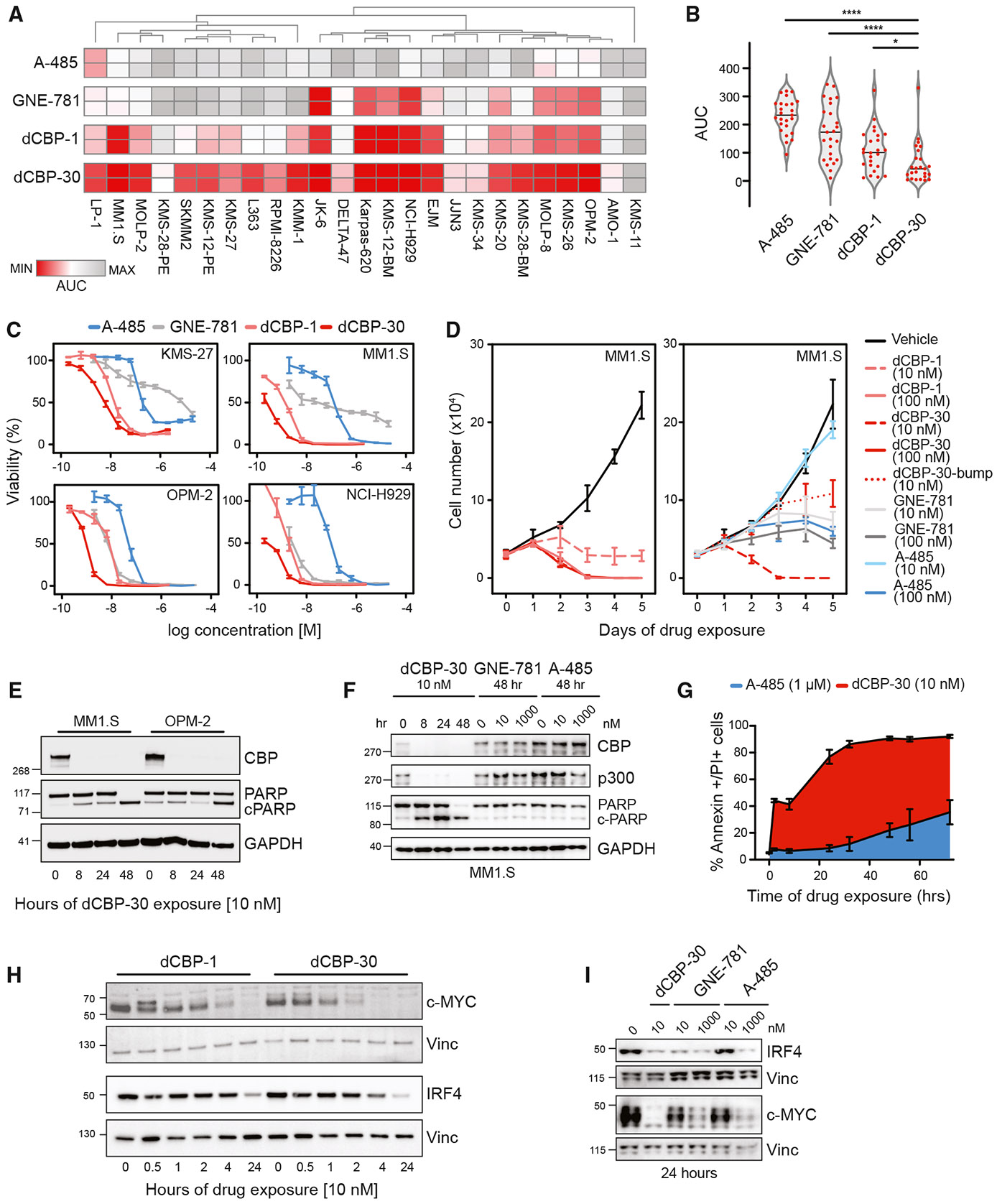
dCBP-30 has accentuated antiproliferative and pro-apoptotic activity in multiple myeloma cells (A) Clustered heatmap of A-485, GNE-781, dCBP-1, and dCBP-30 effects on the viability of a panel of 25 multiple myeloma cell lines, represented by the AUC of a nine-point dose-response ATP-based luminescence viability assay with five days of drug exposure. Clustering was performed by Euclidean distance using Morpheus (software.broadinstitute.org/morpheus). (B) Quantification of AUCs across cell line panel; ****p < 0.0001; *p < 0.05; line on each plot represents median. (C) Dose-response curves for each drug in four exemplary cell lines from (A): KMS-27, MM1.S, OPM-2, and NCI-H929. Data were normalized to DMSO-treated control cells; error bars represent ± SD of two replicates. (D) Growth-over-time in MM1.S cells comparing 10 nM and 100 nM of dCBP-1 and dCBP-30 (left) and dCBP-30 with the inhibitors A-485 and GNE-781 (right), normalized to DMSO-treated controls. Error bars represent ± SD of three replicates. (E) Immunoblotting of cleaved PARP (cPARP) in MM1.S and OPM2 cell lines following 10 nM dCBP-30 treatment. (F) Immunoblotting of cPARP in MM1.S cells comparing dCBP-30 with GNE-781 and A-485 (48 h). (G) Washout experiments in MM1.S cells comparing apoptosis induction by dCBP-30 (10 nM) versus A-485 (1 μM). Error bars represent ± SD of two replicates. (H) Immunoblotting of c-MYC and IRF4 following 10 nM dCBP-1 or dCBP-30 in MM1.S cells. (I) Immunoblotting of c-MYC and IRF4 comparing dCBP-30 with GNE-781 and A-485, 24 h. See also Figure S4.

In viability experiments across a panel of 25 MM cell lines, dCBP-30 demonstrated superior potency compared to dCBP-1 and the inhibitors A-485 and GNE-781 (Figures 4A–4C). dCBP-30 responses were more uniform, with a majority showing IC_50_ < 100 nM, while bromodomain inhibition with GNE-781 showed highly variable responses (Figure S4). In growth-over-time experiments, low doses of dCBP-30 (10 nM) were uniquely able to kill MM1.S cells, with almost complete cell killing observed by 72 h of treatment (Figure 4D). Apoptosis, as measured by cleaved PARP signal, was detected by 48 h, which was not the case for the inhibitors at up to 100-fold the dose of dCBP-30 (Figures 4E and 4F). Washout experiments indicated that apoptosis (as indicated with annexin+/PI+ staining) was observed in a stepwise fashion, with approximately 40% of cells apoptotic with 2 h of treatment, rising to ~80% with 24 h of dCBP-30 exposure (Figure 4G). This is consistent with a model previously demonstrated with CBP/p300 KAT inhibitors, whereby myeloma cells become refractory to re-acetylation following more than 24 h CBP/p300 blockade through the activity of lysine methylation machinery, reaching a phenotypic “point of no return”.^5^ Established transcriptional targets of CBP/p300 inhibitors and degraders, c-MYC and IRF4, were also more potently decreased with dCBP-30 compared with dCBP-1, GNE-781, and A-485 (Figures 4H and 4I). Together, dCBP-30 offers a more potent and efficacious CBP/p300 PROTAC for use in cellular studies, with superior activity to both established inhibitors and first-generation degraders in cell-based models of myeloma.

### CBP/p300 degradation leads to potent inhibition of critical MM signaling nodes

**Figure 5.**
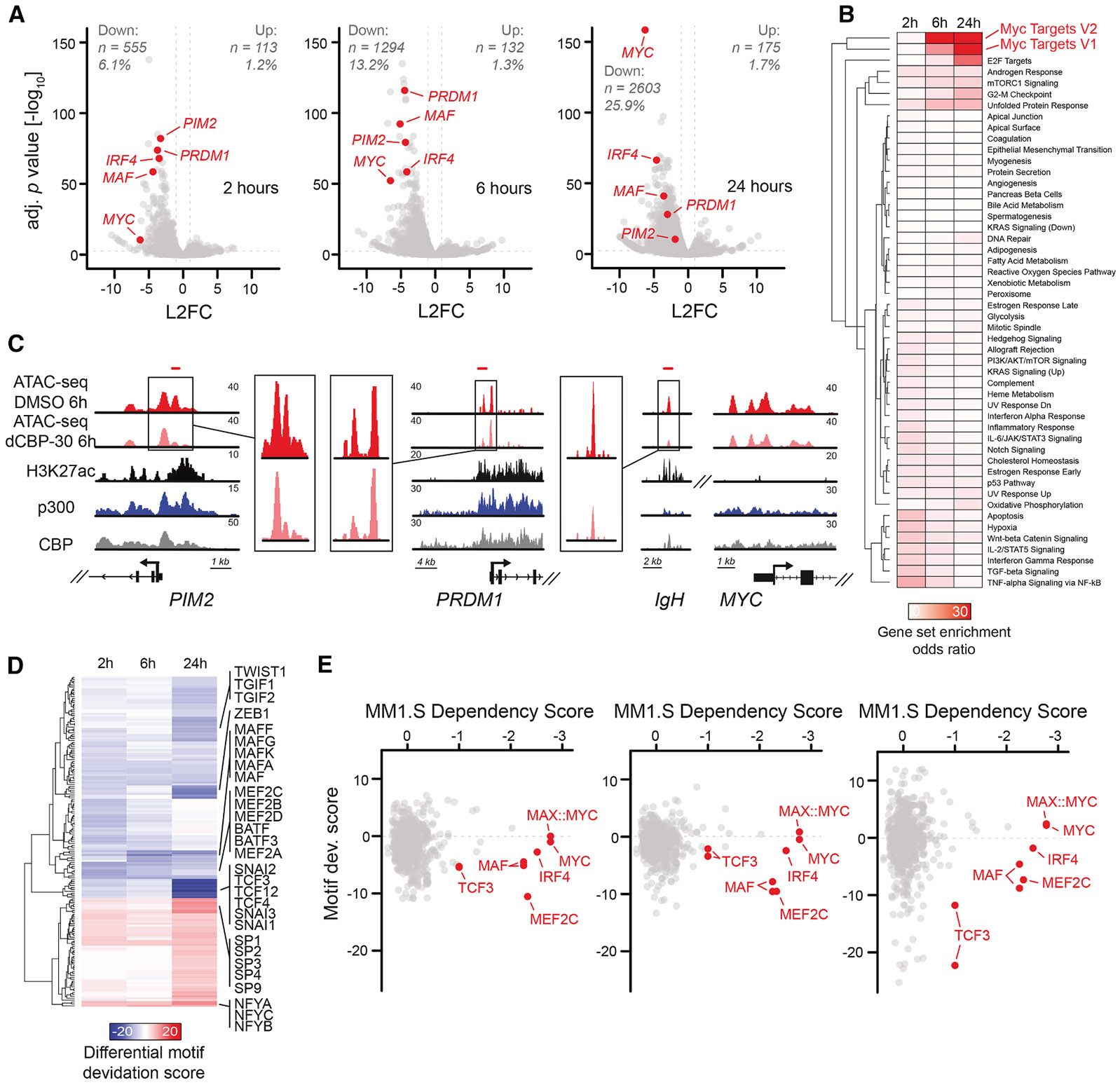
Comprehensive assessment of transcriptome and chromatin accessibility changes with short-term dCBP-30 treatment (A) Time-course volcano plots of RNA expression changes, as measured by SLAM-seq following dCBP-30 treatment (10 nM) in MM1.S cells. Highlighted are known dependencies downregulated by dCBP-30. L2FC, Log2 fold change. Cutoffs for gene quantification: *p* value > 0.1, L2FC > 1 or < −1. (B) Clustered gene set enrichment analysis of downregulated gene sets using the cancer “Hallmark 2020”, MSigDB. (C) Gene loci of downregulated MM dependency genes *PIM2* (chrX:48,915,839–48,921,927), *PRDM1* (chr6:106,071,093–106,096,202), and *IgH-MYC* (chr8:127,733,136–127,739,940 [*MYC*] and chr14: 105,698,000–105,703,490 [*IgH*]), with sites of decreased chromatin accessibility highlighted. Also shown are CBP, p300, and H3K27ac ChIP-seq tracks.^12^ (D) Clustered chromatin accessibility changes associated with TF motifs with dCBP-30 treatment highlight specific lost and gained TF motifs. (E) Correlation of motif accessibility changes and CRISPR-knockdown Chronos dependency scores in the MM1.S cell line (depmap.org). See also Figure S5 and Table S4.

CBP/p300 have been shown to directly interact with over 400 proteins and acetylate via their KAT function >21,000 lysines on >500 proteins, many of which are physically associated with transcriptional enhancer regions.^1^ Complete and acute CBP/p300 degradation likely affects the functioning of many of these enhancer factors. Chemical inhibition of CBP/p300 bromodomains has been shown to reduce *MYC* and *IRF4* expression in MM cells, decrease histone H3K27ac levels, and decrease chromatin accessibility when combined with IMID treatment.^11,39^ Treatment of MM1.S cells with A-485 results in very little chromatin accessibility changes yet decreases expression of critical MM driver genes including *MYC*, *IRF4*, *PIM2*, *MAF*, and *PRDM1*.^5^ Our previous analysis of transcriptional changes by RNA-seq with dCBP-1 treatment showed potent loss of *MYC* expression and decrease of chromatin accessibility at the 3′ regulatory region of the *IgH* enhancer that drives *MYC* expression in MM1.S cells and ~20% of MM cases.^12^ To gain a more complete picture of the overall effects of CBP/p300 degradation, we performed time-resolved measurements of newly synthesized mRNA using the SLAM-seq assay following 2, 6, and 24 h treatment of dCBP-30 in MM1.S cells. After two hours of treatment with dCBP-30 at a concentration sufficient to activate an apoptotic program, we observed a decrease in the expression of *MYC* but also in those of MM dependency genes *MAF*, *IRF4*, *PRDM1*, and *PIM2*. This expression pattern was sustained over 24 h, with downregulation of *MYC* expression and canonical MYC target genes after 24 h (Figures 5A and 5B; Table S4). Chromatin accessibility changes were modest, however, with 621 peaks displaying decreased accessibility 6 h following dCBP-30 treatment (of which approximately one-third are direct sites of CBP or p300 binding) (Figure S5; Table S4). These effects are similar to what has been observed with CBP/p300 KAT inhibitors, revealing that chromatin accessibility changes are not a general consequence of CBP/p300 degradation, despite clear effects on transcription.^5,40^ However, some genes with decreased transcription showed distinct loss of accessibility at their loci, including *PIM2* and *PRDM1*, along with the *IgH* enhancer that drives *MYC* expression in MM1.S (Figure 5C). A single promoter-proximal peak displayed decreased accessibility immediately upstream of *PIM2* and *PRDM1*, which might contribute to transcriptional decreases at these loci (Figure 5C). We globally assessed motif accessibility changes across a database of established TF position weight matrices (PWMs) and observed time-resolved TF activity pattern changes upon CBP/p300 degradation. This included immediate (2 h) loss of accessibility at MEF2 family and BATF family motifs, followed by loss of MAF motif accessibility (Figure 5D). Strong accessibility decreases at TCF, SNAI, and ZEB and gain in SP1/2/3/4/9 and NFY family motifs were observable after 24 h treatment, coinciding with cells having dramatically altered transcriptional profiles and the initiation of an apoptotic response (Figure 5D). Correlating motif accessibility changes with gene dependency in MM1.S cells from pooled CRISPR knockout screening (depmap.org) resolved how loss of specific TF signaling contributes to dCBP-30 effects with the initial decrease in MEF2C motif accessibility likely playing a significant causal role (Figure 5E). MEF2C directly binds to p300 and is a known substrate of p300 KAT activity, which can activate its DNA binding and transcriptional activity in myocytes.^41–43^ A similar regulatory interaction may also occur in MM and drive an as-of-yet unknown gene expression program that promotes cell viability. Among other TFs modulated by CBP/p300 degradation, *MAF* is a pioneer factor that cooperates with IRF4 in MM cells to promote proliferation and is overexpressed via an *IgH* rearrangement in MM1.S cells and in 3%–5% of patients with newly diagnosed MM with generally poor prognosis.^44,45^ TCF3 dependency is also characteristic of MM cells with ID2 downregulation and is among the most affected motif with dCBP-30 treatment at 24 h.^46^ These experiments reveal a clearer picture of the cascading and coordinated transcriptional and epigenetic consequences of CBP/p300 degradation in MM, with acute effects on MM-essential genes *PIM2*, *PRDM1*, *IRF4*, *MAF*, *MEF2C*, *TCF3*, and *MYC*. This highlights the multi-factorial nature of targeting CBP/p300 through degradation as a method to disrupt essential signaling nodes that drive MM.

### dCBP-30 is orally bioavailable and efficacious in mouse models of MM

**Figure 6.**
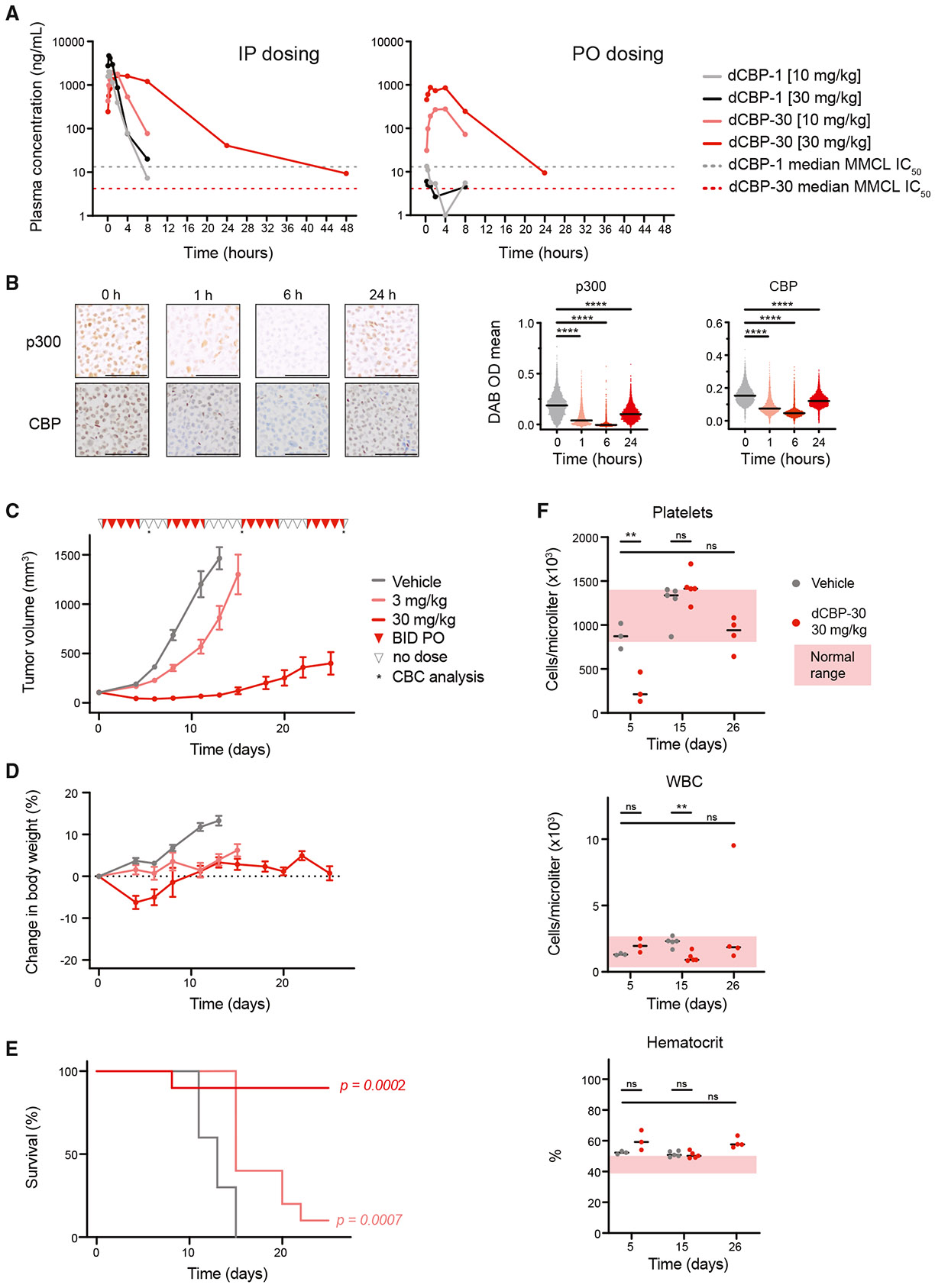
dCBP-30 is orally active and efficacious in an *in vivo* xenograft model of MM (A) Bioavailability in C57BL/6 mice, comparing dCBP-1 and dCBP-30 following intraperitoneal (IP) (left) or oral (PO) (right) dosing. Indicated is the median IC_50_ of dCBP-1 or dCBP-30 in MM cell line (MMCL) viability *in vitro* (from Figure 4A); *n* = 3 mice per treatment group. (B) Immunohistochemistry of CBP and p300 at time points indicated following a single dose of dCBP-30 (30 mg/kg) in NSG mice harboring subcutaneously xenografted NCI-H929 cells. Representative images of tumors shown at left, quantification of staining intensity at right (*n* = 4,000 randomly selected cells). ****p < 0.0001; scale bars represent 50 μm; line on each plot represents median. (C) Tumor volume of NCI-H929 engrafted mice treated intermittently with 3 or 30 mg/kg dCBP-30, BID; *n* = 10 mice per treatment group; error bars represent ± SEM. (D) Mouse weights of dCBP-30 treated mice from (C); error bars represent ± SEM. (E) Kaplan-Meier survival analysis of mice from (C); *p* values determined by log rank Mantel-Cox test. (F) Hematology analysis (platelets, white blood cells (WBCs), and hematocrit) of mice from (C) on days 5, 15, and 26 (endpoint) of study comparing the dCBP-30 (30 mg/kg) group with vehicle-treated control mice. Highlighted is the normal blood count range for NSG mice.^47^ **p < 0.01; line on each plot represents median. See also Figure S6 and Tables S5 and S6.

The increased potency of dCBP-30 prompted us to examine its capability in *in vivo* mouse models. Comparative pharmacokinetic profiling of bioavailability of dCBP-1 and dCBP-30 showed exposure times and concentrations by both intraperitoneal and oral dosing that would predict responses in tumor-bearing mice (Figure 6A; Table S5). Pharmacodynamic monitoring of CBP/p300 protein levels in both subcutaneously engrafted tumors (NCI-H929 cells) and somatic tissue (intestine) following dCBP-30 oral administration showed acute loss of both with maximal degradation observed at 6 h following treatment (Figures 6B and S6A–S6C). Using an intermittent dosing strategy, we observed a dose-response effect of dCBP-30 with twice-daily (BID) dosing. At 30 mg/kg, tumor regression from baseline was observed during the first two cycles of treatment (Figures 6C and S6D). The first cycle of 30 mg/kg dosing led to some weight loss that was recovered to baseline during subsequent cycles (Figures 6D and S6E). In the 30 mg/kg cohort, one animal required euthanization after the first cycle due to morbidity and body weight loss, yet the remaining surviving mice in the 30 mg/kg and 3 mg/kg cohorts had increased survival during the study compared with vehicle-treated control mice (Figure 6E). Complete blood count data were collected at day 5 (following first cycle), day 15 (immediately preceding third cycle), and day 26 (following fourth cycle). Reduction in platelets (thrombocytopenia) was observed at day 5 in 30 mg/kg mice cohort; however, platelets were generally normalized at days 15 and 26. No significant effects on white blood cells (WBCs) or hematocrit were observed (Figure 6F; Table S6). Collectively, dCBP-30 is orally bioavailable and shows efficacy in tumor growth inhibition in MM tumor-bearing animals.

### CBP/p300 degradation is synergistic with dexamethasone in myeloma

**Figure 7.**
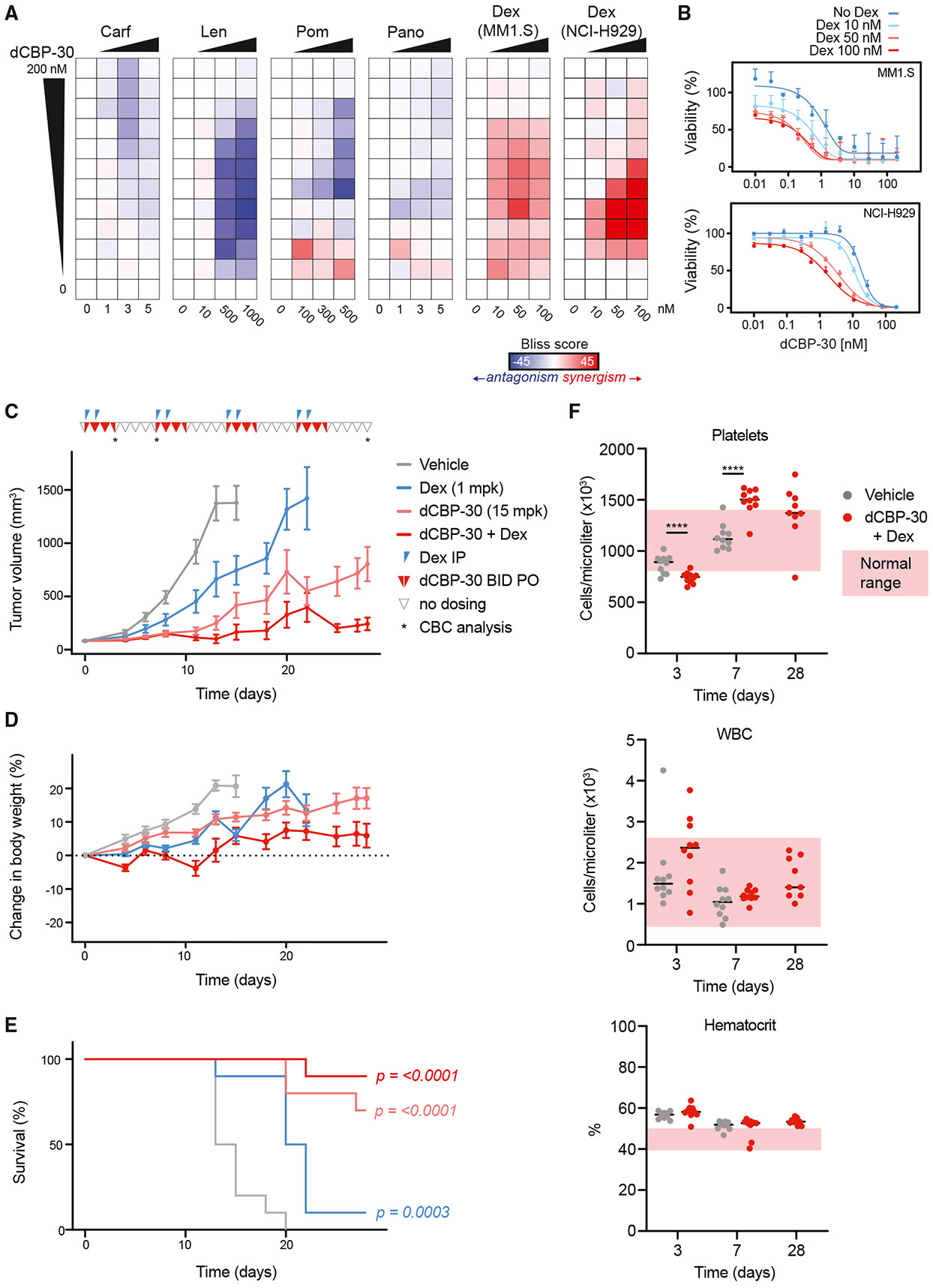
CBP/p300 degradation is synergistic with dexamethasone in MM cells (A) Synergy analysis of dCBP-30 in combination with carfilzomib (Carf), lenalidomide (Len), pomalidomide (Pom), panobinostat (Pano), and dexamethasone (Dex) in MM1.S (48 h) and NCI-H929 cells (+Dex, 120 h). (B) Dose-response curves of MM1.S and NCI-H929 cells combining dCBP-30 with dexamethasone (MM1.S, 48 h; NCI-H929, 120 h). (C) Tumor volume of NCI-H929 engrafted mice treated intermittently with dCBP-30 alone (15 mg/kg, BID), Dex alone (1 mg/kg, twice weekly), or in combination; *n* = 10 mice per treatment group; error bars represent ± SEM. (D) Mouse weights of treated mice from (C); error bars represent ± SEM. (E) Kaplan-Meier survival analysis of mice from (C); *p* values determined by log rank Mantel-Cox test. (F) Hematology analysis (platelets, white blood cells (WBCs), and hematocrit) of mice from (C) on days 3, 7, and 28 (endpoint) of study comparing the dCBP-30 + Dex group with vehicle-treated control mice. Highlighted is the normal blood count range for NSG mice.^47^ ****p < 0.0001; line on each plot represents median. See also figure S7 and Table S6.

With the promising efficacy of dCBP-30 in this model and the manageable toxicities observed, we sought to find treatment combinations with established MM clinical agents that may provide a synergistic response and opportunity for dose reduction of dCBP-30. We performed a drug synergy screen using established MM drugs, including proteasome inhibitor carfilzomib (Carf), immunomodulatory drugs lenalidomide (Len) and pomalidomide (Pom), HDAC inhibitor panobinostat (Pano), and glucocorticoid dexamethasone (Dex). As the mechanism of action of dCBP-30 requires proteasome activity, engagement with CRBN, and HDAC activity (required for complete acetylation loss following degrader-induced loss of KAT function), we were not surprised to find considerable drug antagonism with Carf, Len, Pom, and Pano treatments (Figure 7A). A considerable synergistic response was, however, observed with Dex (Figures 7A and 7B). We proceeded to test this combination *in vivo* using the xenografted NCI-H929 model. We halved the dose of dCBP-30 from 30 mg/kg to 15 mg/kg, and performed intermittent dosing (3 days on, 4 days off), with Dex administered twice weekly concomitant with dCBP-30 (Figure 7C). While Dex and dCBP-30 treatment alone had significant partial effects on tumor growth inhibition, their combination was superior with similar tumor regression as observed with 30 mg/kg of dCBP-30 (Figures 7C and S7A). Less weight loss was observed compared with 30 mg/kg dCBP-30 administration, with animals treated with the combination maintaining baseline weight during the first two cycles, but some weight gain was observed during cycle three (Figures 7D and S7B). Mice treated with this combination also demonstrated superior survival with one mouse succumbing to tumor burden during the final treatment cycle (Figure 7E). A slight reduction in platelets was observed in animals treated with the combination immediately following the first treatment cycle (day 3); however, these counts were fully recovered following the four-day treatment holiday (day 7) and were generally within normal range at day 28 (Figure 7F). No significant effects were observed in WBCs or hematocrit (Table S6). Additionally, no apparent toxicity was observed in an immunocompetent humanized CRBN model following four dosing cycles of dCBP-30, with similar CBP/p300 degradation effects in somatic tissue (Figures S7C–S7E).^48^ Together, dose reduction with dCBP-30 can be achieved with synergistic combination with dexamethasone, which could offer a larger therapeutic index for dual CBP/p300 degraders for anti-MM treatment.

## DISCUSSION

Targeting transcriptional coactivators CBP and p300 is an area of intense interest owing to their dual functions as acetyltransferases and scaffolding proteins essential for enhancer activity, transcription factor function, and oncogene expression.^49^ Pharmacological inhibition of their bromodomain or KAT domain has provided proof-of-concept that these proteins are druggable, and clinical utility of these modalities are currently under investigation in MM and other cancers (NCT07096778, NCT04068597, and NCT06403436). While chemical degradation of CBP/p300 has not yet entered clinical investigation, the data presented here demonstrate potential advantages for this modality for use in MM and perhaps other malignancies as well.

We show here that PROTAC-mediated degradation of CBP/p300 has unique pharmacologic properties and achieves superior antiproliferative potency compared with domain-directed inhibitors. Across a 300-cell line panel, the prototypical degrader dCBP-1 was consistently more effective at cell killing than the bromodomain inhibitor GNE-781 and the KAT inhibitor A-485. This finding aligns with the general principle that degraders can overcome disadvantages of inhibitors through their catalytic mechanism of action and comprehensive targeting of entire proteins rather than individual domain functions. Our lineage-focused analysis reveals that lymphoid malignancies are preferentially sensitive to CBP/p300 degraders, with MM emerging as the most vulnerable subtype. While MM cells are sensitive to both inhibitors and degraders, other lymphoid cancers such as diffuse large B cell lymphoma (DLBCL) displays heightened sensitivity to CBP/p300 degradation compared to inhibition, particularly bromodomain inhibition. This divergence highlights a potential limitation of bromodomain domain inhibition—partial suppression of CBP/p300 can fail to extinguish the enhancer activity that drives oncogenic transcription to the level required for cell death. Degraders, by contrast, demonstrate increased potency, likely through more effective disruption of enhancer-driven oncogenic circuits. Together, these results suggest that lymphoid cancers, MM in particular, are transcriptionally addicted to CBP/p300 in ways that degraders can maximally exploit.

Our development of dCBP-30 demonstrates that near-linkerless CRBN-based PROTACs can function as exceptionally potent degraders of CBP/p300. By directly conjugating the bromodomain inhibitor GNE-781 to thalidomide without an intervening structured linker that is typical of PROTACs, we obtained the lowest-molecular-weight CBP/p300 degrader reported to date. Surprisingly, dCBP-30 outperformed analogs incorporating flexible linkers or more structured linkers featured in clinical-grade PROTACs. This near-linkerless design led to unanticipated high potency of CBP/p300 degradation, inhibition of critical signaling nodes in MM, and oral bioavailability *in vivo*. While slight attenuation of a bromodomain-PROTAC-CRBN ternary complex is observed in cell-free assays, the increased potency highlights that minimizing compound size and increasing cell permeability are reliable strategies to create orally bioavailable degraders. Recent demonstrations of *in vivo* efficacy of an orally bioavailable CBP/p300 PROTAC that utilizes the same conjugation site as dCBP-1 having condensed linker geometry with oral delivery in prostate cancer models also support these observations.^29^ Collectively, these findings position dCBP-30 as a compact and pharmacologically potent CBP/p300 degrader within the emerging landscape of orally active degraders. We also hypothesize that direct thalidomide conjugation to protein-binding ligands may be applied to other targets with favorable solvent-exposed conjugation sites, removing the need for time-consuming iterative linker optimization. Deep structural analysis of the ternary complex induced by dCBP-30 will resolve the atomic-level interactions facilitated by this near-linkerless design.

dCBP-30 demonstrates greater depth of target degradation than either inhibitors or our first-generation degrader dCBP-1. In MM, dCBP-30 robustly downregulated canonical CBP/p300 targets such as MYC and IRF4 and blocked histone acetylation. Kinetic profiling using SLAM-seq and ATAC-seq further revealed inhibition or downregulation of dependencies unique to MM cells, including MEF2C, MAF, PIM2, and PRDM1. These factors have previously been implicated as MM-specific essential genes, and their early suppression by dCBP-30 suggests structural disruption of enhancer-driven gene expression programs. These effects may account for the superior cell killing activity of degraders compared with inhibitors. These targets have also been found to be decreased with CBP/p300 KAT inhibitors, suggesting that phenotypic consequences of CBP/p300 degradation are similar, although more potent.^5^ The increased potency may arise from more completely disrupting specific TF activities. Chromatin accessibility decreases at MEF2C sites, for example, are among the earliest changes observed following dCBP-30 treatment in MM cells. It is notable that in addition to being an acetylation target, the MEF2 TF family also binds directly to the p300 TAZ domain in a tripartite manner, representing a critical signaling node that may most effectively be blocked with a degrader. Moreover, we note that increased intracellular acetyl-CoA levels, which can be achieved through alterations in *PANK3*, lead to resistance to CBP/p300 KAT domain inhibition, which can be overcome by CBP/p300 degraders that target the bromodomain.^50^ In MM, resistance to IMIDs has been shown to be correlated with upregulation of BATF, blockage of which we observe as an acute effect of CBP/p300 degradation.^39^

Our prior efforts to characterize the efficacy of CBP/p300 degraders in MM models were limited due to poor pharmacokinetic properties of dCBP-1. In this study, *in vivo* evaluation of dCBP-30 in murine MM xenograft models demonstrated both tolerability and potent antitumor efficacy. While some weight loss and thrombocytopenia was observed, instituting drug holidays mitigated these effects, suggesting that some toxicity may be manageable with intermittent dosing strategies. These effects were most apparent during the initial treatment cycle, reflecting an acute and reversible perturbation of hematopoiesis rather than sustained bone marrow toxicity. These are similar to effects observed clinically with proteasome inhibitor treatment which are mitigated with specific intermittent dosing regimens.^51^ These findings suggest that optimized dosing schedules incorporating drug holidays may help maximize a therapeutic index for CBP/p300 degraders. Our drug combination studies revealed a complex interaction landscape—proteasome inhibitors, HDAC inhibitors, and IMIDs, all displayed anticipated antagonism when paired with dCBP-30. However, synergy with dexamethasone was observed *ex vivo*, enabling dose reduction of dCBP-30 and a partial mitigation of weight loss and thrombocytopenia while maintaining efficacy *in vivo*. These findings highlight the need to carefully map the pharmacological interactions of CBP/p300 degraders to identify compatible regimens.

Together, these data establish chemical degradation of CBP/p300 as a promising therapeutic strategy for enhancer-addicted malignancies such as MM. They also broaden the conceptual framework of PROTAC chemistry by demonstrating that compact, near-linkerless designs can yield highly potent degraders. dCBP-30 treatment overcomes pharmacological limitations of CBP/p300 inhibitors by collapsing enhancer-driven transcriptional dependencies at multiple levels in MM cells. This work positions CBP/p300 degradation as a powerful therapeutic approach for MM and other cancers dependent on enhancer-driven transcription. The preferential activity in lymphoid malignancies, particularly myeloma, provides a compelling rationale for translation of CBP/p300 degraders into the clinic as next-generation epigenetic therapy.

### Limitations of the study

We note some limitations of our work presented here. First, while the *in vivo* efficacy and pharmacokinetic/pharmacodynamic relationships we observed with dCBP-30 treatment in mice are encouraging, these studies were evaluated in a limited set of models and therefore, do not fully define a therapeutic index. Notably, transient hematotoxicity was observed early during treatment, highlighting the need for more comprehensive toxicology evaluation and dosing strategy optimization in higher rodent and other species. Future studies may also determine whether selective CBP or p300 degraders could preserve antitumor efficacy while improving tolerability and the therapeutic window relative to dual degraders, or whether specific tumor-targeting strategies through antibody conjugation may improve a therapeutic index. Additionally, although our multi-omics analyses establish the immediate epigenomic and transcriptomic consequences of CBP/p300 loss, longer-term cellular adaptation to sustained target depletion was not investigated. Addressing these questions will further clarify the translational potential of CBP/p300 degraders.

## STAR★METHODS

### EXPERIMENTAL MODEL AND STUDY PARTICIPANT DETAILS

#### Mouse models

Animals (*C57BL/6J, NOD.Cg-Prkdc^scid^ Il2rg^tm1WJI^/SzJ*) were housed at Syngene International, Ltd (Bangalore, India). *C57BL/6J* mice used for pharmacokinetic studies were male, 8-12 weeks old, 3 mice per group; *NOD.Cg-Prkdc^scid^ Il2rg^tm1WJI^/SzJ* were female, 6-8 weeks old, 10 mice per group. Female mice were used to enable group housing and ease of handling throughout the efficacy study. *C57BL/6-Crbn^tm2.1Ble^/J* mice were housed at the Mass General Hospital animal care facility. *C57BL/6-Crbn^tm2.1Ble^/J* were female, 4-5 weeks old, 3 mice per treatment group with one additional untreated animal. All animals were housed under a 12h/12h light/dark cycle, with free access to regular chow and water. Xenograft and pharmacokinetic studies were performed under IAEC protocol #1586/05-2024. Studies with *C57BL/6-Crbn^tm2.1Ble^/J* mice were conducted under the guidelines and approval of the Institutional Animal Care and Use Committee of Massachusetts General Hospital (MGH Animal Welfare Assurance No.: D16-00361; protocol #2017N000244). We note the limitations of using single-sex animal studies for these experiments; future *in vivo* characterization of advanced dCBP analogs in additional models will incorporate more sex-balanced cohorts to ensure sex-bias is not observed for this class of agent.

#### Cell lines

Cell lines are maintained as part of the MGH Center for Molecular Therapeutics for drug sensitivity profiling.^25^ All lines for the 300-cell line screen were tested for mycoplasma and grown in either RPMI or DMEM/F12 media with 10% fetal bovine serum. List of cell lines can be found in Table S1. For myeloma cell line screen, cells were cultured in RPMI medium with 10% FBS (MM1.S, MM1R, U266, NCI-H929, RPMI-8226, KMS-11, KMS-12-BM, KMS-12-PE, KMS-28-BM, KMS-28-PE, DELTA-47, KMS-20, KMS-26, KMS-27, KMS-34, KMM-1, OPM-2, L363) or 20% FBS (MOLP-2, MOLP-8, JK-6, JJN3, AMO-1, Karpas-620, SKMM2); or with IMDM media supplemented 10% FBS (EJM) or 20% FBS (LP-1). HAP1 CRBN knockout cells were grown as described previously.^12^ All cell lines were cultured at 37°C with 5% CO_2_.

### METHOD DETAILS

#### Cell line viability screening, dose-response analysis

Cells were seeded into 384-well plates into duplicate plates one day prior to drug addition. Cells were treated with vehicle (DMSO) or compound stocks dissolved in DMSO with a PerkinElmer JANUS workstation. Treatments of each compound were performed at 9 dose levels [GNE-781, A-485: 20 μM, 6.3 μM, 2 μM, 0.63 μM, 0.2 μM, 0.063 μM, 0.02 μM, 0.0063 μM, 0.002 μM; dCBP-1: 2 μM, 0.63 μM, 0.2 μM, 0.063 μM, 0.02 μM, 0.0063 μM, 0.002 μM, 0.00063 μM, 0.0002 μM). Following drug addition, cells were incubated for 5 days. Cell viability was then determined using the CellTiter-Glo assay (Promega), with luminescence measured by a PerkinElmer Envision plate reader. Viability was determined as the normalized luminescence to DMSO control for each cell line (n=2 replicates). For 300-cell line screen, dose-response curves were fitted to a four-parameter log-logistic model using SciPy scipy.optimize.curve_fit to estimate IC_50_, AUC, *E*_max_, and *HS* values. AUC values were calculated over the shared dose range screened for each compound (2 μM - 0.002 μM). For myeloma cell line screen, comparative AUC values were calculated using GraphPad PRISM. For growth-over-time assays, MM1S cells were plated at a density of 3 x 10^5^ cells/mL in 24-well low-attachment tissue culture plates. One day following seeding, cells were treated in triplicate cultures were counted manually by hemocytometer over the course of five days of treatment.

#### HiBiT assays

HAP1-HiBiT cells were thawed in IMDM (GIBCO 12440-053) + 10% FBS (GIBCO A52567-01) + 1% PenStrep (GIBCO 15140-122) media and grown for two days until seeding. On the day of seeding, cells were trypsinized using TrypLE (Thermo Scientific 12604039), resuspended in IMDM containing 1% PenStrep and 2% FBS, and seeded in 384 well plates (Corning #3570) at 1.7 x 10^5^ cells/mL, 30 μL well. Cells are incubated at 37°C, 5% CO_2_ overnight. The following day 20 μL of a 1% Endurazine solution in IMDM is added to each well and incubated for 3 hours at 37°C, 5% CO_2_. Compounds were delivered to the assay plate with a JANUS workstation pintool and immediately sealed with a clear plate seal (PerkinElmer #6050185) and loaded onto an Envision plate reader with temperature control module set to 35°C. Luminescence readings were taken at regular intervals (every three minutes for the first hour, every ten minutes thereafter) for just over 24 hours. Readings for each compound were obtained in triplicate and averaged values were normalized to vehicle-treated wells and baseline timepoint. Degradation quantification was performed by fitting a one-phase decay model (GraphPad Prism) to determine D_max_, DC_50_, degradation rate (DR), and area-over-the curve.

#### Capillary immunoassays and immunoblotting

For immunoassays, cells were plated into 6-well plates followed by overnight incubation before treatment. For non-histone assays, cells were harvested and lysed in RIPA buffer containing HALT protease inhibitor cocktail (Pierce). Protein extraction was facilitated by passing all samples through a Gauge 28 Micro-Fine IV insulin syringe (BD). Capillary-based immunoassays were performed using a standard WES (Simple Western) protocol (proteinsimple). Lysates were loaded onto WES plates at 0.8 mg/mL total protein. For histone immunoblots, cells were resuspended and washed twice in 0.5% Triton X100 (V/V) in 1X PBS containing HALT protease inhibitor cocktail and incubated overnight in 0.2N HCl at 4°C. Samples were centrifuged and supernatant collected, neutralized with 2M NaOH. For FFPE lysate preparation, small tissue chunks were retrieved from FFPE blocks, incubated at 60°C for 10 minutes to remove excessive paraffin, followed by xylene incubation for deparaffinization. Deparaffinized tumors were then rehydrated with gradual decrease of ethanol from 100% to 70%, followed by PBS at the end. Rehydrated tumors were homogenized and lysed in buffer (500 mM Tris, 1mM EDTA, 2% SDS, 150 mM NaCl, 0.5% sodium deoxycholate, 1% NP-40) supplemented with HALT protease inhibitor cocktail. Initial reverse cross-linking and extraction was performed by incubating at 95°C for 30 min and 80°C for 2 hours, respectively. Samples were passed through an insulin syringe to ensure lysis and to shear DNA. WES staining or immunoblotting was conducted using the following antibodies: Vinculin (Bethyl, A302-535A), CBP (Cell Signaling, D6C5), p300 (Cell Signaling, D2X6N), GAPDH (Cell Signaling, 14C10), CRBN (Sigma, HPA045910), MYC (Santa Cruz, 9E10), BRD4 (Bethyl, A301-9851), IRF4 (Cell Signaling, P173), PARP (Cell Signaling, 9542), H2BK5ac (Cell Signaling, D5H1S), H2BK20ac (Cell Signaling, D7O9W), Histone H2B (Active Motif, 39210), H3K27ac (abcam, ab4729), H3K18ac (active motif, 39755), H3 (Cell Signaling, 96C10) .

#### AlphaScreen ternary complex assay

Recombinant FLAG-CRBN/DDB1/CUL4A/RBX1 (BPS Bioscience, #100329) and bromodomain (GST-CBP, BPS Bioscience, #31128; His-p300, BPS Bioscience, #31118) were diluted to 7.3 ng/μL and 0.83 ng/μL respectively in 1x Immuno Buffer (BPS Bioscience, #79311) and 25 μL of protein mixture was add to each well of a 384 well AlphaPlate (PerkinElmer). Compounds were then added at 30 nL per well from DMSO stock plates using a JANUS workstation (PerkinElmer) pin tool. Compounds were incubated with proteins for one hour at room temperature. Next, 12.5 μL of AlphaLISA anti-FLAG acceptor beads diluted 250-fold (PerkinElmer, #AL1112C) was added to each well and incubated with gentle shaking at room temperature for 1 hour. Then, 12.5 μL of either AlphaScreen glutathione donor beads (for CBP assay, PerkinElmer, #6765300) or nickel chelate donor beads (for p300 assay, PerkinElmer, #AS101D) diluted 125-fold was added to each well, the plate was gently shaken for 30 minutes at room temperature and then read on a PerkinElmer Envision plate reader.

#### PAMPA membrane permeability assay

Assay was conducted at Syngene International, Ltd (Bangalore, India) using the pION PAMPA double sink system (GIT-PAMPA). PRISMA_HT_ was diluted in Milli-Q water and pH adjusted to 4.0, 5.0, 6.5, and 7.4 with 2N NaOH. dCBP-1 and dCBP-30 were diluted in pION donor buffer to a final concentration of 10 μM and added to individual wells of GIT-PAMPA donor plate. Then, 5 μL GIT-O lipid was added to the back side of acceptor plate followed by 200 μL of acceptor sink buffer. Plate was covered and incubated for 4 hr at room temperature in a humidified atmosphere. Sandwich plates were then separated, and 200 μL of both acceptor and donor solutions were transferred to fresh 96-well deep well plates. Samples were mixed with acetonitrile containing an internal standard (tolbutamide), plates were covered and vortexed for 5 minutes at 1000 rpm, then centrifuged at 4000 rpm for 10 minutes; 100 μL of each sample were transferred to a new plate, diluted with 100 μL Milli-Q water and then analyzed by LC-MS/MS.

#### Global proteomics

Frozen cell pellets were lysed by the addition of 500 μL of lysis buffer (75 mM sodium chloride, 10 mM sodium pyrophosphate, 10 mM sodium fluoride, 10 mM B-glycerophosphate, 10 mM sodium orthovanadate, 50 mM EPPS pH 8.5, cOmplete Mini EDTA-free Protease Inhibitor Cocktail, 3% SDS, 5 mM PMSF). Disulfide bonds were reduced by the addition of dithiothreitol (DTT) to a final concentration of 5 mM with incubation at 56°C for 30 minutes, then alkylated with iodoacetamide (IAA) to a final concentration of 15 mM followed by incubation in the dark at room temperature for 20 minutes. The reaction was stopped by the addition of DTT to a final concentration of 5 mM with incubation in the dark at room temperature for 20 minutes.

Proteins were precipitated using a standard trichloroacetic acid (TCA) protocol by adding one part TCA to four parts protein solution, followed by incubation on ice for 10 minutes. The precipitated protein was pelleted by centrifugation (15,000 g, 10 min, 5°C) and washed twice with prechilled acetone (−20°C, 300 μL, 15,000 g, 10 min, 5°C). The remaining protein pellets were resuspended in 500 μL 1 M urea, 50 mM EPPS (pH 8.5), 10 mM calcium chloride and digested overnight at room temperature with 1 μg/μL endoproteinase Lys-C (Wako) followed by a digestion with sequencing-grade trypsin (Promega) at a final concentration of 1 ng/μL for 6 hours at 37°C. The digestion was quenched with 1% trifluoroacetic acid (TFA), and peptides were desalted using Sep-Pak C18 solid-phase extraction (SPE) cartridges (Waters). The peptide concentration of each sample was determined using a BCA assay (Thermo Scientific).

For labeling with TMTpro reagents (Thermo Scientific), 20 μg of peptides were dried and resuspended in 25 μL of 200 mM EPPS (pH 8.5), 30% acetonitrile (ACN). Labeling was performed by adding 100 μg TMT reagent in anhydrous ACN and incubating at room temperature for 1 h. The reaction was stopped by addition of 5% (w/v) hydroxylamine in 200 mM HEPES (pH 8.5) to a final concentration of 0.5% hydroxylamine and incubation at room temperature for 15 min. Samples were acidified with 1% TFA, and samples were combined. The pooled samples were desalted using Sep- Pak C18 SPE cartridges.

Samples were analyzed on an Orbitrap Eclipse mass spectrometer (Thermo Fisher Scientific) coupled to an EASY-nLC 1200 HPLC and autosampler (Thermo Fisher Scientific) and equipped with a FAIMS Pro^™^ Interface (Thermo Fisher Scientific). Peptides were separated on an in-house packed microcapillary column (30 cm length; 150 μm inner diameter; 360 μm outer diameter) containing GP-C18 beads (1.8 μm, 120 Å, Sepax Technologies). Data were acquired using an in-house-developed method for global targeted proteomics of human proteins (UniProt UP000005640 extended with human UniParc proteins) implemented through the application programming interface (API) provided by Thermo Fisher Scientific. The method involved acquiring MS2 spectra upon peptide fragmentation with CID at 30% normalized collision energy in the Orbitrap at a resolution of 50 x 10^3^ following collision-induced dissociation (CID). From each MS2 scan, up to ten of the most intense product ions were selected for subsequent MS3 analysis. Peptide-spectral matches were made in real-time using a custom algorithm and validated post-acquisition with a target-decoy search strategy to ensure a false discovery rate (FDR) of ≤1%.^60^ Quantitative MS3 spectra were acquired in the Orbitrap at a resolution of 50,000. For quantification, TMT reporter ion intensities were extracted from the collected MS3 spectra by selecting the most intense signal within a 0.03 Th isolation window centered on the expected m/z for each reporter ion. All mass spectrometry RAW data can be accessed through the MassIVE data repository (http://massive/ucsd.edu/ProteoSAFe) under the accession number MSV000101009.

#### Washout AV/PI staining

MM1.S cells were seeded at 1 x 10^5^ cells/mL in 48-well tissue-culture plates and treated with either A-485 (1 mM) or dCBP-30 (10 nM). Cells were harvested (including TrypLE to collect adherent population) at timepoints of 2-, 8-, 24-, 32-, 48-, 56- and 72-hours post-treatment, washed in PBS and pelleted at 450 x *g* in an Eppendorf benchtop centrifuge. Cells were resuspended in 500 μL fresh media and replated for the remainder of the 72-hour period in the absence of drug, after which they were harvested and assessed for Annexin-V/PI positivity by flow cytometry.

For AnnexinV-APC/PI staining, harvested cells were washed in PBS and pelleted, prior to resuspension in Annexin-V binding buffer (10 mM HEPES, 140 mM NaCl, 5 mM CaCl_2_) containing APC-conjugated Annexin-V (1:100, BD Biosciences 550475) and propidium iodide (0.1 μg/mL). Cells were incubated at room temperature for 15 minutes prior to analysis on a FACSCanto II flow cytometer (BD Biosciences).

#### SLAM-seq

SLAM-seq was performed as described with triplicate samples per treatment condition.^52^ MM1.S cells were pre-seeded in 6-well plates (1.5 x 10^6^ cells at 0.75 x 10^6^ cells/mL) 16 hours prior to treatment with DMSO or dCBP-30 (10nM) for 2-, 6-, and 24-hours. One hour prior to harvest, cells were labeled with 4-thiouridine (4sU, 100 μM), and for harvest cells were lysed with TRIzol^™^ reagent (600 μL/sample). To isolate total RNA, TRIzol^™^ lysates were mixed with chloroform (180 μL) and centrifuged at 4°C (16000 g, 15 minutes) prior to isolation of the nucleic acid containing aqueous layer. Aqueous supernatants were supplemented with DTT (0.1 mM final concentration), GlycoBlue^™^ co-precipitant (15 μg), and 1x volume 100% (v/v) isopropanol, and vortexed at room temperature (RT) for one minute. Samples were incubated at RT for 10 minutes followed by centrifugation at 4°C (20000 g, 20 minutes). Precipitated RNA pellets were washed with 75% (v/v) ethanol prior to air-drying at RT. RNA pellets were reconstituted in 1 mM DTT at 55°C for 10-minutes prior to storage on ice. Prior to iodoacetamide (IAA) conversion 0.5 μg RNA isolated from *Drosophila melanogaster* S2 cells incubated with 4sU (100 μM, 1 hour) was mixed with 10 μg human/MM1.S RNA (5% external spike-in). IAA conversion was performed at 50°C for 15-minutes, with each reaction containing 10.5 μg RNA, 10 mM IAA, 50 mM NaPO_4_ pH 8, and 50% (v/v) DMSO. Reactions were quenched by the addition of DTT (20 mM final concentration). To isolate RNA, quenched reactions were supplemented with GlycoBlue^™^ co-precipitant (15 μg), NaOAc pH 5.2 (300 mM) and 2.5x volume 100% (v/v) ethanol, followed by vortexing for one minute, incubation at −80°C for 30-minutes, and centrifugation at 4°C (20000 g, 20 minutes). Precipitated RNA pellets were washed twice with 75% (v/v) ethanol prior to air-drying at RT. RNA pellets were reconstituted in TRIzol^™^ reagent (300 μL/sample) prior to isolation using the Direct-zol RNA Miniprep kit (Zymo Research, R2051) as per manufacturer’s instructions. RNA-seq library preparation was performed using the Lexogen Quantseq 3′mRNA-Seq V2 Library Prep Kit FWD, and single-end 100bp sequencing (25-30 x 10^6^ reads/sample) was performed using the Illumina NextSeq 2000 (Molecular Genomics Core, Peter MacCallum Cancer Centre).

Raw files were trimmed with TrimGalore! (v.0.4.4)^54^. Trimmed reads were then processed with Slamdunk workflow v.0.4.3^2^ with default parameters, and mapped to UTRs from human genome (hg38), accessed from R package TxDb.Hsapiens.UCSC.hg38.knownGene.^61^ After collapsing reads per transcript with alleyoop function *collapse*, gene counts were processed with R v.4.4.2. Out of the total reads 93-94% were mappable to the genome, and 80-81% retained after removal of multimapping and low-quality reads. Differential gene expression was performed with DESeq with cutoffs for fold change of 2x, and FDR of 0.05.^53^ Pathway enrichment analysis was performed with EnrichR using database “MSigDB Hallmark 2020”.^54^ Gene enrichment was performed with R package *fgsea*.^62^ Data is accessible at the NCBI GEO (GSE318522).

#### ATAC-seq

Fifty thousand MM1.S cells treated with DMSO or dCBP-30 (10 nM) for 2-, 6-, or 24-hours in quadruplicate. Each sample was washed with 1 mL cold PBS and then resuspended in 50 mL cold Omni-ATAC lysis buffer (10 mM Tris-HCl, pH 7.5, 10 mM NaCl, 3 mM MgCl_2_, 0.1% v/v NP-40, 0.1% v/v Tween-20, 0.01% v/v digitonin) with *Drosophila* cell nuclei spike-in controls (Active Motif, #53154), incubated on ice for 3 min, then resuspended in 1 mL Omni-ATAC wash buffer (10 mM Tris-HCl, pH 7.5, 10 mM NaCl, 3 mM MgCl_2_, 0.1% v/v Tween-20). Nuclei were pelleted by centrifugation and resuspended in 50 mL Omni-ATAC transposition reaction mixture (25 mL Nextera TD buffer, 16.5 mL PBS, 0.1% v/v Tween-20, 0.01% v/v digitonin, 2.5 mL Tn5 transposase) and incubated at 37°C for 30 minutes. DNA was purified using a MinElute PCR purification kit (Qiagen). Libraries were PCR amplified (15 total cycles) and were purified and sequenced using 150 bp paired end reads on an Illumina Novaseq 6000 (Novogene). Adapters were trimmed with TrimGalore! (v0.6.7) and were aligned to the reference human genome (hg38) and the *Drosophila* reference genome (dm3) with Bowtie2 (-X 2000 -N 1 -L 20 -i S,1,0.50 –local –rdg 5,1 –rfg 5,1 -D 15 -R 3).^55,63^ Unmapped/low quality and chrM reads (mapQuality ≥ 30; isProperPair; !chrM) were filtered with BAMtools (v2.5.2); reads in blacklisted regions (ENCFF356LFX_blacklist_hg38.bed) were removed with NGSUtils (v0.5.9); and duplicated reads were removed with Picard MarkDuplicates (v3.1.1.0). Normalization factors were determined using the dm3-mapped read counts from spiked-in nuclei per manufacturer’s instructions, and each the hg38-mapped reads from each sample were downsampled accordingly using Picard downsample (v3.1.1.0). Peak calling was performed using MACS2 callpeak (–nomodel –extsize 200 –shift -100; q-value cutoff 0.1) (v2.2.9.1).^56^ Peaks from each replicate were combined using Bedtools intersect and Bedtools merge function was used to generate a master list of peaks across samples (v2.31.1).^64^ Read counts for each peak in each sample was determined using featureCounts (v.2.0.8).^65^ Differential peak intensities between dCBP-30-treated and DMSO vehicle-treated control samples was performed using edgeR (v3.36.0).^66^ For peak track visualization, mean intensity signal tracks for each sample were generated using deepTools bigwigCompare tool (v3.5.4).^67^ To intersect ATAC-seq peaks with p300 and CBP binding sites, our previously reported ChIP-seq datasets were used.^12^ However, read counts from these datasets were too low to establish high-quality peak lists, so the ChIP experiments were repeated and sequenced as previously performed. Raw read processing and alignments were performed as above. Peaks were called with MACS2 callpeak (–nocontrol –single-end –nomodel –extsize 200 –shift 0; q-value cutoff 0.1. To determine TF motif accessibility, we used the chromVAR software package with the JASPAR2020 motif database.^59,68^ This analysis was performed specifically on ATAC-seq peaks that overlapped with MM1.S CBP or p300 binding sites as determined by ChIP-seq. Data is accessible at the NCBI GEO (GSE318521).

#### *In vivo* pharmacokinetics

dCBP-1 and dCBP-30 were formulated in 30% (v/v) polyethylene glycol 400 (PEG400) and 70% (v/v) Captisol^®^ solution (20% w/v in water, final concentration 14% (w/v)). Light sonication was used to achieve a homogeneous solution. Male 8-12-week-old C57BL/6 mice were dosed by intraperitoneal (IP) injection or per oral (PO) delivery, three mice per treatment group. Serial blood samples were collected at indicated time points from the saphenous vein. Blood samples were centrifuged (13,000 rpm, 5 minutes, 4°C), and plasma collected. Five microliters of each plasma sample was precipitated with 150 μL acetonitrile containing internal standard, vortexed at 1000 rpm for 10 minutes, centrifuged for 10 minutes at 4000 rpm, and run on an API-4500 LC-MS/MS with Shimadzu Nexera X2 HPLC, Kinetex C18 column (5mM ammonium acetate/0.1% formic acid in acetonitrile gradient) for compound quantification. Pharmacokinetic parameters were calculated using non-compartmental analysis module of Phoenix WinNonlin (v8.2). All studies were performed at Syngene International, Ltd (Bangalore, India).

#### *In vivo* xenograft studies

Two million NCI-H929 cells resuspended in 50% Matrigel were injected subcutaneously into 6–8-week-old female NSG (NOD.Cg-*Prkdc^scid^ Il2rg^tm1Wjl^*/SzJ) mice (Jackson Labs, strain #005557). Tumor volume was quantified by caliper measurements using the following formula: *Tumor Volume* = *Length[longest dimension] x (Width[shortest dimension])^2^*/2, and mice were randomized into treatment groups.

For pharmacodynamic study of dCBP-30 effects on CBP/p300 protein levels, mice were dosed once with 30 mg/kg dCBP-30 by oral gavage when tumor volume reached >200 mm^3^. Mice were euthanized with CO_2_ at predetermined time points, and tissue (tumor and intestine) was immediately collected and fixed for immunohistochemistry analysis as described below.

For efficacy studies of dCBP-30, mice were dosed with an intermittent dosing schedule of dCBP-30, twice per day by oral gavage. For the single agent, three-arm study testing dCBP-30 at 3 and 30 mg/kg, mice were dosed with a 4 days on-drug/3s day off-drug schedule (cycle 1); 4 days on/4 days off (cycle 2); 4 days on/3 days off (cycle 3). Four more days of dosing (cycle 4) was performed, and the study was terminated following the final dosing day.

For the combination study (dCBP-30 with Dex), mice were dosed with 15 mg/kg of dCBP-30, twice per day by oral gavage. This study included four cycles of dosing dCBP-30, 3 days on-drug/4 days off-drug. Dexamethasone was delivered at 1 mg/kg by IP injection on days 1 and 2 of each cycle (twice weekly). Blood was collected from selected mice at indicated time points throughout each study for hematologic cell count analysis. Xenograft animal studies were carried out at Syngene, International (Bangalore, India) under IAEC protocol #1586/05-2024.

#### *In vivo* activity in humanized CRBN mice (*Crbn^V380E/I391V^*)

Four- to 5-week-old female humanized CRBN mice (*C57BL/6-Crbn^tm2.1Ble^/J*, Jackson Labs #035831) were randomized to treatment groups. For tolerability study, mice were dosed twice daily with 3 days on/4 days off schedule for 4 weeks by oral gavage. Body weight was measured daily, and cardiac blood was collected at the end of study to measure complete blood counts (CBC) using an Element HT5 analyzer (Heska). For pharmacodynamic assessment of dCBP-30 activity, mice were given a single dose by oral gavage of either 15 mg/kg or 30 mg/kg. Mice were euthanized with CO_2_ after six hours, and tissue (tumor and intestine) was immediately collected and fixed for immunohistochemistry analysis as described below.

#### Immunohistochemistry

Tissues were fixed in 10% neutral buffered formalin for 24 h at room temperature and then kept in 70% ethanol, followed by dehydration and paraffin embedding. IHC was carried on the automated Ventana DISCOVERY ULTRA platform with anti-CBP (clone EPR23418-23, abcam #ab253202) and anti-p300 (clone D8Z4E, Cell Signaling Technology #86377). Epitope retrieval was done with either DISCOVERY CC1 (06414575001, Roche) and CC2 (05279798001, Roche), and epitope block was done with S-block (760-4212, Roche). DISCOVERY OmniMap anti-Rabbit HRP (760-4311, Roche) was used for secondary antibody incubation, DISCOVERY ChromoMap DAB kit (760-159) was used for chromogenic reaction, and Bluing reagent (760-2307, Roche) was used to counterstain. Images were quantified using the positive cell detection tool of QuPath software (version 0.6.0).^57^

#### Drug combination synergy screening

Cells were plated into 384-well plates with 3,000 cells per well (NCI-H929) or 4,000 cells per well (MM1.S) in 30 μl of appropriate media 24h prior to drug administration. Cells were treated with multiple doses of dCBP-30, carfilzomib (HY-10455, MedChem Express), lenalidomide (SML2283, Sigma), pomalidomide (P0018, Sigma), panobinostat (S1030, Selleck Chemicals), or dexamethasone (S1322, Selleck Chemicals) resuspended in DMSO using D300e Digital Dispenser (30100152, Tecan). Cells were incubated for 2, 3, and 5 days and the viability was determined by measuring relative ATP levels using CellTiter-Glo (G8462, Promega). In brief, CellTiter-Glo solution was added to each well, the luminescence was read on the EnVision Multi-well plate reader (2104-0010, PerkinElmer), and relative viability were generated by normalizing luminescence signal to DMSO-treated wells. For synergy analysis, individual values of relative cell viability from each well were analyzed using SynergyFinder.^58^ The synergistic effect of drug combination was determined with Bliss synergy score.

#### Synthesis and characterization of dCBP analog library

##### General methods and materials

All reactions were conducted under Argon (Ar) atmosphere, unless otherwise mentioned. All commercially available reagents and solvents were used as such without further purification. Reactions were either monitored by TLC (silica gel HL, w/UV254, 250 mm) and visualized either under UV lamp or by charring with ceric ammonium molybdate or potassium permanganate stain solutions or an Agilent 1260 system (UV detection at 220, 254 and 280 nm) coupled to an Agilent Technologies 6130 Quadrupole MS system using the following methods: Mobile Phases: A: H_2_O (+ 0.1% formic acid, v/v) B: CH_3_CN (+ 0.1% formic acid, v/v) Method 1: Column: Phenomenex Luna, C18 (2), 5 mm, 100 X 2 mm column, flow rate: 0.7 mL min-1 using Method-1: Mobile Phase B: 5 to 95% for 7 min. Flash chromatography was performed on a Combi flash (ISCO) instrument using prepacked commercially available C18 and silica gel columns. ^1^H NMR and ^13^C NMR spectra were recorded on either a 500 MHz Varian or 400, 300 MHz Bruker Avance III spectrometer. NMR data were obtained in either CDCl_3_ or CD_3_OD solvents. Data for ^1^H NMR are reported as follows: chemical shift value in ppm, multiplicity (s = singlet, d = doublet, t = triplet, dd = doublet of doublets, and m = multiplet), integration value, and coupling constants are given in hertz (Hz). Chemical shifts referred to (CH_3_)_4_Si (δ_H_ 0.00 ppm) or the residual peak in either CDCl_3_ (δ_H_ 7.26 ppm) or CD_3_OD (δ_H_ 3.31 ppm). HRMS was performed on Agilent 6210 TOF-LC/MS. NMR characterization provided in Data S1.

##### Synthesis of dCBP-1, dCBP-2, dCBP-3, dCBP-25 and dCBP-26 and dCBP-27


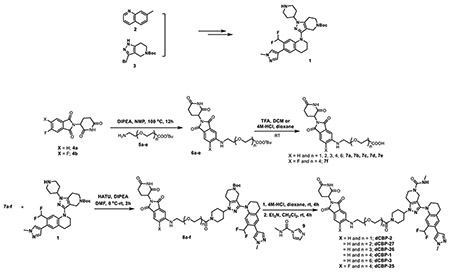


###### Synthesis of tert-butyl 3-(7-(difluoromethyl)-6-(1-methyl-1H-pyrazol-4-yl)-3,4-dihydroquinolin-1(2H)-yl)-1-(piperidin-4-yl)-1,4,6,7-tetrahydro-5H-pyrazolo[4,3-c]pyridine-5-carboxylate (1):

Synthesis of compound **1** was performed using starting materials **2** and **3** according to reported literature procedures.^12,30^

###### General procedure for synthesis of linker (6a-e):

Synthesis of compound **6a-e** was performed using starting materials **4a and 4b** and linkers **5a-e** respectively according to literature reported procedure and spectral data were satisfactory as literature reports.^12,69,70^

###### General procedure for synthesis of linker 7a-f:

Synthesis of compound **7a-f** was according to reported literature procedure and crude product was used in the next reaction step without further purification.^12,70^

###### General procedure for synthesis of compound 8a-f:

To the solution of **7a-f** (0.01g, 0.019 mmol) in DMF (1 mL) was added HATU (0.14g, 0.038 mmol) and DIPEA (50 ul, 0.038 mmol) and then after 5 min, secondary amine **1** (0.01g, 0.017 mmol) in DMF (1 mL) was added and reaction mixture was stirred overnight at room temperature. The solvents were removed under vacuum and residue was purified by silica gel chromatography (10% Methanol in CH_2_Cl_2_ to yield product **8a-f** respectively as a yellow oil.^12^

###### General procedure for synthesis of dCBP-1, dCBP-2, dCBP-3, dCBP-25, dCBP-26 and dCBP-27:

Compound **8a-f** (0.01 mmol) was dissolved in either DCM:TFA (1:1) or 4M-HCl in dioxane and stirred for 30min to 2h. After completion of reaction, solvents were evaporated under reduced pressure and then azeotrope with toluene (2 x 1 mL) followed by drying under vacuum. The crude reaction mixture was dissolved in DCM (1mL); triethylamine (0.1 mmol) and N-methyl-1H-imidazole-1-carboximide **9** (0.03 mmol) was added at rt and stirred overnight. Volatiles were evaporated and the crude reaction mixture was purified by C18 column chromatography using acetonitrile:water (1:1) to yield corresponding compounds **dCBP-1, dCBP-2, dCBP-3, dCBP-25, dCBP-26** and **dCBP-27** respectively as a yellow oil.^12,31^

###### 3-(7-(difluoromethyl)-6-(1-methyl-1H-pyrazol-4-yl)-3,4-dihydroquinolin-1(2H)-yl)-1-(1-(1-((2-(2,6-dioxopiperidin-3-yl)-6-fluoro-1,3-dioxoisoindolin-5-yl)amino)-3,6,9,12-tetraoxapentadecan-15-oyl)piperidin-4-yl)-N-methyl-1,4,6,7-tetrahydro-5H-pyrazolo[4,3-c]pyridine-5-carboxamide (dCBP-25):

^1^H NMR (500 MHz, *CDCl_3_*) δ 8.61 (s, 1H), 7.52 (s, 1H), 7.43 – 7.32 (m, 2H), 7.07 (dd, *J* = 7.1, 2.2 Hz, 1H), 7.02 (s, 1H), 6.82 (s, 1H), 6.52 (t, *J* = 55.6 Hz, 1H), 5.33 (s, 1H), 4.90 (dd, *J* = 12.2, 5.3 Hz, 1H), 4.70 (d, *J* = 13.6 Hz, 1H), 4.57 – 4.37 (m, 1H), 4.17 – 4.06 (m, 1H), 4.02 (d, *J* = 13.9 Hz, 1H), 3.94 (d, *J* = 2.2 Hz, 5H), 3.83 – 3.57 (m, 20H), 3.43 (q, *J* = 5.2 Hz, 2H), 3.14 (t, *J* = 12.7 Hz, 1H), 2.91 – 2.59 (m, 13H), 2.19 – 1.88 (m, 7H). ^13^C NMR (126 MHz, *CDCl_3_*) δ 171.3, 169.3, 168.5, 167.5, 167.0, 167.0, 158.4, 154.9, 152.9, 148.5, 142.9, 142.8, 142.1, 138.9, 137.5, 130.9, 130.1, 130.1, 129.6, 129.5, 129.3, 129.2, 126.4, 121.2, 121.2, 119.5, 118.6, 118.5, 115.6, 113.7, 111.8, 110.3, 110.3, 110.1, 106.0, 105.8, 105.7, 77.2, 70.7, 70.6, 70.6, 70.5, 68.9, 67.5, 55.6, 49.6, 49.4, 44.8, 43.0, 41.4, 40.8, 40.6, 39.2, 33.6, 32.1, 31.6, 27.8, 27.7, 22.8, 22.4, 22.3. HRMS (ESI) calc’d for C_51_H_62_F_3_N_11_O_10_+H = 1046.4706, found 1046.4695.

###### 3-(7-(difluoromethyl)-6-(1-methyl-1H-pyrazol-4-yl)-3,4-dihydroquinoxalin-1(2H)-yl)-1-(1-(3-(2-(2-(2-((2-(2,6-dioxopiperidin-3-yl)-1,3-dioxoisoindolin-5-yl)amino)ethoxy)ethoxy)ethoxy)propanoyl)piperidin-4-yl)-N-methyl-1,4,6,7-tetrahydro-5H-pyrazolo[4,3-c]pyridine-5-carboxamide (dCBP-26):

(23 mg, 48%) as yellow solid. ^1^H NMR (500 MHz, *CD_3_OD*) δ 7.62 (s, 1H), 7.52 (d, *J* = 8.4 Hz, 1H), 7.49 (s, 1H), 7.06 (s, 1H), 6.99 (d, *J* = 2.1 Hz, 1H), 6.83 (dd, *J* = 8.4, 2.2 Hz, 1H), 6.72 (s, 1H), 6.57 (t, *J* = 55.4 Hz, 1H), 5.03 (dd, *J* = 12.5, 5.5 Hz, 1H), 4.64 (d, *J* = 11.0 Hz, 1H), 4.39 – 4.26 (m, 1H), 4.15 – 4.03 (m, 3H), 3.91 (s, 3H), 3.76 – 3.69 (m, 4H), 3.66 – 3.54 (m, 13H), 3.34 (t, *J* = 5.3 Hz, 2H), 3.20 (t, *J* = 12.9 Hz, 1H), 2.95 – 2.73 (m, 9H), 2.68 (s, 4H), 2.62 – 2.55 (m, 1H), 2.13 – 2.00 (m, 4H), 1.95 (dq, *J* = 8.2, 4.0 Hz, 3H). ^13^C NMR (126 MHz, *CD_3_OD*) δ 174.7, 172.0, 171.7, 169.6, 169.2, 160.8, 156.1, 143.8, 140.0, 139.6, 139.0, 135.9, 132.4, 131.7, 131.3, 130.9, 127.3, 126.7, 122.2, 122.2, 122.2, 120.8, 118.3, 108.0, 71.6, 71.5, 71.5, 71.4, 70.3, 68.6, 64.7, 61.4, 56.5, 50.9, 50.7, 49.9, 49.0, 46.2, 44.0, 42.0, 41.7, 38.9, 33.3, 32.6, 28.5, 28.1, 27.3, 23.3, 22.9. HRMS (ESI) calc’d for C_48_H_58_F_2_N_12_O_9_+H = 985.4491, found 985.4539.

###### 3-(7-(difluoromethyl)-6-(1-methyl-1H-pyrazol-4-yl)-3,4-dihydroquinoxalin-1(2H)-yl)-1-(1-(3-(2-(2-((2-(2,6-dioxopiperidin-3-yl)-1,3-dioxoisoindolin-5-yl)amino)ethoxy)ethoxy)propanoyl)piperidin-4-yl)-N-methyl-1,4,6,7-tetrahydro-5H-pyrazolo[4,3-c]pyridine-5-carboxamide (dCBP-27):

(11.4 mg, 56%) as yellow solid. ^1^H NMR (500 MHz, *CD_3_OD*) δ 7.61 (s, 1H), 7.50 (d, *J* = 9.8 Hz, 2H), 7.05 (s, 1H), 6.99 – 6.90 (m, 1H), 6.80 (ddd, *J* = 8.6, 3.9, 2.2 Hz, 1H), 6.73 (s, 1H), 6.56 (t, *J* = 55.4 Hz, 1H), 5.03 (dd, *J* = 12.8, 5.3 Hz, 1H), 4.71 – 4.56 (m, 1H), 4.34 (s, 1H), 4.10 (dd, *J* = 33.3, 10.9 Hz, 3H), 3.91 (s, 3H), 3.84 – 3.56 (m, 14H), 3.23 (dd, *J* = 15.6, 9.5 Hz, 1H), 2.99 – 2.63 (m, 14H), 2.59 – 2.46 (m, 1H), 2.16 – 1.93 (m, 8H). ^13^C NMR (126 MHz, *CD_3_OD*) δ 174.7, 172.1, 171.7, 169.5, 169.2, 160.8, 156.0, 150.1, 143.8, 139.7, 139.5, 135.8, 132.0, 131.1, 130.8, 127.3, 126.2, 122.2, 120.8, 118.2, 116.8, 115.1, 113.2, 111.2, 108.1, 107.1, 71.6, 71.4, 70.4, 68.7, 56.5, 50.9, 50.3, 49.5, 49.5, 49.3, 49.0, 46.3, 44.0, 42.1, 42.0, 41.7, 38.9, 34.5, 33.3, 32.7, 32.2, 28.5, 27.7, 23.8, 23.3, 22.9. HRMS (ESI) calc’d for C_46_H_54_F_2_N_12_O_8_+H = 963.4048, found 963.4092.

##### Synthesis of dCBP-11


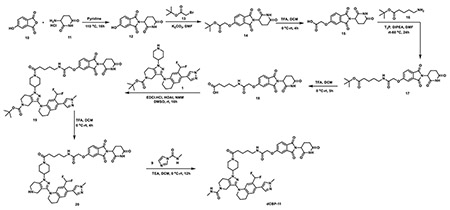


###### Synthesis of 2-(2,6-dioxopiperidin-3-yl)-5-hydroxyisoindoline-1,3-dione (12):

To a stirred solution of 5-hydroxyisobenzofuran-1,3-dione **10** (1.0g, 6.09 mmol) in pyridine was added compound **11** (1.0g, 6.09 mmol) and the reaction mixture was stirred a 110 °C for 16h. After completion of reaction, reaction mixture was concentrated a to afford crude mass (2.5g) as grey solid. It was triturated with diethyl ether (100 mL) and supernatant layer was decanted, and the solid was dried to afford compound **12** (2.0g, 87%) as a tan solid. This was taken for the next step without further any purification.

###### Synthesis of tert-butyl 2-((2-(2,6-dioxopiperidin-3-yl)-1,3-dioxoisoindolin-5-yl)oxy)acetate (14):

To a solution of 2-(2,6-dioxopiperidin-3-yl)-5-hydroxyisoindoline-1,3-dione **12** (1.3g, 3.68 mmol) in DMF (50 mL) was added potassium carbonate (0.76g, 5.52 mmol) followed by addition of tert-butyl 2-bromoacetate **13** (0.72g, 3.68 mmol) and the mixture was stirred at rt for 2h. After completion of reaction mixture was diluted with water (30 mL) and the aqueous layer was extracted with ethyl acetate (3 x 200 mL). The combined organic layers were dried over anhydrous sodium sulphate, filtered, and concentrated to afford crude product (1.5g) as a pale-yellow solid. Crude product was purified by Grace using 24g pre-packed column, and the product was eluted at 75-80% ethyl acetate in hexane to afford desired product **14** (1.1g, 77%) as a yellow solid.

###### Synthesis of 3-(5-(carboxymethoxy)-1,3-dioxoisoindolin-2-yl)-2,6-dioxo piperidin-1-ium-2,2,2-trifluoroacetate (15):

To a solution of compound **14** (1.1g, 2.83 mmol) in TFA (30 mL) was stirred at ambient temperature for 5h. Then the above reaction mixture was concentrated to afford crude product (1.3g) as a yellow solid. It was triturated with hexane (2 x 50 mL), the supernatant layer was decanted, and the solid was dried to afford desired product **15** (1.0g, 76%) as an off-white solid which was used as such for the next step without purification.

###### Synthesis of tert-butyl 5-(2-((2-(2,6-dioxopiperidin-3-yl)-1,3-dioxoisoindolin-5-yl)oxy)acetamido)pentanoate (17):

To a solution of compound **15** (0.32g, 0.717 mmol) in dry DMF (8 mL) was added DIPEA (463mg, 3.59 mmol, d: 0.742 g/mL) followed by addition of T3P (1.27 mL, 2.15 mmol, d:1.08 g/mL, 50% in EtOAc) and tert-butyl 5-aminopentanoate **16** (0.373g, 2.15 mmol). The reaction mixture was stirred at 60 °C for 12h. After completion, reaction mixture was diluted with water (30 mL) and the aqueous layer was extracted with ethyl acetate (4 x 100 mL). Combined organic layers were washed with brine (30 mL), dried over sodium sulphate, filtered, and concentrated to afford crude mass (0.35g) as brown liquid. It was further purified by Grace using pre-packed 24g column and product fraction was eluted at 2-4% methanol in dichloromethane to afford desired product **17** (0.15g, 42%) as off-white solid.

###### Synthesis of 3-(5-(2-((4-carboxybutyl)amino)-2-oxoethoxy)-1,3-dioxoiso indolin-2-yl)-2,6-dioxopiperidin-1-ium-2,2,2-trifluoroacetate (18):

Compound **17** (0.15g, 0.308 mmol) in a mixture of solvents DCM/TFA (1:1; 5mL:5mL) was stirred at ambient temperature for 12h. Completion of reaction was monitored by TLC and after completion, reaction mixture was concentrated to afford crude product (135mg) as brown gum which was purified by preparative HPLC in acetonitrile:water method, the product fraction was concentrated to afford desired product **18** (80mg, 60%) as a white solid.

###### Synthesis of tert-butyl 3-(7-(difluoromethyl)-6-(1-methyl-1H-pyrazol-4-yl)-3,4-dihydroquinolin-1(2H)-yl)-1-(1-(5-(2-((2-(2,6-dioxopiperidin-3-yl)-1,3-dioxoisoindo lin-5-yl)oxy)acetamido)pentanoyl)piperidin-4-yl)-1,4,6,7-tetrahydro-5H-pyrazolo[4,3-c] pyridine-5-carboxylate (19):

To a solution of compound **18** (30mg, 0.07 mmol) in DMSO (4 mL) was added NMM (45mg, 0.439 mmol) followed by addition of EDC.HCl (20mg, 0.102 mmol), HOAt (0.04g, 0.102 mmol) and then intermediate **1** (40mg, 0.058 mmol) was added and the reaction mass was stirred at ambient temperature for 16h. The above reaction mass was diluted with water (15 mL) and extracted with ethyl acetate (3 x 50 mL). The combined organic layers were dried over sodium sulphate, filtered, and concentrated to afford crude product **19** (55mg) as a brown liquid which was used for the next step without purification.

###### Procedure for synthesis 3-(7-(difluoromethyl)-6-(1-methyl-1H-pyrazol-4-yl)-3,4-dihydroquinolin-1(2H)-yl)-1-(1-(5-(2-((2-(2,6-dioxopiperidin-3-yl)-1,3-dioxoisoindolin-5-yl)oxy)acetamido)pentanoyl)piperidin-4-yl)-N-methyl-1,4,6,7-tetrahydro-5H-pyrazolo[4,3-c]pyridine-5-carboxamide (dCBP-11):

To a solution of compound **19** (65mg, 0.039 mmol) in dichloromethane (5 mL), TFA (2 mL) was added, and reaction mixture was stirred at rt for 30 min and after completion of reaction, it was concentrated to remove the excess TFA to afford crude product **20** which was used for the next step as such.

The crude product **20** was dissolved in dichloromethane (5 mL) and added DIPEA (0.40 mL, 0.117 mmol, d: 0.742 g/mL) followed by addition of N-methyl-1H-imidazole-1-carboxamide **9** (8mg, 0.059 mmol) and reaction mixture was stirred at ambient temperature for 16h. After completion of reaction, it was diluted with water (15 mL) and extracted with ethyl acetate (3 x 100 mL). The combined organic layers were dried over sodium sulphate, filtered, and concentrated to afford crude mass (42 mg) as a brown liquid. It was further purified by reverse phase HPLC in ACN/TFA method, and the product fraction was concentrated to afford desired product **dCBP-11** (21mg, 50%) as a brown solid. ^1^H NMR (400 MHz, *CDCl_3_*) δ 8.60 – 8.54 (m, 1H), 7.81 (d, *J* = 8.3 Hz, 1H), 7.59 (s, 1H), 7.48 – 7.32 (m, 3H), 7.30 – 7.23 (m, 2H), 7.03 (s, 1H), 6.83 (s, 1H), 6.51 (t, *J* = 55.5 Hz, 1H), 4.95 (dd, *J* = 12.1, 5.4 Hz, 6H), 3.98 (d, *J* = 4.6 Hz, 6H), 3.83 – 3.60 (m, 5H), 3.37 (q, *J* = 6.2 Hz, 2H), 2.92 – 2.82 (m, 3H), 2.77 (d, *J* = 11.3 Hz, 7H), 2.41 (t, *J* = 6.7 Hz, 2H), 2.22 – 2.10 (m, 2H), 2.08 – 1.97 (m, 5H), 1.72 – 1.60 (m, 4H). ^13^C NMR (101 MHz, *CDCl_3_*) δ 171.7, 171.2, 168.3, 167.4, 166.8, 162.7, 158.8, 148.6, 142.3, 138.4, 137.8, 134.5, 131.2, 129.8, 129.7, 126.6, 125.9, 124.9, 120.9, 120.4, 120.3, 119.7, 116.1, 113.7, 111.4, 110.6, 110.4, 110.4, 106.0, 106.0, 77.2, 72.3, 67.8, 67.7, 61.9, 55.7, 55.6, 49.7, 49.5, 44.7, 41.5, 41.2, 40.9, 39.1, 39.0, 32.3, 32.0, 31.6, 31.6, 28.6, 28.6, 27.9, 27.6, 22.8, 22.4, 21.9. HRMS (ESI) calc’d for C_47_H_53_F_2_N_11_O_8_+H = 938.4119, found 938.4135.

##### Synthesis of dCBP-12


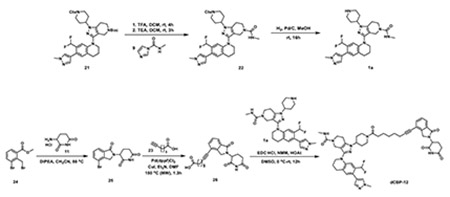


###### Synthesis of 3-(7-(difluoromethyl)-6-(1-methyl-1H-pyrazol-4-yl)-3,4-dihydroquinolin-1(2H)-yl)-N-methyl-1-(piperidin-4-yl)-1,4,6,7-tetrahydro-5H-pyrazolo[4,3-c]pyridine-5-carboxamide (1a):

To a solution of compound **21**^30^ (0.52g, 0.741 mmol) in dichloromethane (20 mL), trifluoro acetic acid (0.171 mL, 2.22 mmol, d: 1.48g/mL) was added and the reaction mixture was stirred at ambient temperature for 4h. The reaction mass was concentrated to afford crude product (0.53g) as a brown gum, which was used for the next step without any purification. LC-MS: m/z 602.80 {(M+1)-TFA}.

The crude product (0.24g, 0.259 mmol) in dichloromethane (10 mL), triethyl amine (0.181 mL, 1.297 mmol, d: 0.726 g/ml) was added followed by addition of *N*-methyl-1H-imidazole-1-carboxamide **9** (49 mg, 0.389 mmol) and the reaction mixture was stirred at ambient temperature for 3h. The reaction mass was diluted with water (30 mL) and extracted with ethyl acetate (3x75 mL). The combined organic layers were dried over anhydrous sodium sulphate, filtered and concentrated to afford crude mass (0.171g) as a yellow liquid. It was purified by C18 column using CAN:water as solvent which afforded desired intermediate **22** (0.11g, 55%) as an off-white solid. LC-MS: m/z 659.30 (M+1).

To a solution of intermediate **22** (0.46g, 0.698 mmol) in methanol (25 mL) was added Pd/C (10% on carbon) (0.45 g, 0.419 mmol, 100% wt/wt) and reaction mixture was stirred under hydrogen atmosphere in a balloon pressure at ambient temperature for 16h. The reaction mass was passed through the celite bed, and it was washed with methanol (75 mL), the combined filtrates were concentrated to afford **1a** (0.32 g, 71%) as an off-white solid, which was used for the next step without any purification. LC-MS: m/z 525.70 (M+1).

###### Synthesis of 3-(4-bromo-1-oxoisoindolin-2-yl)piperidine-2,6-dione (25):

To a solution of compound **24** (2.5g, 8.12 mmol) in acetonitrile (50 mL) was added DIPEA (7.1 mL, 40.6 mmol, d: 0.74 g/mL) followed by addition of 3-aminopiperidine-2,6-dione hydrochloride **11** (2g, 12.2 mmol) and the reaction mass was stirred at 80 °C. After completion of reaction, it was concentrated, and the residue was triturated with methyl tert-butyl ether (2 x 200 mL). The supernatant layer was decanted and dried to afford crude product **25** (2.2g, 83%) as a pale purple solid. LC-MS: m/z 323.90 (M+1).

###### Synthesis of 8-(2-(2,6-dioxopiperidin-3-yl)-1-oxoisoindolin-4-yl)oct-7-ynoic acid (26):

To a solution of compound **25** (0.2g, 0.619 mmol) in DMF (10 mL) solid CuI (28mg, 0.149 mmol) was added following the addition of Pd(dppf)Cl2 (36 mg, 0.05 mmol), TEA (0.43 mL, 3.09 mmol, d: 0.724 g/mL) and oct-7-ynoic acid **23** (0.13g, 0.928 mmol) and the mixture was stirred at 80 °C in MW for 1.3h. After completion of reaction, it was diluted with water (25 mL) and 1.5N aq. HCl solution (10 mL). Further it was extracted with ethyl acetate (3 x 100 mL) and the combined organic layers were dried over sodium sulphate, filtered, and concentrated to afford crude (0.31g) as a brown liquid. It was further purified by reverse phase HPLC purification and desired fraction was lyophilized to afford product **26** (80mg, 25%) as an off-white solid. LC-MS: m/z 383.10 {(M+1)-TFA}.

###### Synthesis of 3-(7-(difluoromethyl)-6-(1-methyl-1H-pyrazol-4-yl)-3,4-dihydroquinolin-1(2H)-yl)-1-(1-(8-(2-(2,6-dioxopiperidin- 3-yl)-1-oxoisoindolin -4-yl)oct-7-ynoyl)piperidin-4-yl)-N-methyl-1,4,6,7-tetrahydro-5H-pyrazolo[4,3-c] pyridine-5-carboxamide (dCBP-12):

To a solution of amine intermediate **1a** (25mg, 0.039 mmol) in DMSO (2 mL) was added *N*-methyl morpholine (NMM) (28mg, 0.274 mmol), HOAt (10 mg, 0.069 mmol), EDC.HCl (14mg, 0.069 mmol) followed by addition of compound **26** (24 mg, 0.047 mmol). The reaction mixture was stirred at ambient temperature for 12h and after completion, it was diluted with 10% aq. NaHCO3 solution (20 mL). Aq. layer was extracted with ethyl acetate (3 x 25 mL), dried over sodium sulphate, filtered, and concentrated to afford crude product (36mg) as pink gum. This crude was purified by reverse phase preparative HPLC in ACN/TFA method. Product fraction was concentrated, and the residue was diluted with 10% aqueous sodium bicarbonate solution (10 mL), extracted with ethyl acetate (3 x 20 mL), dried over sodium sulphate, filtered, and concentrated to afford **dCBP-12** (15mg, 42%) as a yellow solid. LC-MS: m/z 890.06 (M+1).

###### 3-(7-(difluoromethyl)-6-(1-methyl-1H-pyrazol-4-yl)-3,4-dihydroquinolin-1(2H)-yl)-1-(1-(8-(2-(2,6-dioxopiperidin-3-yl)-1-ox-oisoindolin-4-yl)oct-7-ynoyl)piperidin-4-yl)-N-methyl-1,4,6,7-tetrahydro-5H-pyrazolo[4,3-c]pyridine-5-carboxamide (dCBP-12):

^1^H NMR (400 MHz, *CDCl_3_*) δ 8.53 – 8.30 (m, 1H), 7.78 (dd, *J* = 7.6, 1.1 Hz, 1H), 7.56 (dd, *J* = 7.6, 1.1 Hz, 2H), 7.42 (t, *J* = 7.6 Hz, 2H), 7.03 (s, 1H), 6.83 (s, 1H), 6.53 (t, *J* = 55.6 Hz, 1H), 5.20 (dt, *J* = 12.9, 6.3 Hz, 1H), 4.50 (d, *J* = 16.7 Hz, 1H), 4.36 (dd, *J* = 16.9, 2.4 Hz, 1H), 4.13 (td, *J* = 10.9, 5.4 Hz, 1H), 3.96 (d, *J* = 2.7 Hz, 5H), 3.86 – 3.59 (m, 4H), 2.98 – 2.65 (m, 10H), 2.55 – 2.28 (m, 11H), 2.10 – 1.92 (m, 5H), 1.76 – 1.60 (m, 4H), 1.53 (h, *J* = 8.0, 6.8 Hz, 2H). ^13^C NMR (101 MHz, *CDCl_3_*) δ 171.5, 171.3, 169.7, 169.7, 169.3, 158.5, 148.6, 143.8, 142.2, 137.6, 134.7, 131.6, 131.0, 129.6, 128.5, 126.5, 123.3, 121.2, 119.7, 116.1, 113.7, 111.4, 110.4, 106.2, 106.1, 96.2, 77.2, 55.7, 55.6, 52.0, 52.0, 49.7, 47.2, 47.2, 44.8, 41.4, 40.9, 40.7, 39.2, 33.3, 32.2, 31.7, 29.8, 28.8, 28.7, 28.5, 28.5, 27.8, 27.7, 25.0, 24.9, 23.5, 22.4, 22.4, 19.5, 19.4. HRMS (ESI) calc’d for C_48_H_54_F_2_N_10_O_5_+H = 889.4319, found 889.4315.

##### Synthesis of dCBP-13


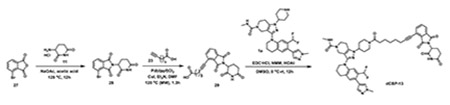


###### Synthesis of 4-bromo-2-(2,6-dioxopiperidin-3-yl)isoindoline-1,3-dione (28):

To a solution of compound **27** (3g, 13.2 mmol) in acetic acid (40 mL) was added sodium acetate and 3- aminopiperidine-2,6-dione hydrochloride **11** (2.83g, 17.2 mmol) sequentially and reaction mixture was stirred at 120 °C for 12h. After completion of reaction, it was concentrated under reduced pressure to remove most acetic acid and residue was poured into water (100 mL) and stirred for 10 minutes then it was filtered. The filtered cake was washed with cold water (2 x 30 mL) and dried to give crude mass (4.5g) as an off-white solid. The above crude was triturated with cold water (3 x 150 mL) and methyl tert-butyl ether (3 x 100 mL). The solid was dried to afford dried to afford desired product **28** (3.8g, 86%) as an off-white solid.

###### Synthesis of 8-(2-(2,6-dioxopiperidin-3-yl)-1,3-dioxoisoindolin-4-yl)oct-7-ynoic acid (29):

To a stirred solution of compound **28** (200mg, 0.593 mmol) in DMF (5 mL) was added CuI (34mg, 0.178 mmol), Pd(dppf)Cl_2_ (43mg, 0.059 mmol), TEA (300mg, 2.97 mmol, d: 0.724 gm/mL) followed by addition of **23** (125mg, 0.89 mmol). The reaction mixture was processed in microwave at 120 °C for 1.3h. After completion, the reaction mixture was cooled at rt and it was passed twice through a celite bed and washed with methanol, the filtrate was concentrated. Now, this residue was diluted with water (25 mL) and 1.5N aq. HCl solution (10 mL). Aqueous layer was extracted with ethyl acetate (3 x 100 mL). Combined organic layers were dried over sodium sulphate, filtered, and concentrated to afford crude product (305 mg) as a brown liquid. After reverse phase purification (C18), using acetonitrile:water (1:1) the product fraction was concentrated to afford desired compound **29** (123mg, 43%) as an off-white solid.

###### Synthesis of 3-(7-(difluoromethyl)-6-(1-methyl-1H-pyrazol-4-yl)-3,4-dihydroquinolin-1(2H)-yl)-1-(1-(8-(2-(2,6-dioxopiperidin-3-yl)-1,3-dioxoisoindolin-4-yl)oct-7-ynoyl)piperidin-4-yl)-N-methyl-1,4,6,7-tetrahydro-5H-pyrazolo[4,3-c]pyridine-5-carboxamide (dCBP-13):

To a stirred solution of intermediate **1a** (25mg, 0.039 mmol) in DMSO (2 mL) was added NMM (28 mg, 0.274 mmol), HOAt (10mg, 0.069 mmol), EDC.HCl (15mg, 0.069 mmol) followed by addition of acid **29** (24mg, 0.047 mmol) and the reaction mixture was stirred at ambient temperature for 12h. The reaction mass was diluted with 10% aq. NaHCO_3_ solution (20 mL), it was extracted with ethyl acetate (3 x 25 mL), dried over sodium sulphate, filtered, and concentrated to afford crude mass (40mg) as a pink gum which was purified over reverse phase HPLC using acetonitrile:water (1:1). The product fraction was concentrated and diluted with 10% aqueous NaHCO_3_ solution (10 mL), it was extracted with ethyl acetate (3 x 20 mL), dried over sodium sulphate, filtered, and concentrated to afford desired product **dCBP-13** (22mg, 59%) as a yellow solid. LC-MS: m/z 904.00 (M+1). ^1^H NMR (400 MHz, *CDCl_3_*) δ 8.63 – 8.57 (m, 1H), 7.75 (dd, *J* = 7.2, 1.2 Hz, 1H), 7.71 – 7.58 (m, 2H), 7.53 (s, 1H), 7.41 (s, 1H), 7.03 (s, 1H), 6.83 (d, *J* = 3.0 Hz, 1H), 6.53 (t, *J* = 55.6 Hz, 1H), 4.94 (dt, *J* = 11.7, 5.3 Hz, 1H), 4.73 (d, *J* = 13.5 Hz, 1H), 4.43 (d, *J* = 5.3 Hz, 1H), 4.12 (ddt, *J* = 10.7, 7.9, 4.0 Hz, 1H), 3.95 (d, *J* = 3.5 Hz, 6H), 3.84 – 3.61 (m, 4H), 2.92 – 2.66 (m, 11H), 2.53 (t, *J* = 6.8 Hz, 2H), 2.39 (t, *J* = 7.6 Hz, 2H), 2.16 – 1.91 (m, 7H), 1.75 – 1.52 (m, 7H). ^13^C NMR (101 MHz, *CDCl_3_*) δ 171.6, 171.1, 168.2, 166.6, 166.2, 158.4, 148.6, 142.2, 139.0, 138.6, 137.6, 134.0, 132.3, 131.0, 130.7, 129.2, 126.5, 122.6, 121.9, 119.5, 113.7, 110.4, 106.2, 99.6, 77.2, 76.3, 55.8, 49.7, 49.4, 44.8, 41.4, 41.4, 40.8, 40.6, 39.2, 33.4, 32.2, 31.7, 31.5, 29.8, 28.7, 28.2, 27.8, 27.7, 25.0, 22.7, 22.4, 22.3, 19.8. HRMS (ESI) calc’d for C_48_H_52_F_2_N_10_O_6_+H = 903.4112, found 903.4114.

##### Synthesis of dCBP-14


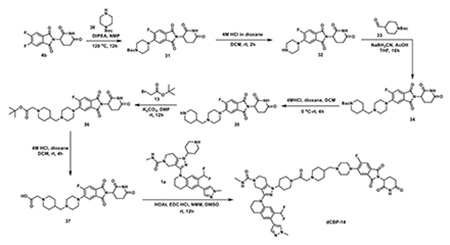


###### Synthesis of tert-butyl 4-(2-(2,6-dioxopiperidin-3-yl)-6-fluoro-1,3-dioxoisoindolin-5-yl)piperazine-1-carboxylate (31):

To a stirred solution of compound **4b** (1g, 3.4 mmol) in NMP (25 mL) was added DIPEA (1.8 mL, 10.2 mmol, d: 0.742 g/mL) followed by addition of amine **30** (0.66g, 3.57 mmol) and the reaction mixture was stirred at 120 °C for 12h. The above reaction mass was diluted with water and extracted with ethyl acetate. The combined organic layers were dried over sodium sulphate, filtered and concentrated to afford crude mass (1.56g) as a brown liquid. It was purified by Isolera, the product fraction was eluted using 30-35% ethyl acetate in hexane to afford desired product **31** (0.8g, 51%) as an off-white solid. LC-MS: m/z 361.1 {(M+1-Boc}.

###### Synthesis of 2-(2,6-dioxopiperidin-3-yl)-5-fluoro-6-(piperazin-1-yl)isoindoline-1,3-dione (32):

To a stirred solution of **31** (0.8g, 1.74 mmol) in DCM (15 mL) was added 4M HCl in dioxane (2.1 mL, 8.69 mmol) and the reaction mass was stirred at ambient temperature for 2h. The above reaction mass was concentrated to afford crude mass (0.69g) as a yellow solid. It was triturated with methyl tert-butyl ether (2 x 50 mL), the supernatant layer was decanted and concentrated to afford desired product **32** (0.6g, 87%) as a yellow solid and used as such for the next step. LC-MS: m/z 361.20 {(M+1-HCl}.

###### Synthesis of tert-butyl 4-((4-(2-(2,6-dioxopiperidin-3-yl)-6-fluoro-1,3-dioxoisoindolin-5-yl)piperazin-1-yl)methyl)piperidine-1-carboxylate (34):

To a stirred solution of **32** (0.45g, 1.25 mmol) in THF (25 mL) was added aldehyde **33** (0.35g, 1.65 mmol) followed by catalytic amount of acetic acid (15mg, 0.25 mmol) followed by sodium cyanoborohydride (0.25g, 4 mmol) and the mixture was stirred at ambient temperature for 16h. The above reaction mass was diluted with water and extracted with ethyl acetate. The combined organic layers were dried over sodium sulphate, filtered, and concentrated to afford crude mass (0.7g) as a yellow liquid. It was purified by Isolera (24g cartridge, 60-120 silica gel), the product fraction was eluted using 3-4% methanol in DCM to afford desired product **34** (0.5g, 72%) as a yellow solid. LC-MS: m/z 558.65 (M+1).

###### Synthesis of tert-butyl 2-(4-((4-(2-(2,6-dioxopiperidin-3-yl)-6-fluoro-1,3-dioxoisoindolin-5-yl)piperazin-1-yl)methyl)piperidin-1-yl)acetate (36):

To a stirred solution of compound **34** (0.5g, 0.897 mmol) in DCM (20 mL) was added 4M HCl in dioxane (1.12 ml, 4.48 mmol) and the reaction mass was stirred at ambient temperature for 4h. The above reaction mass was concentrated to afford crude mass (0.45g) as a yellow solid. The above solid was triturated with methyl tert-butyl ether and then dried to afford desired product **35** (400mg, 87%) as a yellow solid. LC-MS: m/z 458.51 {(M+1)-HCl}.

To a stirred solution of compound **35** (0.3g, 0.61 mmol) in DMF (10 mL) was added potassium carbonate (0.34g, 2.43 mmol) followed by addition of tert-butyl 2-bromoacetate **13** (0.13g, 0.668 mmol) and reaction mass was stirred at ambient temperature for 12h. The above reaction mass was diluted with water (30 mL) and extracted with ethyl acetate (3 x 100 mL). The combined organic layers were dried over sodium sulphate, filtered, and concentrated to afford crude mass (0.35g) as yellow liquid. Crude product was purified by Isolera and product fraction was eluted at 3-4% methanol in dichloromethane to afford desired product **36** (0.15g, 38%) as a yellow solid. LC-MS: m/z 572.10 (M+1).

###### Procedure for synthesis of dCBP-14:

To a stirred solution of compound **36** (140mg, 0.245 mmol) in dichloromethane (10 mL) was added 4M HCl in dioxane (0.61 mL, 2.45 mmol) and the reaction mass was stirred at an ambient temperature for 4h. The above reaction mass was concentrated to afford desired crude product **37** (0.12g) as a yellow solid and used for the next step without further purification. LC-MS: m/z 516.20 {(M+1)-HCl}.

To a stirred solution of compound **37** (30mg, 0.054 mmol) in DMSO (5 mL) was added NMM (33mg, 0.326 mmol) followed by addition of HOAt (15mg, 0.109 mmol), EDC.HCl (21mg, 0.109 mmol) and addition of **1a** (29mg, 0.054 mmol). Reaction mixture was stirred at rt for 12h for completion and then reaction mass was diluted with water (15 mL) and extracted with ethyl acetate (3 x 50 mL). The combined organic layers were dried over sodium sulphate, filtered, and concentrated to afford crude mass (56mg) as a yellow liquid. It was purified using C18 column and desired product fraction was concentrated to afford product as a yellow gum. Product was diluted with 10% aqueous sodium bicarbonate solution (20 mL), extracted with ethyl acetate (3 x 50 mL), combined extracts were dried over sodium sulphate, filtered and concentrated to afford pure **dCBP-14** (28mg, 48%) as a yellow solid. ^1^H NMR (400 MHz, *CD_3_OD*) δ 7.62 (s, 1H), 7.55 – 7.42 (m, 3H), 7.08 (s, 1H), 6.74 (s, 1H), 6.57 (t, *J* = 55.5 Hz, 1H), 5.08 (dd, *J* = 12.5, 5.5 Hz, 1H), 4.63 (d, *J* = 13.4 Hz, 1H), 4.39 (dd, *J* = 12.9, 7.4 Hz, 1H), 4.09 (s, 3H), 3.92 (s, 4H), 3.76 – 3.57 (m, 6H), 3.29 (d, *J* = 5.0 Hz, 6H), 2.86 (ddd, *J* = 20.4, 6.6, 3.8 Hz, 7H), 2.77 – 2.52 (m, 13H), 2.31 (d, *J* = 7.0 Hz, 2H), 2.17 – 1.88 (m, 10H), 1.55 – 1.40 (m, 2H). ^13^C NMR (101 MHz, *CD_3_OD*) δ 174.5, 171.4, 168.4, 167.9, 167.9, 163.2, 162.8, 160.8, 160.8, 158.3, 150.2, 147.1, 147.0, 143.9, 139.9, 139.5, 132.1, 131.2, 131.0, 131.0, 130.8, 130.5, 130.5, 127.3, 125.6, 125.5, 122.3, 120.9, 119.7, 117.4, 116.8, 115.0, 114.6, 114.5, 112.8, 112.7, 112.5, 111.3, 111.2, 108.2, 64.8, 59.7, 56.2, 55.0, 54.8, 54.5, 50.9, 50.9, 50.7, 49.0, 45.4, 42.4, 42.0, 41.7, 38.9, 33.1, 32.9, 32.5, 32.2, 30.7, 30.1, 28.5, 27.7, 23.7, 23.3, 22.9. HRMS (ESI) calc’d for C_52_H_62_F_3_N_13_O_6_+H = 1022.4971, found 1022.4966.

##### Synthesis of dCBP-15


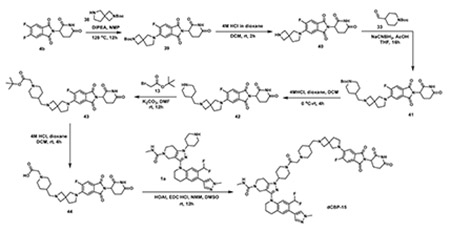


###### Synthesis of 39:

To a solution of compound **4b** (1.0g, 3.4 mmol) in dry NMP (20 mL), triethyl amine (1.03 g, 10.2 mmol, d: 0.724 g/mL) was added followed by addition of **38** (0.79g, 3.74 mmol) and the whole mixture was stirred at 120 °C for 12h. The reaction mass was diluted with water (40 mL) and it was extracted with ethyl acetate (3 x 200 mL). The combined organic layers were dried over anhydrous sodium sulphate, filtered and concentrated to afford crude mass as a yellow liquid which was further purified using silica gel column chromatography using MeOH:DCM (1:9) to afford compound **39** (1.0g, 60%) as a yellow solid. LC-MS: m/z 487.10 (M+1).

###### Synthesis of 40:

To a solution of compound **39** (1.0g, 2.06 mmol) in DCM (20 mL), 4M-HCl in dioxane (2.57 mL, 10.28 mmol) was added and reaction mixture was stirred at ambient temperature for 2h. The reaction mass was concentrated and dried to afford crude mass (0.87g) as a yellow solid. The crude product was triturated with methyl tert-butyl ether (50 mL) and super natant layer was decanted to afford the solid which was dried to afford desired product **40** (0.8g, 88%) as a yellow solid. LC-MS: m/z 387.20 {(M+1)-HCl}.

###### Synthesis of 41:

To a solution of compound **40** (0.3g, 0.776 mmol) and aldehyde **33** (0.22g, 1.01 mmol) in a mixture of tetrahydrofuran/methanol (4:1) (10 mL), catalytic amount of acetic acid (0.01g, 0.155 mmol) was added followed by addition of sodium cyanoborohydride (0.146g, 2.33 mmol) and the reaction mixture was stirred at an ambient temperature for 16h. The reaction mass was diluted with water (30 mL), and it was extracted with ethyl acetate (3 x 100 mL). The combined organic layers were dried over anhydrous sodium sulphate, filtered, and concentrated to afford crude mass (0.46 g) as a yellow liquid. It was purified by Isolera, the product fraction was eluted at 2-3% methanol in dichloromethane to afford compound **41** (0.25g, 29%) as a yellow gum. LC-MS: m/z 584.10 (M+1).

###### Synthesis of 42:

To a solution of compound **41** (0.25g, 0.428 mmol) in dichloromethane (5 mL), 4M HCl in dioxane (0.13 mL) was added and reaction mass was stirred at an ambient temperature for 3h. The reaction mass was concentrated to afford crude product **42** (0.2g, 69%) as a yellow solid, which was used for next step without any purification. LC-MS: m/z 484.15 {(M+1)-HCl}.

###### Synthesis of 43:

To a solution of compound **42** (0.19g, 0.318 mmol) in tetrahydrofuran (10 mL), DIPEA (0.15 mL, 0.954 mmol, d: 0.742 g/mL) was added followed by addition of tert-butyl 2-bromo acetate **13** (0.08g, 0.413 mmol) and reaction mass was stirred at an ambient temperature for 12h. The reaction mass was diluted with water (30 mL) and extracted with ethyl acetate (3 x 100 mL). The combined organic layers were dried over anhydrous sodium sulphate, filtered, and concentrated to afford crude mass (0.2g) as a yellow liquid. It was purified by preparative HPLC in Acetonitrile:water (1:1) and desired fraction was concentrated to afford product **43** (0.1g, 44%) as a yellow solid. LC-MS: m/z 598.20 {(M+1)-TFA}.

###### Synthesis of 44:

To a solution of **43** (0.3g, 0.502 mmol) in dichloromethane (10 mL) was added trifluoro acetic acid (0.2 mL, 2.51 mmol, 1.48 g/mL) and the reaction mass was stirred at an ambient temperature for 2h. The reaction mass was concentrated to afford crude mass (0.28g) as a yellow liquid. It was purified by preparative HPLC in Acetonitrile:water (1:1) and desired fraction was concentrated to afford product **44** (25 mg, 9%) as yellow gum. LC-MS: m/z 542.20 {(M+1)-TFA}.

###### Synthesis of 3-(7-(difluoromethyl)-6-(1-methyl-1H-pyrazol-4-yl)-3,4-dihydroquinolin-1(2H)-yl)-1-(1-(2-(4-((6-(2-(2,6-dioxopiperidin-3-yl)-6-fluoro-1,3-dioxoisoindolin-5-yl)-2,6-diazaspiro[3.4]octan-2-yl)methyl)piperidin-1-yl)acetyl)piperidin-4-yl)-N-methyl-1,4,6,7-tetrahydro-5H-pyrazolo[4,3-c]pyridine-5-carboxamide (dCBP-15):

To a solution **44** (25mg, 0.038 mmol) and amine **1a** (25mg, 0.046 mmol) in dimethyl sulfoxide (2 mL), 1-hydroxy-7-azabenzotriazole (11mg, 0.076 mmol) and 1-Ethyl-3-(3-dimethylaminopropyl) carbodiimide (15 mg, 0.076 mmol) were added sequentially followed by addition of N-Methyl morpholine (27mg, 0.267 mmol). Reaction mixture was stirred at an ambient temperature for 12h. The reaction mass was diluted with water (5 mL), and it was extracted with ethyl acetate (3 x 50 mL). The combined organic layers were dried over sodium sulphate, filtered, and concentrated to afford crude mass (40mg) as a brown liquid. It was purified by preparative HPLC by Acetonitrile:water (1:1, 0.1% TFA) and desired fraction was concentrated and diluted with 10% aqueous sodium bicarbonate solution (25 mL), extracted with ethyl acetate (3 x 25 mL), combined extracts were dried over anhydrous sodium sulphate, filtered and concentrated to afford pure **dCBP-15** (18mg, 44%) as a yellow solid. LC-MS: m/z 1049.20 (M+1). ^1^H NMR (401 MHz, *CD_3_OD*) δ 7.62 (s, 1H), 7.51 – 7.43 (m, 2H), 7.13 (d, *J* = 7.5 Hz, 1H), 7.08 (s, 1H), 6.72 (s, 1H), 6.57 (t, *J* = 55.5 Hz, 1H), 5.06 (dd, *J* = 12.6, 5.5 Hz, 1H), 4.68 – 4.59 (m, 1H), 4.48 – 4.20 (m, 7H), 4.09 (s, 2H), 3.92 (s, 7H), 3.69 (dt, *J* = 30.1, 5.7 Hz, 9H), 3.12 (d, *J* = 15.0 Hz, 2H), 2.99 – 2.80 (m, 7H), 2.76 – 2.65 (m, 6H), 2.38 (s, 2H), 2.20 – 1.64 (m, 13H). ^13^C NMR (101 MHz, *CD_3_OD*) δ 174.6, 171.6, 168.6, 168.2, 163.5, 162.2, 161.8, 160.8, 156.8, 154.3, 150.3, 143.9, 143.1, 140.0, 139.5, 132.1, 131.2, 131.1, 127.3, 122.4, 120.9, 119.2, 117.4, 115.0, 112.8, 112.7, 112.6, 111.2, 110.7, 108.4, 64.5, 60.9, 59.4, 59.4, 58.1, 55.9, 54.4, 50.9, 50.6, 49.0, 44.8, 42.4, 42.0, 41.7, 41.5, 38.9, 35.6, 32.8, 32.3, 32.2, 31.3, 30.7, 28.5, 28.0, 27.7, 23.8, 23.3, 22.9. HRMS (ESI) calc’d for C_54_H_64_F_3_N_13_O_6_+H = 10485127, found 1048.5126.

##### Synthesis of dCBP-28


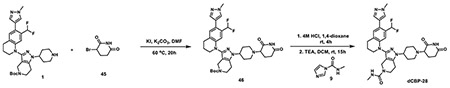


###### Procedure:

To a solution of amine compound **1** (30mg, 0.053 mmol) in dry DMF (1.0mL), bromo compound **45** (20mg, 0.106mmol) was added following the addition of KI (9mg, 0.053 mmol) and K_2_CO_3_ (15mg, 0.106 mmol) at rt and then reaction mixture was heated at 60 °C for 20h. After completion of reaction as monitored by LC-MS, it was cooled at rt. To this reaction mixture, water was added, and aq. layer was extracted with EtOAc (3 times). Combined organic layers were dried over Na_2_SO_4_ and concentrated to get the crude product which was purified using silica-gel column by MeOH:DCM (2:8) as eluent to get the desired product **46** (29mg, 80%) as brown gum. LC-MS: m/z 679.0 (M+1).

Intermediate **46** (29mg, 0.043) was dissolved 1.0 mL 4M-HCl in dioxane solution and stirred at rt for 4h. After completion of reaction, solvents were evaporated under reduced pressure and then azeotrope with toluene (2 x 1 mL) followed by drying under vacuum. The crude reaction mixture was dissolved in DCM (1.5mL); triethylamine (60 μL, 0.427 mmol) and N-methyl-1H-imidazole-1-carboximide **9** (16mg, 0.128 mmol) was added at rt and stirred overnight. After completion of reaction, volatiles were evaporated, and crude reaction mixture was purified by C18 column chromatography using acetonitrile water (1:1) to yield corresponding compounds **dCBP-28** as white solid (13 mg, 48%). ^1^H NMR (500 MHz, *CDCl_3_*) δ 7.95 (d, *J* = 3.9 Hz, 1H), 7.53 (s, 1H), 7.40 (s, 1H), 7.03 (s, 1H), 6.84 (s, 1H), 6.53 (t, *J* = 55.6 Hz, 1H), 4.43 (q, *J* = 4.6 Hz, 1H), 3.95 (s, 6H), 3.77 (t, *J* = 5.7 Hz, 2H), 3.73 – 3.67 (m, 2H), 3.39 (dd, *J* = 10.6, 4.5 Hz, 1H), 3.06 – 2.90 (m, 3H), 2.85 (t, *J* = 6.5 Hz, 2H), 2.75 (dd, *J* = 15.2, 5.2 Hz, 5H), 2.70 – 2.64 (m, 1H), 2.62 – 2.52 (m, 1H), 2.30 – 2.16 (m, 2H), 2.14 – 1.88 (m, 7H). ^13^C NMR (126 MHz, *CDCl_3_*) δ 172.2, 172.1, 158.4, 148.3, 142.2, 139.0, 137.4, 130.9, 129.6, 129.5, 129.3, 129.2, 126.4, 126.3, 121.1, 121.1, 121.0, 119.5, 115.6, 113.8, 111.9, 110.4, 110.3, 110.3, 105.8, 77.2, 64.1, 56.2, 51.0, 49.6, 47.2, 41.4, 40.6, 39.2, 32.5, 32.1, 30.9, 27.8, 27.7, 22.5, 22.4, 22.3. HRMS (ESI) calc’d for C_32_H_39_F_2_N_9_O_13_+H = 636.3217, found 636.3218.

##### Synthesis of dCBP-29, dCBP-30 and dCBP-30-Bump


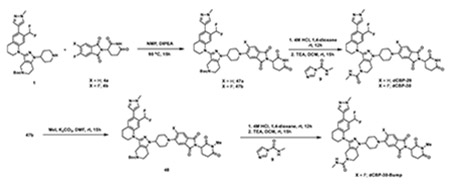


###### Procedure for synthesis of dCBP-29:

To a solution of amine compound **1**, (70mg, 0.123 mmol) in dry DMP (1.5 mL), compound **4a** (68mg, 0.247 mmol) was added following the addition of DIPEA (86 μL, 0.493 mmol) and reaction mixture was heated at 90 °C for 15h. After completion of reaction, it was cooled to rt, and water was added. The aq. layer was extracted using EtOAc (3 x 5 mL). All the organic layers were collectively dried over Na_2_SO_4_ and then concentrated. The crude reaction mixture was purified over silica gel column chromatography using MeOH:DCM (2:8) as eluent to get the product **47a** as light-yellow gum (66mg, 65%). LC-MS: m/z 824.0 (M+1).

Compound **47a** (43 mg, 0.052 mmol) was dissolved in 4M HCl in 1,4-dioxane (1.5 mL) and stirred for 12h. After completion of reaction, solvent was evaporated, and crude product was azeotrope using toluene (3x1 mL) and resulted crude product was used as such for the next step. LC-MS: m/z 724.0 (M+1). The crude product (37 mg, 0.051) was dissolved in DCM (1.5mL); triethylamine (71micro L, 0.511 mmol) and N-methyl-1H-imidazole-1-carboximide **9** (12.8 mg, 0.102 mmol) at rt and reaction mixture was stirred overnight. After completion of reaction, volatiles were evaporated, and the crude reaction mixture was purified by C18 column chromatography using acetonitrile water (1:1) to yield corresponding compounds **dCBP-29** as yellow solid (16.5 mg, 41%). ^1^H NMR (400 MHz, *CD_3_OD*) δ 7.68 (d, *J* = 8.5 Hz, 1H), 7.62 (d, *J* = 0.8 Hz, 1H), 7.49 (d, *J* = 0.8 Hz, 1H), 7.40 (d, *J* = 2.3 Hz, 1H), 7.29 – 7.22 (m, 1H), 7.07 (t, *J* = 1.1 Hz, 1H), 6.72 (s, 1H), 6.56 (t, *J* = 55.5 Hz, 1H), 5.06 (dd, *J* = 12.5, 5.4 Hz, 1H), 4.47 – 4.28 (m, 1H), 4.20 (d, *J* = 13.4 Hz, 2H), 4.08 (s, 2H), 3.92 (s, 3H), 3.73 (t, *J* = 5.7 Hz, 2H), 3.66 – 3.57 (m, 2H), 3.26 – 3.05 (m, 2H), 2.92 – 2.79 (m, 5H), 2.76 – 2.55 (m, 5H), 2.22 (qd, *J* = 12.3, 3.9 Hz, 2H), 2.13 – 1.89 (m, 6H). ^13^C NMR (101 MHz, *CD_3_OD*) δ 174.7, 171.6, 169.4, 169.0, 160.8, 156.5, 150.2, 143.8, 139.8, 139.5, 135.8, 132.1, 131.2, 131.1, 131.0, 130.8, 127.3, 126.2, 122.3, 122.2, 122.2, 120.9, 120.2, 119.4, 117.4, 115.0, 112.7, 111.2, 111.2, 109.5, 108.2, 56.8, 50.9, 50.4, 49.0, 48.2, 42.0, 41.7, 38.9, 32.2, 32.1, 28.5, 27.7, 23.8, 23.3, 23.0. HRMS (ESI) calc’d for C_40_H_42_F_2_N_10_O_5_+H = 781.3380, found 781.3377.

###### Procedure for synthesis of compound 47b:

To a solution of amine compound **1** (80 mg, 0.141 mmol) in dry NMP (1.5 mL), compound **4b** (83mg, 0.282 mmol) was added following the addition of DIPEA (98 μL, 0.564 mmol) and reaction mixture was heated at 90 °C for 15h. After completion of reaction, it was cooled to rt, and water was added. The aq. layer was extracted using EtOAc (3 x 5 mL). All the organic layers were collectively dried over Na_2_SO_4_ and then concentrated. The crude reaction mixture was purified over silica gel column chromatography using MeOH:DCM (1:9) to get the product **47b** as light-yellow gum. ^1^H NMR (400 MHz, *CDCl_3_*) δ 8.03 (s, 1H), 7.52 (d, *J* = 0.8 Hz, 1H), 7.48 (d, *J* = 10.9 Hz, 1H), 7.44 – 7.37 (m, 2H), 7.00 (s, 1H), 6.90 (s, 1H), 6.52 (t, *J* = 55.6 Hz, 1H), 4.93 (dd, *J* = 12.3, 5.2 Hz, 1H), 4.10 (s, 3H), 3.95 (s, 3H), 3.88 – 3.59 (m, 6H), 3.06 (t, *J* = 12.1 Hz, 2H), 2.93 – 2.72 (m, 7H), 2.44 (d, *J* = 11.4 Hz, 2H), 2.19 – 2.02 (m, 5H), 1.59 (s, 9H). LC-MS: m/z 842.0 (M+1).

###### Procedure for synthesis of dCBP-30:

Compound **47b** (43mg, 0.051 mmol) was dissolved in 4M HCl in 1,4-dioxane (1.5 mL) and stirred for 12h. After completion of reaction, solvent was evaporated, and crude product was azeotrope using toluene (3 x 1 mL) and resulted crude product was used as such for the next step. LC-MS: m/z 742.0 (M+1).

The crude product was dissolved in DCM (1.5mL); triethylamine (70 μL, 0.499 mmol) and N-methyl-1H-imidazole-1-carboximide **9** (12.5 mg, 0.10 mmol) was added at rt and stirred overnight. After completion of reaction, volatiles were evaporated and the crude reaction mixture was purified by C18 column chromatography using acetonitrile water (1:1) to yield corresponding compounds **dCBP-30** as yellow solid (9.7mg, 24%). ^1^H NMR (400 MHz, *CD_3_OD*) δ 7.63 (d, *J* = 0.8 Hz, 1H), 7.58 – 7.51 (m, 2H), 7.50 (d, *J* = 0.8 Hz, 1H), 7.08 (t, *J* = 1.1 Hz, 1H), 6.75 (s, 1H), 6.58 (t, *J* = 55.5 Hz, 1H), 5.09 (dd, *J* = 12.4, 5.5 Hz, 1H), 4.34 (tt, *J* = 11.5, 4.0 Hz, 1H), 4.09 (s, 2H), 3.93 (s, 3H), 3.83 (d, *J* = 12.6 Hz, 2H), 3.74 (t, *J* = 5.7 Hz, 2H), 3.71 – 3.65 (m, 2H), 3.13 (dd, *J* = 13.4, 11.0 Hz, 2H), 2.92 – 2.72 (m, 7H), 2.69 (s, 4H), 2.35 (qd, *J* = 12.4, 4.0 Hz, 2H), 2.16 – 2.00 (m, 6H). ^13^C NMR (101 MHz, *CD_3_OD*) δ 174.6, 171.4, 168.4, 167.9, 167.9, 160.8, 160.8, 158.2, 150.2, 147.2, 147.1, 143.8, 139.8, 139.5, 132.1, 131.2, 131.1, 131.0, 130.8, 130.5, 130.5, 127.3, 125.5, 125.4, 122.3, 122.2, 122.2, 120.9, 117.4, 115.0, 115.0, 112.8, 112.7, 112.6, 111.2, 111.2, 108.2, 56.6, 50.9, 50.7, 49.0, 42.0, 41.7, 38.9, 32.8, 32.2, 28.5, 27.7, 23.7, 23.3, 23.0. HRMS (ESI) calc’d for C_40_H_41_F_3_N_10_O_5_+H = 799.3286, found 799.3285.

###### Procedure for synthesis of dCBP-30-Bump:

Intermediate **47b** (70mg, 0.083 mmol) was dissolved in dry DMF (1.5 mL) and to this solution, Methyl iodide (47.2mg, 0.333 mmol) and solid K_2_CO_3_ (46mg, 0.333 mmol) were added sequentially at rt. Reaction mixture was stirred at rt for 15h and after completion of reaction, it was diluted with water (5mL) and aqueous layer was extracted with ethyl acetate (3 x 5 mL). Combined organic layers were dried over anhydrous Na_2_SO_4_ and the solvent was evaporated, and crude product was purified by silica gel column chromatography using EtOAc:Hexanes to yield compound **48** as yellow gum (43mg, 60%). LC-MS: m/z 856.0 (M+1).

Compound **48** (43mg, 0.050 mmol) was dissolved in 4M HCl in 1,4-dioxane (1.5 mL) and stirred for 12h. After completion of reaction, solvent was evaporated, and crude product was azeotrope using toluene (3 x 1 mL) and resulted crude product was used as such for the next step. The crude product **48** was dissolved in DCM (1.5mL); triethylamine (74 μL, 0.53 mmol) and N-methyl-1H-imidazole-1-carboximide **9** (20mg, 0.159 mmol) was added at rt and stirred overnight. After completion of reaction, volatiles evaporated, and the crude reaction mixture was purified by C18 column chromatography using acetonitrile water (1:1) to yield corresponding compounds **dCBP-30-Bump** as yellow solid (13mg, 32%). ^1^H NMR (500 MHz, *CD_3_OD*) δ 7.53 (s, 1H), 7.47 (d, *J* = 10.7 Hz, 1H), 7.42 (d, *J* = 8.1 Hz, 2H), 7.04 (s, 1H), 6.86 (s, 1H), 6.54 (t, *J* = 55.6 Hz, 1H), 4.93 (dd, *J* = 12.4, 5.3 Hz, 1H), 4.42 (d, *J* = 4.9 Hz, 1H), 4.14 (t, *J* = 11.3 Hz, 1H), 3.97 (s, 2H), 3.95 (s, 3H), 3.81 (q, *J* = 4.9 Hz, 4H), 3.73 (t, *J* = 5.7 Hz, 2H), 3.20 (s, 3H), 3.09 – 2.95 (m, 3H), 2.86 (t, *J* = 6.5 Hz, 2H), 2.78 (t, *J* = 5.0 Hz, 7H), 2.42 (dd, *J* = 13.2, 9.7 Hz, 2H), 2.13 – 2.01 (m, 5H). ^13^C NMR (126 MHz, *CD_3_OD*) δ 171.2, 168.9, 168.9, 167.3, 166.7, 159.3, 158.4, 157.2, 148.6, 145.8, 145.8, 142.1, 138.9, 137.5, 131.0, 129.7, 129.5, 129.4, 129.1, 129.0, 126.5, 124.2, 124.2, 121.3, 121.3, 119.5, 110.4, 106.1, 77.4, 77.2, 76.9, 55.4, 50.5, 50.4, 50.2, 50.1, 49.7, 41.4, 40.6, 39.2, 32.1, 31.7, 29.8, 28.0, 27.7, 27.4, 27.4, 22.5, 22.4. HRMS (ESI) calc’d for C_41_H_43_F_3_N_10_O_5_+H = 813.3443, found 813.3453.

### QUANTIFICATION AND STATISTICAL ANALYSIS

All statistical analyses were performed using GraphPad Prism unless stated otherwise. Statistical analyses other than two-tailed unpaired Student’s *t*-tests are indicated in the figure legends. Quantitative data are presented as mean ± standard error of the mean (SEM) and a *p* value of <0.05 was considered statistically significant unless stated otherwise.

## Supplementary Material

1

2

3

4

5

6

7

8

Supplemental information can be found online at https://doi.org/10.1016/j.celrep.2026.117464.

## Figures and Tables

**Table:** KEY RESOURCES TABLE

REAGENT or RESOURCE	SOURCE	IDENTIFIER
Antibodies		
Vinculin	Bethyl	Cat# A302-535A; RRID: AB_1999080
CBP	Cell Signaling Technology	Clone D6C5; RRID: AB_2616020
p300	Cell Signaling Technology	Clone D2X6N; RRID: AB_2799450
GAPDH	Cell Signaling Technology	Clone 14C10; RRID: AB_561053
CRBN	Sigma	Cat# HPA045910; RRID: AB_10960409
MYC	Santa Cruz	Clone 9E10; RRID: AB_631276
BRD4	Bethyl	Cat# A301-9851; RRID: AB_1576498
IRF4	Cell Signaling Technology	Clone P173; RRID: AB_2208963
PARP	Cell Signaling Technology	Cat# 9542S; RRID: AB_2160739
H2K5ac	Cell Signaling Technology	Clone D5H1S; RRID: AB_2636805
H2BK20ac	Cell Signaling Technology	Clone D7O9W; RRID: AB_2799047
Total H2B	Active motif	Cat# 39210; RRID: AB_2793185
H3K27ac	Abcam	Cat# ab4729; RRID: AB_2118291
H3K18ac	Active motif	Cat# 39755; RRID: AB_2714186
H3	Cell Signaling Technology	Clone 96C10; RRID: AB_1642229
CBP	Abcam	Clone EPR23418-23; RRID: AB_3676065
p300	Cell Signaling Technology	Clone D8Z4E; RRID: AB_2800077
Anti-Rabbit IgG, HRP-linked antibody	Cell Signaling Technology	Cat# 7074; RRID: AB_2099233
Anti-Mouse IgG, HRP-linked antibody	Cell Signaling Technology	Cat# 7076; RRID: AB_330924
DISCOVERY OmniMap anti-Rabbit HRP	Roche	Cat# 760-4311; RRID: AB_2811043
Chemicals, peptides, and recombinant proteins		
dCBP-1	Vannam et al.^21^ 2012	
dCBP-2	Tiwari et al.^31^ 2024	
dCBP-3	Tiwari et al.^31^ 2024	
dCBP-11	This study	N/A
dCBP-12	This study	N/A
dCBP-13	This study	N/A
dCBP-14	This study	N/A
dCBP-15	This study	N/A
dCBP-25	This study	N/A
dCBP-26	This study	N/A
dCBP-27	This study	N/A
dCBP-28	This study	N/A
dCBP-29	This study	N/A
dCBP-30	This study	N/A
dCBP-30-bump	This study	N/A
GNE-781	MedChem Express	Cat# HY-108696; CAS: 1936422-33-1
A-485	MedChem Express	Cat# 6387; CAS: 1889279-16-6
MLN4929	Cayman Chemical	Cat# 15217; CAS: 905579-51-3
Carfilzomib	MedChem Express	Cat# HY-10455; CAS: 868540-17-4
Pomalidomide	Sigma	Cat# P0018; CAS: 19171-19-8
Lenalidomide	Sigma	Cat# SML2283; CAS: 191732-72-6
Panobinostat	Selleck Chemicals	Cat# S1030; CAS: 404950-80-7
Dexamethasone	Selleck Chemicals	Cat# S1322; CAS: 50-02-2
HALT protease inhibitor cocktail, EDTA-free	Pierce	Cat# 87785
cOmplete Mini EDTA-free Protease Inhibitor Cocktail	Sigma	Cat# 11836170001
Endopeptidase Lys-C	Wako	Cat# 121-05063
FLAG-CRBN/DDB1/CUL4A/RBX1	BPS Bioscience	Cat# 100329
GST-CBP	BPS Bioscience	Cat# 31128
His-p300	BPS Bioscience	Cat# 31118
AlphaLISA anti-FLAG acceptor beads	PerkinElmer	Cat# AL1112C
AlphaScreen glutathione donor beads	PerkinElmer	Cat# 6765300
Nickel chelate donor beads	PerkinElmer	Cat# AS101D
TRIzol^™^	Invitrogen	Cat# 15596026
RIPA Lysis buffer	Millipore	Cat# 20-188
APC-conjugated Annexin-V	BD Bioscience	Cat# 550475
Drosophila cell nuclei spike-in control	Active motif	Cat# 53154
Critical commercial assays		
CellTiter-Glo	Promega	Cat# G8462
Direct-zol RNA Miniprep kit	Zymo Research	Cat# R2051
Quantseq 3′mRNA-seq V2 Library Prep Kit FWD	Lexogen	Cat# 015.24
66-440 kDa Separation Module	Proteinsimple	Cat# SM-W005
Anti-Rabbit Detection Module	Protiensimple	Cat# DM-001
MinElute PCR purification kit	Qiagen	Cat# 28006
DISCOVERY ChromoMap DAB kit	Roche	Cat# 760-159
Deposited data		
SLAM-seq	This study	GEO: GSE318522
ATAC-seq	This study	GEO: GSE318521
Proteomics	This study	MassIVE: MSV000101009
^1^H and ^13^C NMR spectra of compounds	This study	Data S1
Experimental models: cell lines		
MM1.S	ATCC	RRID: CVCL_8792
MM1.R	ATCC	RRID: CVCL_8794
U266	ATCC	RRID: CVCL_0566
NCI-H929	ATCC	RRID: CVCL_1600
RPMI-8226	ATCC	RRID: CVCL_8226
KMS-11	JCRB	RRID: CVCL_2989
KMS-12-BM	JCRB	RRID: CVCL_1334
KMS-12-PE	JCRB	RRID: CVCL_1333
KMS-20	JCRB	RRID: CVCL_2990
KMS-26	JCRB	RRID: CVCL_2992
KMS-27	JCRB	RRID: CVCL_2993
KMS-34	JCRB	RRID: CVCL_2996
KMM-1	JCRB	RRID: CVCL_2981
OPM-2	DSMZ	RRID: CVCL_1625
L363	DSMZ	RRID: CVCL_1357
MOLP-8	DSMZ	RRID: CVCL_2124
AMO-1	DSMZ	RRID: CVCL_1806
Karpas-620	DSMZ	RRID: CVCL_1823
SKMM2	DSMZ	RRID: CVCL_1699
EJM	DSMZ	RRID: CVCL_2030
LP-1	DSMZ	RRID: CVCL_0012
HAP1 CRBN KO	Vannam et al.^21^ 2012	
Experimental models: organisms/strains		
*C57BL/6J*	Jackson laboratory	RRID: IMSR_JAX:000664
*NOD.Cg-Prkdc^scid^ Il2rg^tm1WJI^/SzJ*	Jackson laboratory	RRID: IMSR_JAX:005557
*C57BL/6-Crbn^tm2.1Ble^/J*	Jackson laboratory	RRID: IMSR_JAX:035831
Software and algorithms		
GraphPad Prism	GraphPad	graphpad.com
TrimGalore!	Krueger et al., 2021	github.com/FelixKrueger/TrimGalore
Slamdunk	Herzog et al.,^52^ 2017	t-neumann.github.io/slamdunk/
R (v4.4.2)		r-project.org
deepTools (v3.5.4)	Ramirez et al., 2016	deeptools.readthedocs.io
DESeq2	Love et al.,^53^ 2014	bioconductor.org
EnrichR	Chenet et al., 2013/Kuleshov et al.,^54^ 2016 if web server was used	https://maayanlab.cloud/Enrichr/
Bowtie2	Langmead and Salzberg,^55^ 2012	bowtie-bio.sourceforge.net
BAMtools (v2.5.2)	Barnett et al., 2011	github.com/pezmaster31/bamtools
NGSUtils (v0.5.9)	Breese and Liu, 2013	ngsutils.org
Picard	Broad Institute	broadinstitute.github.io/picard/
MACS2 (v2.2.9.1)	Zhang et al.,^56^ 2008	github.com/macs3-project/MACS
QuPath (v0.6.0)	Bankhead et al.,^57^ 2017	qupath.github.io
SynergyFinder	Zheng et al.,^58^ 2022	synergyfinder.org
ChemDraw	PerkinElmer	revvitysignals.com/products/research/chemdraw
mnova	Mestrelab Research	mestrelab.com/main-product/mnova
Morpheus	Broad Institute	software.broadinstitute.org/morpheus/
chromVAR (v3.23)	Schep et al.,^59^ 2017	bioconductor.org

## Highlights

- Comparative analysis of cancer cell line killing by CBP/p300 inhibitors and degraders
- Design of dCBP-30, an orally bioactive CBP/p300 degrader with near-linkerless design
- Assessment of multiple myeloma transcriptional signaling targeted by dCBP-30
- Demonstration of*in vivo* tolerability and efficacy of dCBP-30 in myeloma xenografts
