# Supplementary material for: An orally active dual CBP/p300 degrader targets core dependencies of multiple myeloma
Source: Cell Rep. Author manuscript; Available in PMC 2026 Jul 14. (PMC13366507; doi:10.1016/j.celrep.2026.117464)
Supplement: 1 [file NIHMS2190731-supplement-1.pdf]

Cell Reports, Volume *45*

Supplemental information

An orally active dual CBP/p300 degrader targets core dependencies of multiple myeloma

Praveen Kumar Tiwari, Bomin Ku, Drew A. Harrison, Sarah Rizvi, Samuel Ojeda, Jessica Duffy, Leonie Cluse, Jennifer R. Devlin, Nenad Bartonicek, Olga Motorna, Ann-Sophie Koglin, Barbara Karakyriakou, Kaitlyn Gagnon, Ramya S. Iyer, Regina Egan, Pat Greninger, Lauren Benz, Caroline Greco, Julia Norton, Amruth Kumar, Eric F. Zaniewski, Soroush Hajizadeh, Robert Morris, Genna Mullen, Ajinkya S. Kawale, Sangwon Min, Sai Reddy Doda, Raghu Vannam, Lee Zou, Wilhelm Haas, Abner Louissaint Jr., Ricky W. Johnstone, and Christopher J. Ott

Data S1/Methods S1. Characterization of dCBP analog library.

Structural analysis and confirmation by ^{1}H and ^{13}C NMR.

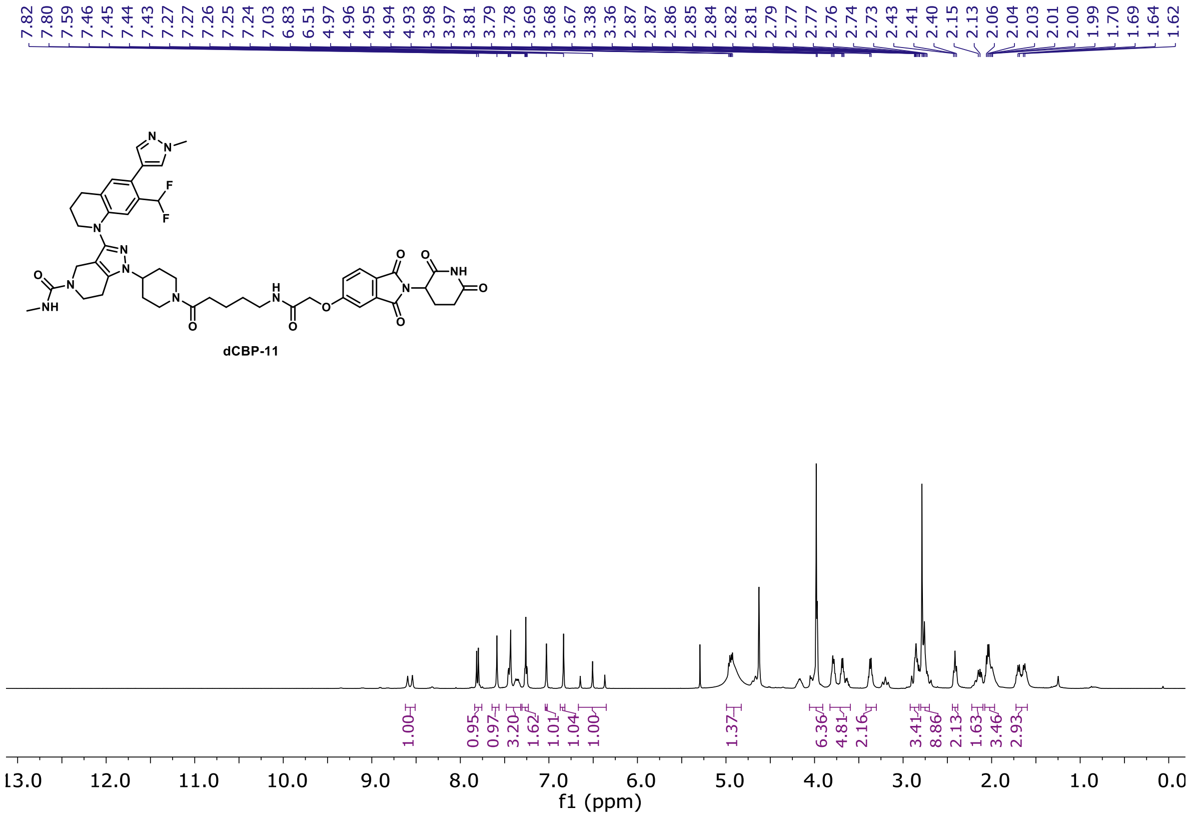

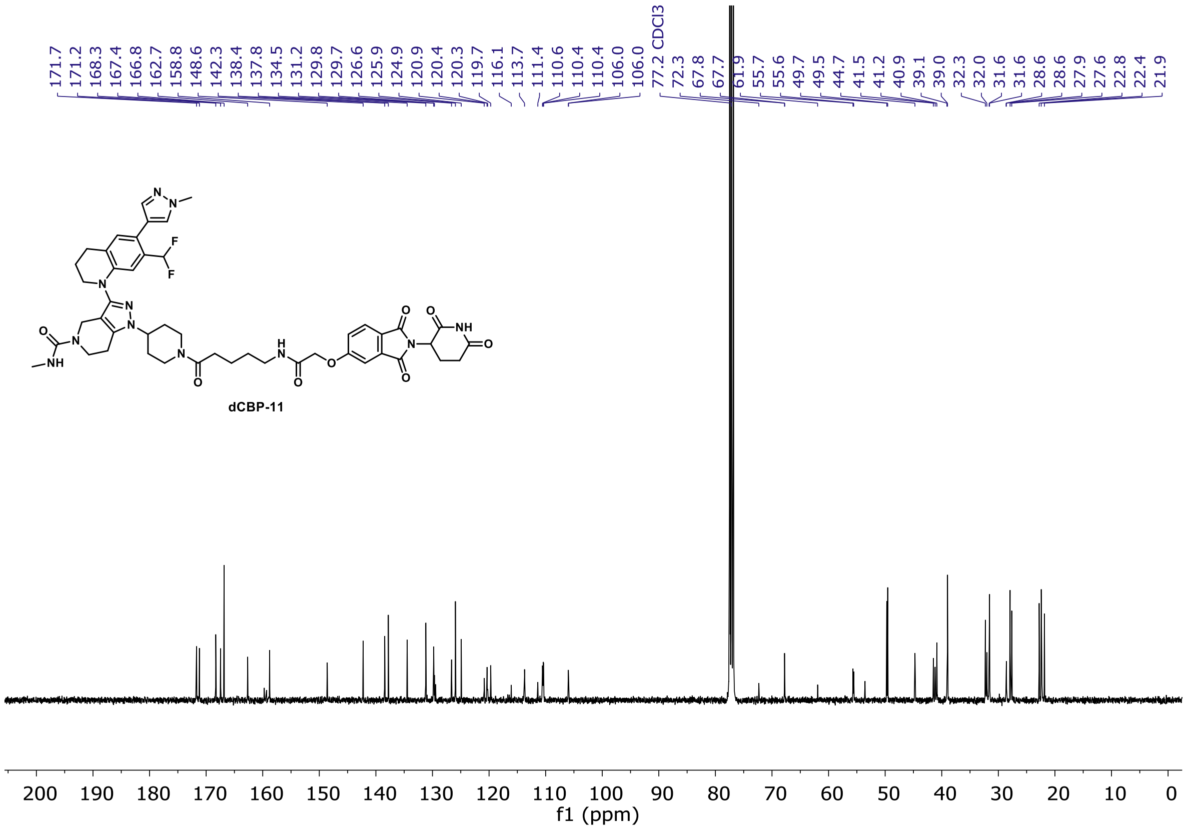

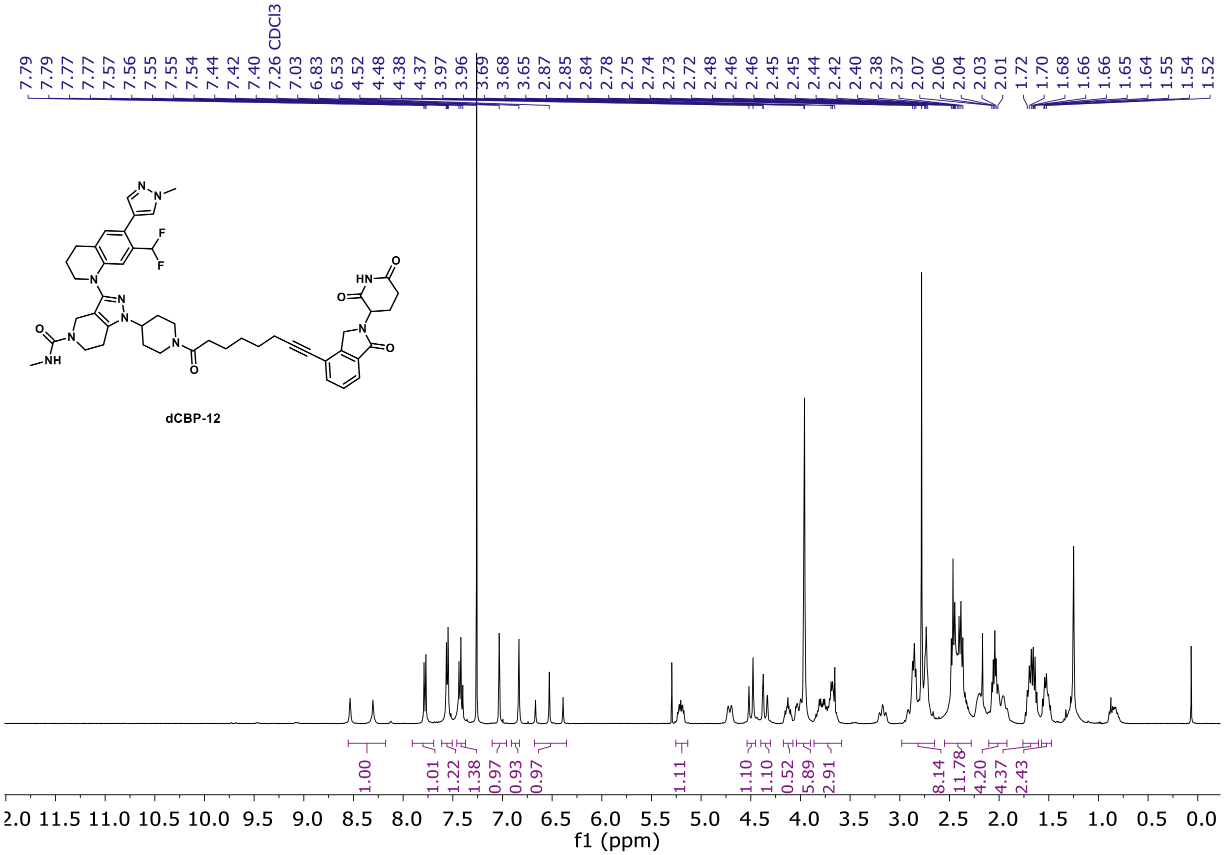
7.79 7.79 7.77 7.77 7.57 7.56 7.55 7.55 7.54 7.44 7.42 7.40 7.26 CDCl3 7.03 6.83 6.53 4.52 4.48 4.38 4.37 3.97 3.96 3.69 3.68 3.65 2.87 2.85 2.84 2.78 2.75 2.74 2.73 2.72 2.48 2.46 2.46 2.45 2.45 2.44 2.42 2.40 2.38 2.37 2.07 2.06 2.04 2.03 2.01 1.72 1.70 1.68 1.66 1.66 1.65 1.64 1.55 1.54 1.52
dCBP-12
1.00 1.01 1.22 1.38 0.97 0.93 0.97 1.11 1.10 1.10 0.52 5.89 2.91 8.14 11.78 4.20 4.37 2.43
f1 (ppm)

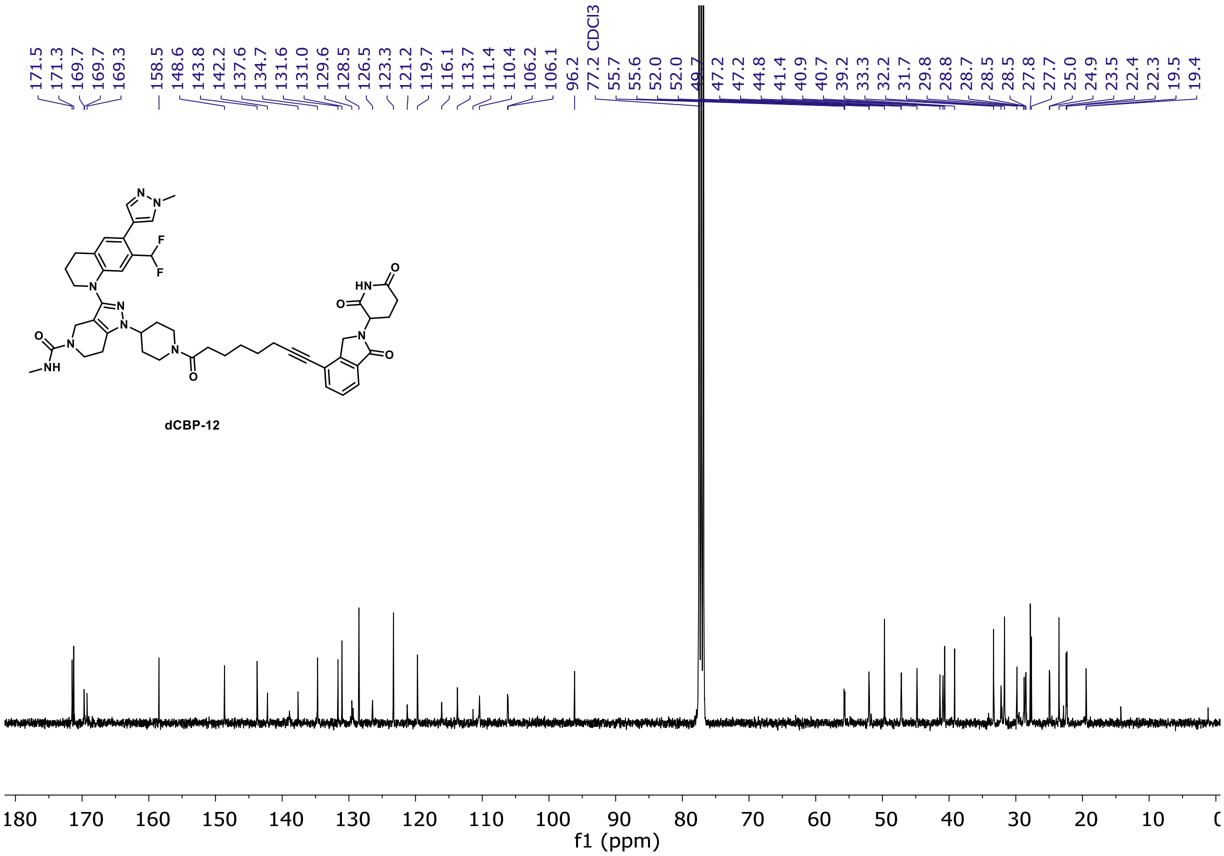
171.5 171.3 169.7 169.7 169.3 158.5 148.6 143.8 142.2 137.6 134.7 131.6 131.0 129.6 128.5 126.5 123.3 121.2 119.7 116.1 113.7 111.4 110.4 106.2 106.1 96.2 77.2 CDCl3 55.7 55.6 52.0 52.0 49.7 47.2 47.2 44.8 41.4 40.9 40.7 39.2 33.3 32.2 31.7 29.8 28.8 28.7 28.5 28.5 27.8 27.7 25.0 24.9 23.5 22.4 22.3 19.5 19.4
dCBP-12
f1 (ppm)

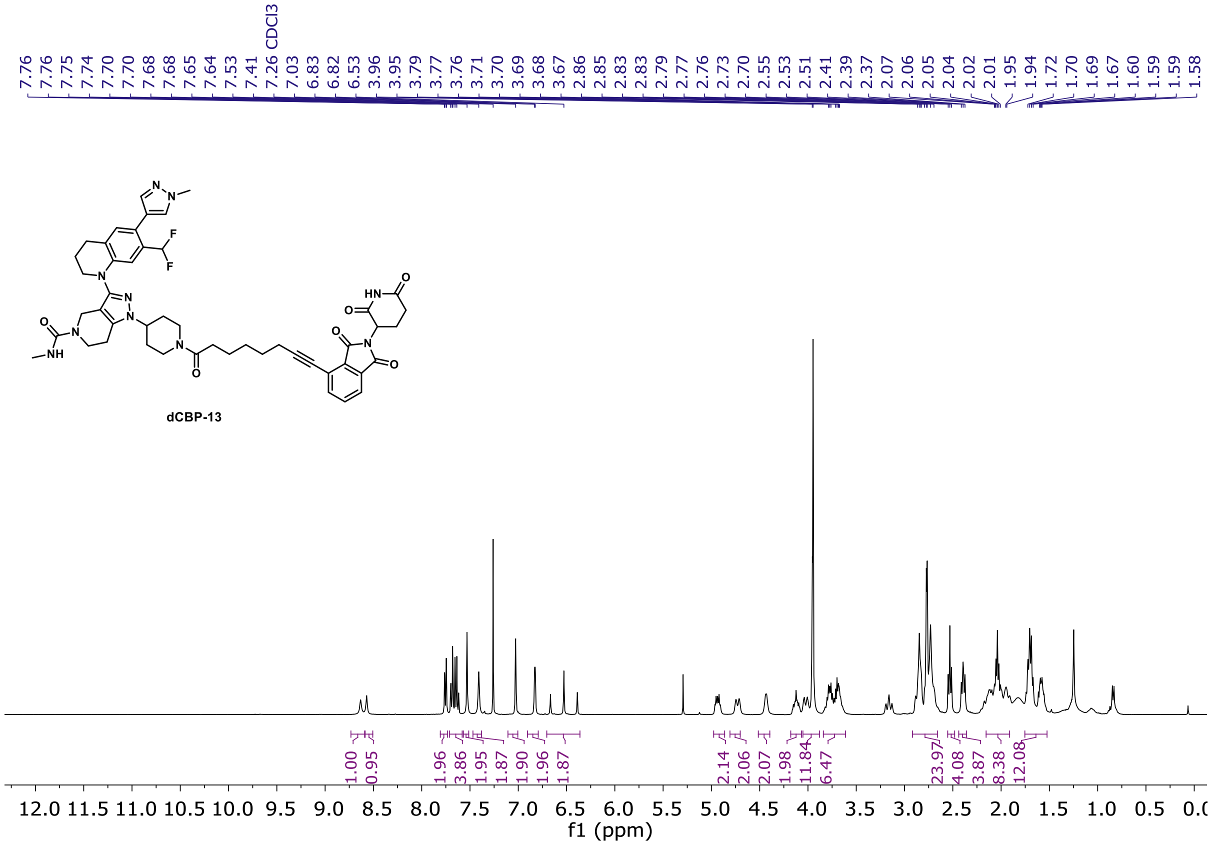
7.76 7.76 7.75 7.74 7.70 7.70 7.68 7.68 7.65 7.64 7.53 7.41 7.26 CDCl3 7.03 6.83 6.82 6.53 3.96 3.95 3.79 3.77 3.76 3.71 3.70 3.69 3.68 3.67 2.86 2.85 2.83 2.83 2.79 2.77 2.76 2.73 2.70 2.55 2.53 2.51 2.41 2.39 2.37 2.07 2.06 2.05 2.04 2.02 2.01 1.95 1.94 1.72 1.70 1.69 1.67 1.60 1.59 1.59 1.58
dCBP-13
1.00 0.95 1.96 3.86 1.95 1.87 1.90 1.96 1.87 2.14 2.06 2.07 1.98 11.84 6.47 23.97 4.08 3.87 8.38 12.08
12.0 11.5 11.0 10.5 10.0 9.5 9.0 8.5 8.0 7.5 7.0 6.5 6.0 5.5 5.0 4.5 4.0 3.5 3.0 2.5 2.0 1.5 1.0 0.5
f1 (ppm)

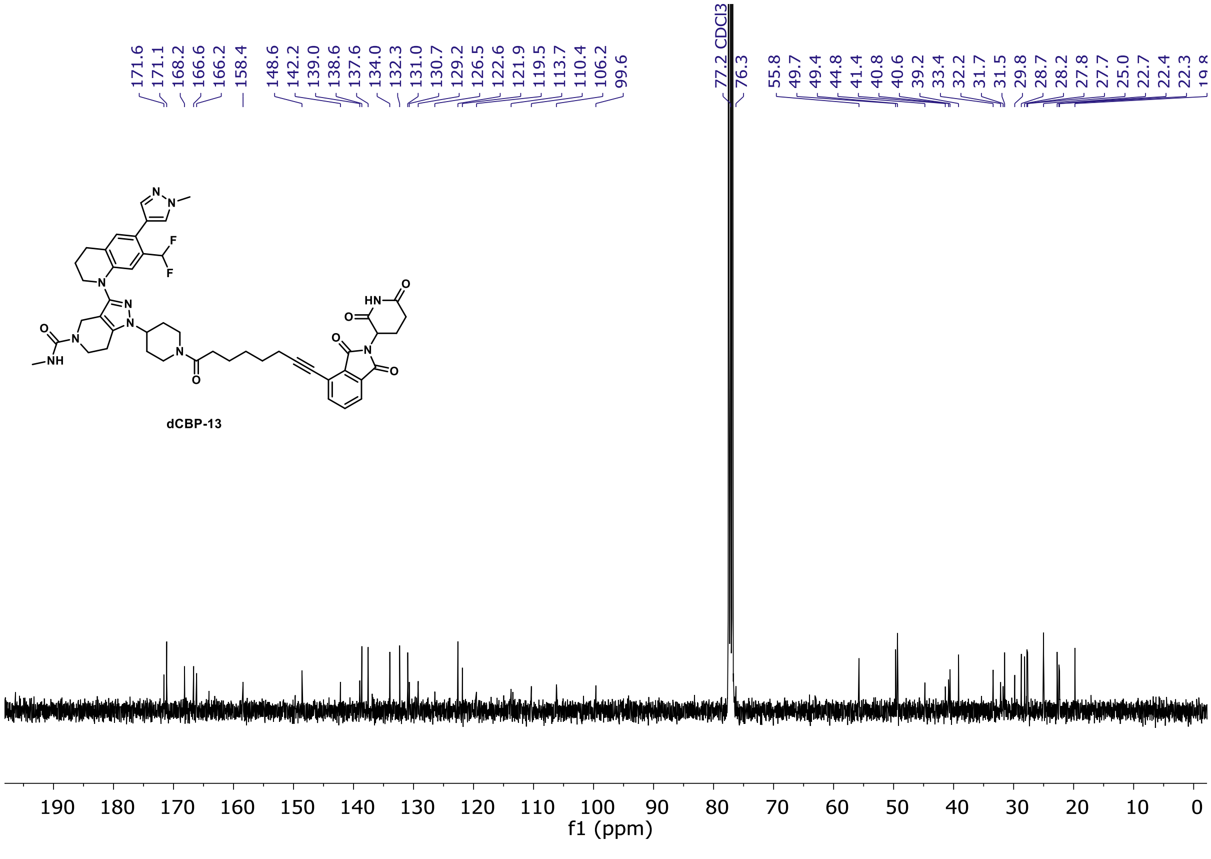
171.6 171.1 168.2 166.6 166.2 158.4 148.6 142.2 139.0 138.6 137.6 134.0 132.3 131.0 130.7 129.2 126.5 122.6 121.9 119.5 113.7 110.4 106.2 99.6 77.2 CDCl3 76.3 55.8 49.7 49.4 44.8 41.4 40.8 40.6 39.2 33.4 32.2 31.7 31.5 29.8 28.7 28.2 27.8 27.7 25.0 22.7 22.4 22.3
dCBP-13
190 180 170 160 150 140 130 120 110 100 90 80 70 60 50 40 30 20 10 0
f1 (ppm)

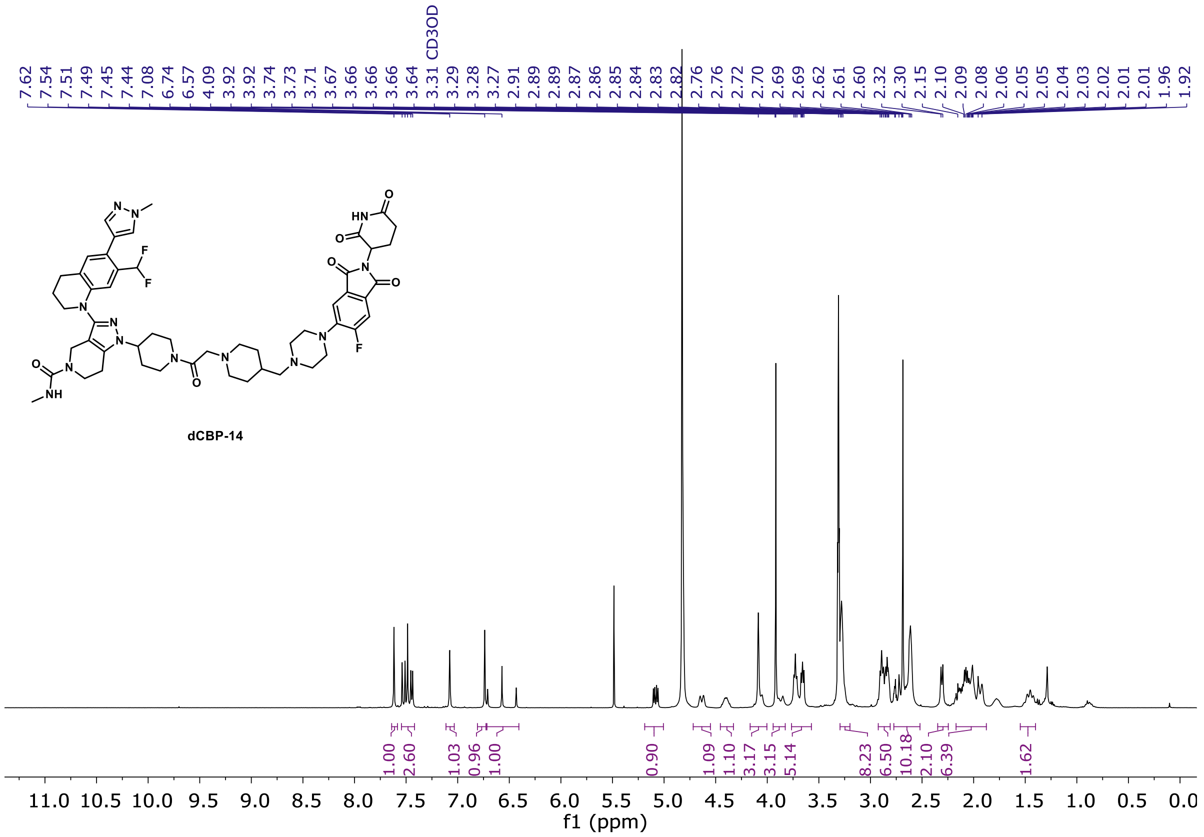
7.62 7.54 7.51 7.49 7.45 7.44 7.08 6.74 6.57 4.09 3.92 3.92 3.74 3.73 3.71 3.67 3.66 3.66 3.64 3.31 CD3OD 3.29 3.28 3.27 2.91 2.89 2.89 2.87 2.86 2.85 2.84 2.83 2.82 2.76 2.76 2.72 2.70 2.69 2.69 2.62 2.61 2.60 2.32 2.30 2.15 2.10 2.09 2.08 2.06 2.05 2.05 2.04 2.03 2.02 2.01 2.01 1.96 1.92
dCBP-14
1.00 2.60 1.03 0.96 1.00 0.90 1.09 1.10 3.17 3.15 5.14 8.23 6.50 10.18 2.10 6.39 1.62
11.0 10.5 10.0 9.5 9.0 8.5 8.0 7.5 7.0 6.5 6.0 5.5 5.0 4.5 4.0 3.5 3.0 2.5 2.0 1.5 1.0 0.5 0.0
f1 (ppm)

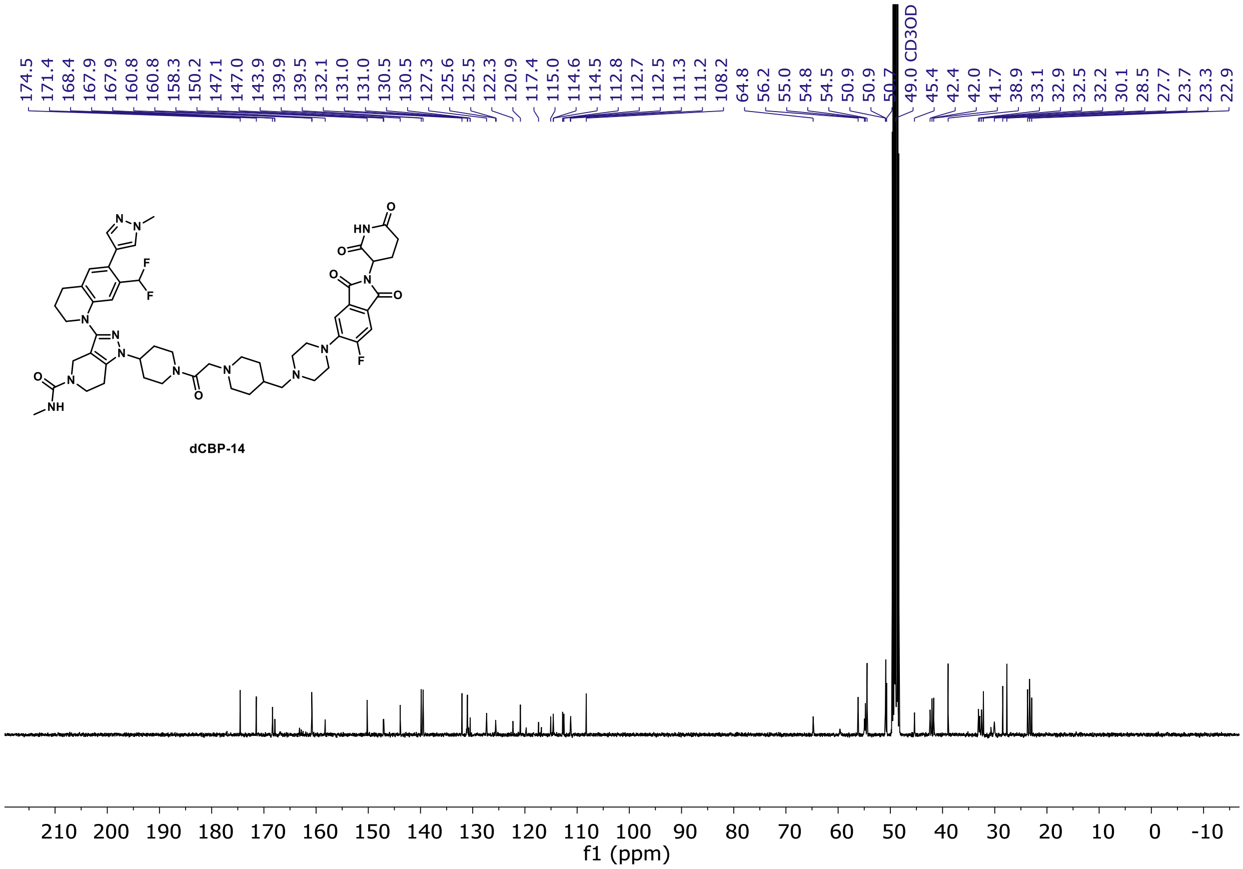
174.5 171.4 168.4 167.9 167.9 160.8 160.8 158.3 150.2 147.1 147.0 143.9 139.9 139.5 132.1 131.0 131.0 130.5 130.5 127.3 125.6 125.5 122.3 120.9 117.4 115.0 114.6 114.5 112.8 112.7 112.5 111.3 111.2 108.2 64.8 56.2 55.0 54.8 54.5 50.9 50.9 50.7 49.0 CD3OD 45.4 42.4 42.0 41.7 38.9 33.1 32.9 32.5 32.2 30.1 28.5 27.7 23.7 23.3 22.9
dCBP-14
210 200 190 180 170 160 150 140 130 120 110 100 90 80 70 60 50 40 30 20 10 0 -10
f1 (ppm)

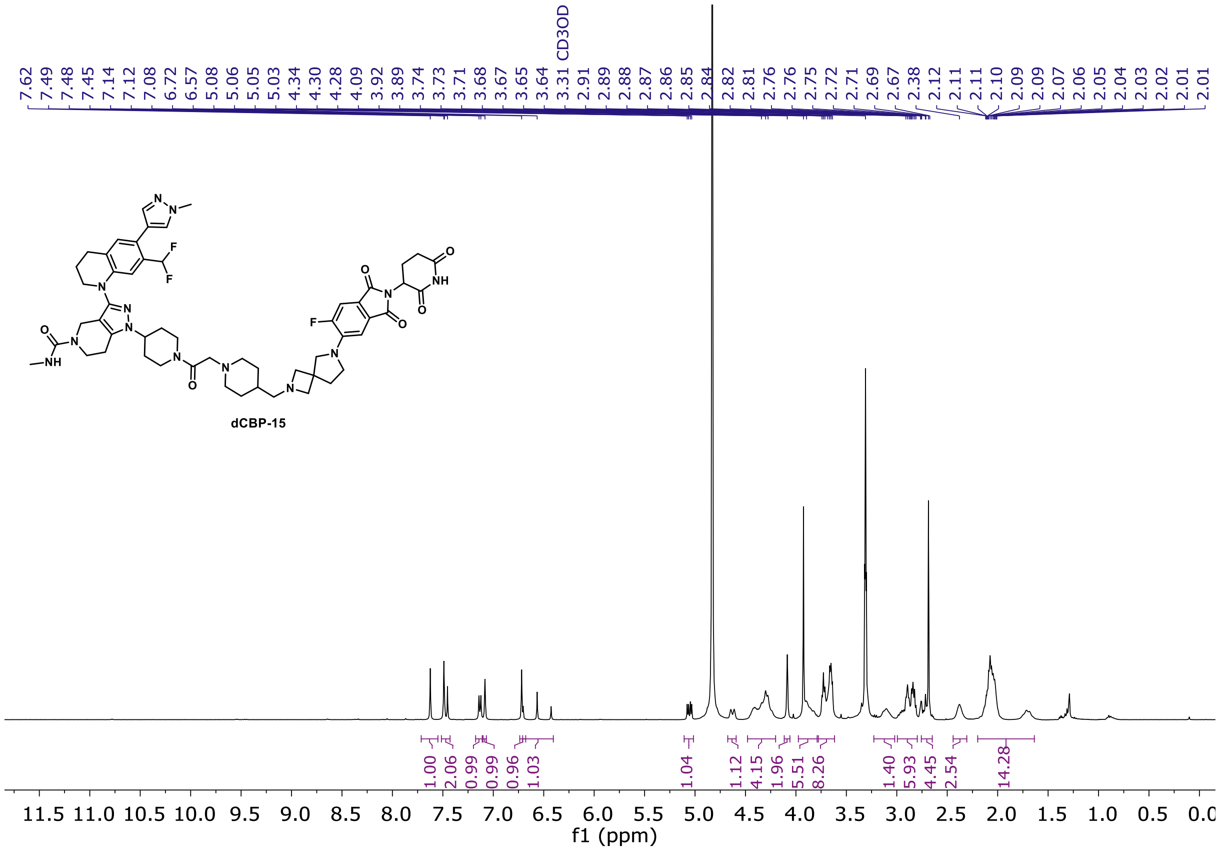
7.62 7.49 7.48 7.45 7.14 7.12 7.08 6.72 6.57 5.08 5.06 5.05 5.03 4.34 4.30 4.28 4.09 3.92 3.89 3.74 3.73 3.71 3.68 3.67 3.65 3.64 3.31 CD3OD 2.91 2.89 2.88 2.87 2.86 2.85 2.84 2.82 2.81 2.76 2.75 2.72 2.71 2.69 2.67 2.38 2.12 2.11 2.11 2.10 2.09 2.09 2.07 2.06 2.05 2.04 2.03 2.02 2.01 2.01
F
F
N
N
O
NH
N
N
N
N
N
O
N
N
F
O
O
N
O
O
NH
N
N
dCBP-15
1.00 2.06 0.99 0.99 0.96 1.03 1.04 1.12 4.15 1.96 5.51 8.26 1.40 5.93 4.45 2.54 14.28
11.5 11.0 10.5 10.0 9.5 9.0 8.5 8.0 7.5 7.0 6.5 6.0 5.5 5.0 4.5 4.0 3.5 3.0 2.5 2.0 1.5 1.0 0.5 0.0
f1 (ppm)

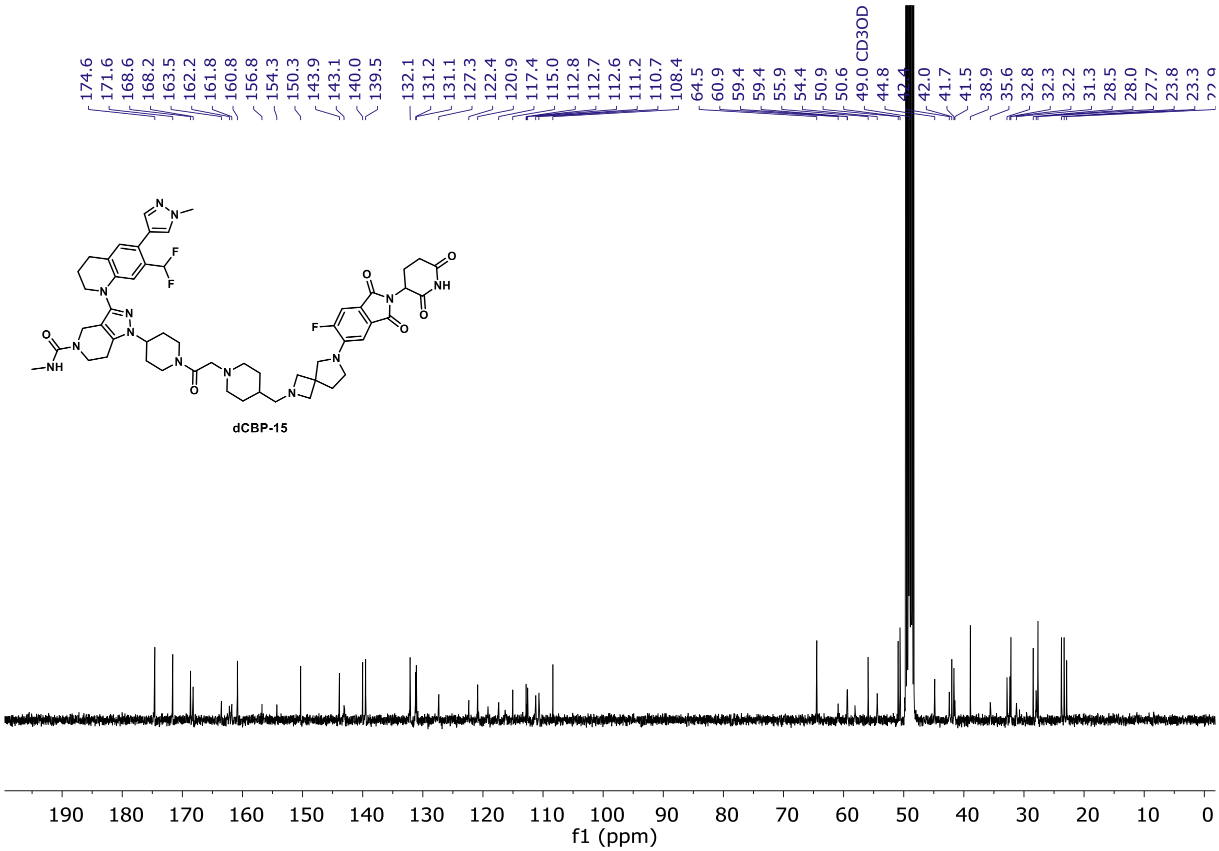
174.6 171.6 168.6 168.2 163.5 162.2 161.8 160.8 156.8 154.3 150.3 143.9 143.1 140.0 139.5 132.1 131.2 131.1 127.3 122.4 120.9 117.4 115.0 112.8 112.7 112.6 111.2 110.7 108.4 64.5 60.9 59.4 55.9 54.4 50.9 50.6 49.0 CD3OD 44.8 42.4 42.0 41.7 41.5 38.9 35.6 32.8 32.3 32.2 31.3 28.5 28.0 27.7 23.8 23.3
dCBP-15
190 180 170 160 150 140 130 120 110 100 90 80 70 60 50 40 30 20 10 0
f1 (ppm)

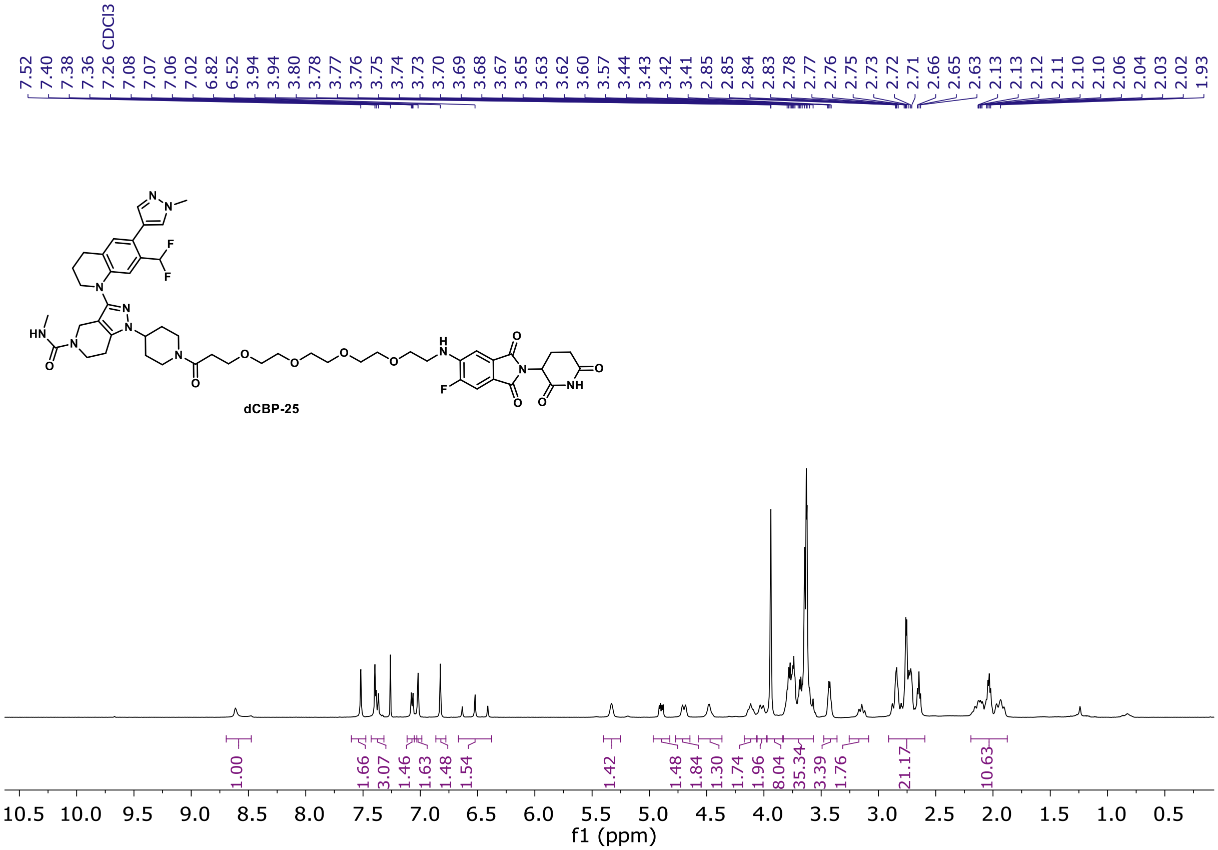
7.52 7.40 7.38 7.36 7.26 CDCl3 7.08 7.07 7.06 7.02 6.82 6.52 3.94 3.94 3.80 3.78 3.77 3.76 3.75 3.74 3.73 3.70 3.69 3.68 3.67 3.65 3.63 3.62 3.60 3.57 3.44 3.43 3.42 3.41 2.85 2.85 2.84 2.83 2.78 2.77 2.76 2.75 2.73 2.72 2.71 2.66 2.65 2.63 2.13 2.13 2.12 2.11 2.10 2.10 2.06 2.04 2.03 2.02 1.93
dCBP-25
1.00 1.66 3.07 1.46 1.63 1.48 1.54 1.42 1.48 1.84 1.30 1.74 1.96 8.04 35.34 3.39 1.76 21.17 10.63
10.5 10.0 9.5 9.0 8.5 8.0 7.5 7.0 6.5 6.0 5.5 5.0 4.5 4.0 3.5 3.0 2.5 2.0 1.5 1.0 0.5
f1 (ppm)

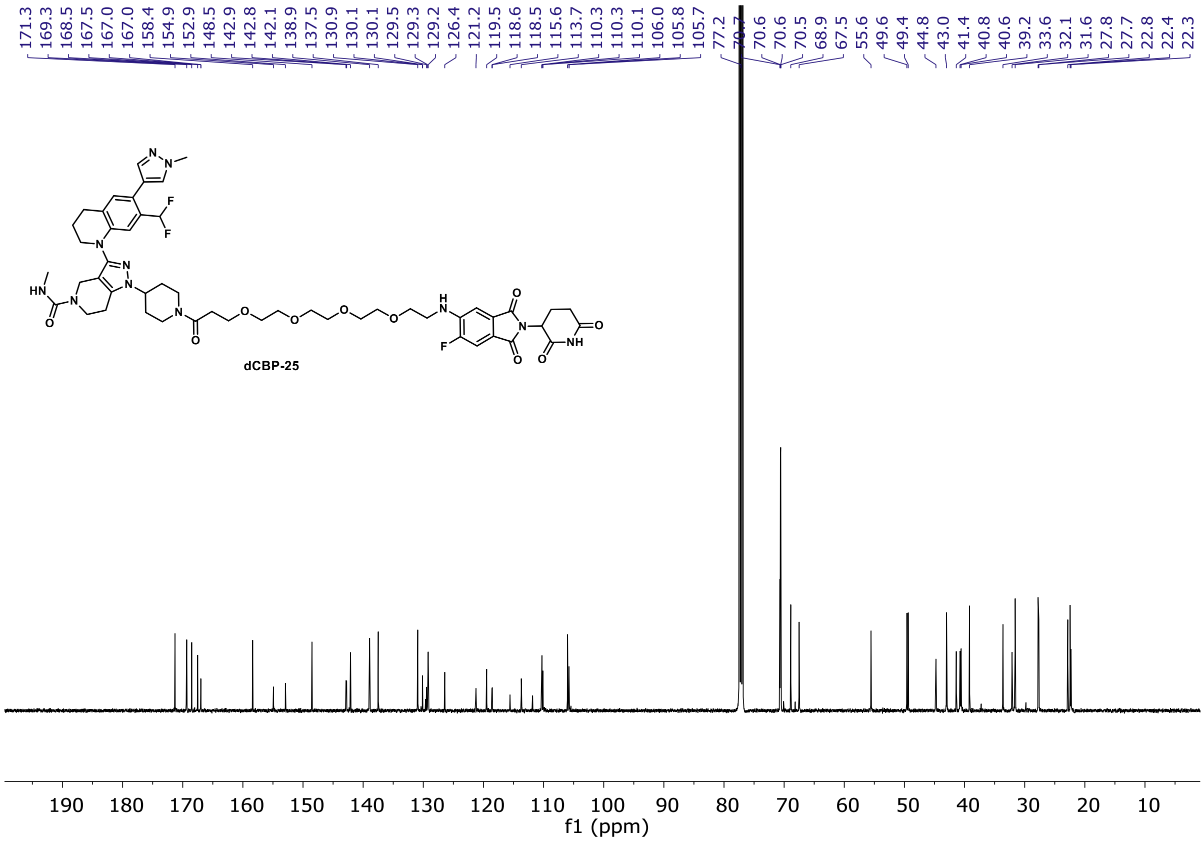
171.3 169.3 168.5 167.5 167.0 167.0 158.4 154.9 152.9 148.5 142.9 142.8 142.1 138.9 137.5 130.9 130.1 130.1 129.5 129.3 129.2 126.4 121.2 119.5 118.6 118.5 115.6 113.7 110.3 110.3 110.1 106.0 105.8 105.7 77.2 70.7 70.6 70.6 70.5 68.9 67.5 55.6 49.6 49.4 44.8 43.0 41.4 40.8 40.6 39.2 33.6 32.1 31.6 27.8 27.7 22.8 22.4 22.3
dCBP-25
190 180 170 160 150 140 130 120 110 100 90 80 70 60 50 40 30 20 10
f1 (ppm)

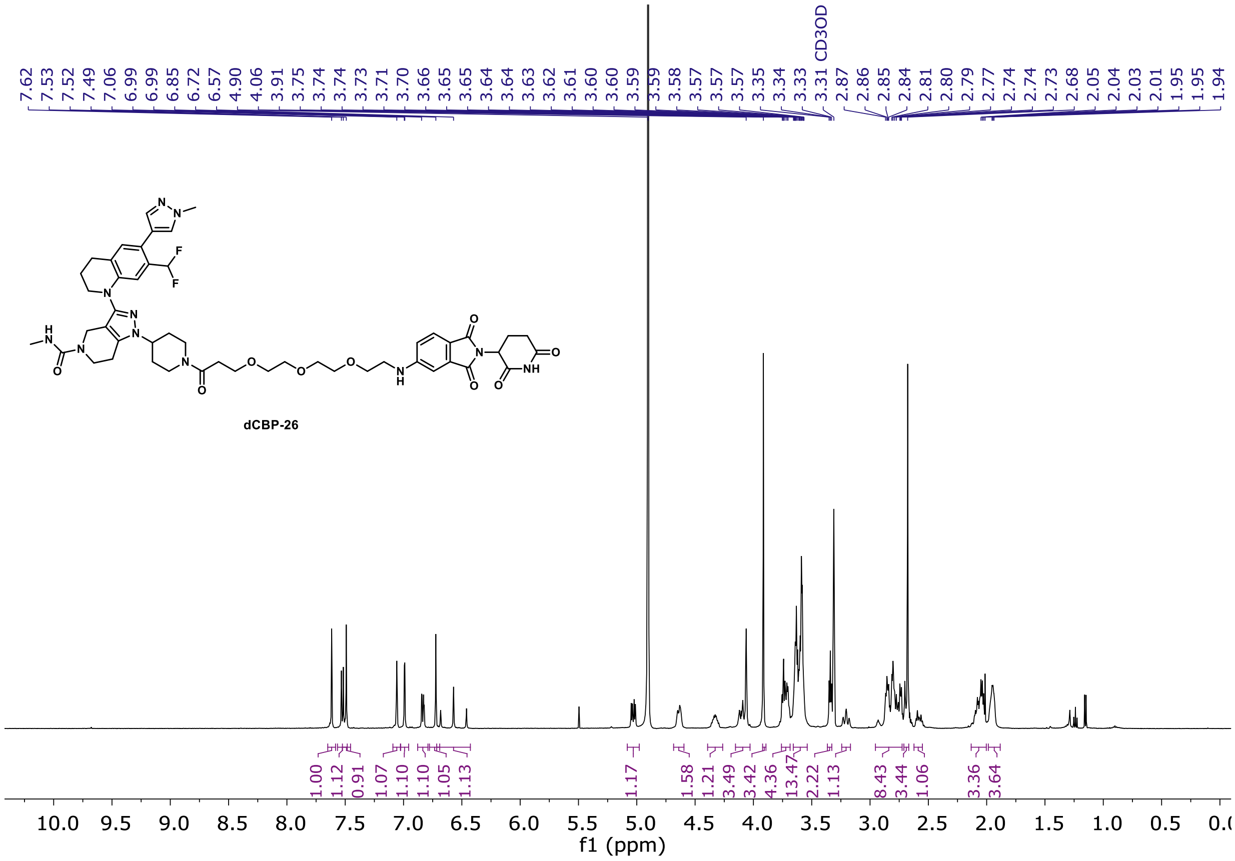
7.62 7.53 7.52 7.49 7.06 6.99 6.99 6.85 6.72 6.57 4.90 4.06 3.91 3.75 3.74 3.74 3.73 3.71 3.70 3.66 3.65 3.65 3.64 3.64 3.63 3.62 3.61 3.60 3.60 3.59 3.59 3.58 3.57 3.57 3.57 3.35 3.34 3.33 3.31 CD3OD 2.87 2.86 2.85 2.84 2.81 2.80 2.79 2.77 2.74 2.74 2.73 2.68 2.05 2.04 2.03 2.01 1.95 1.94
dCBP-26
1.00 1.12 0.91 1.07 1.10 1.10 1.05 1.13 1.17 1.58 1.21 3.49 3.42 4.36 13.47 2.22 1.13 8.43 3.44 1.06 3.36 3.64
10.0 9.5 9.0 8.5 8.0 7.5 7.0 6.5 6.0 5.5 5.0 4.5 4.0 3.5 3.0 2.5 2.0 1.5 1.0 0.5
f1 (ppm)

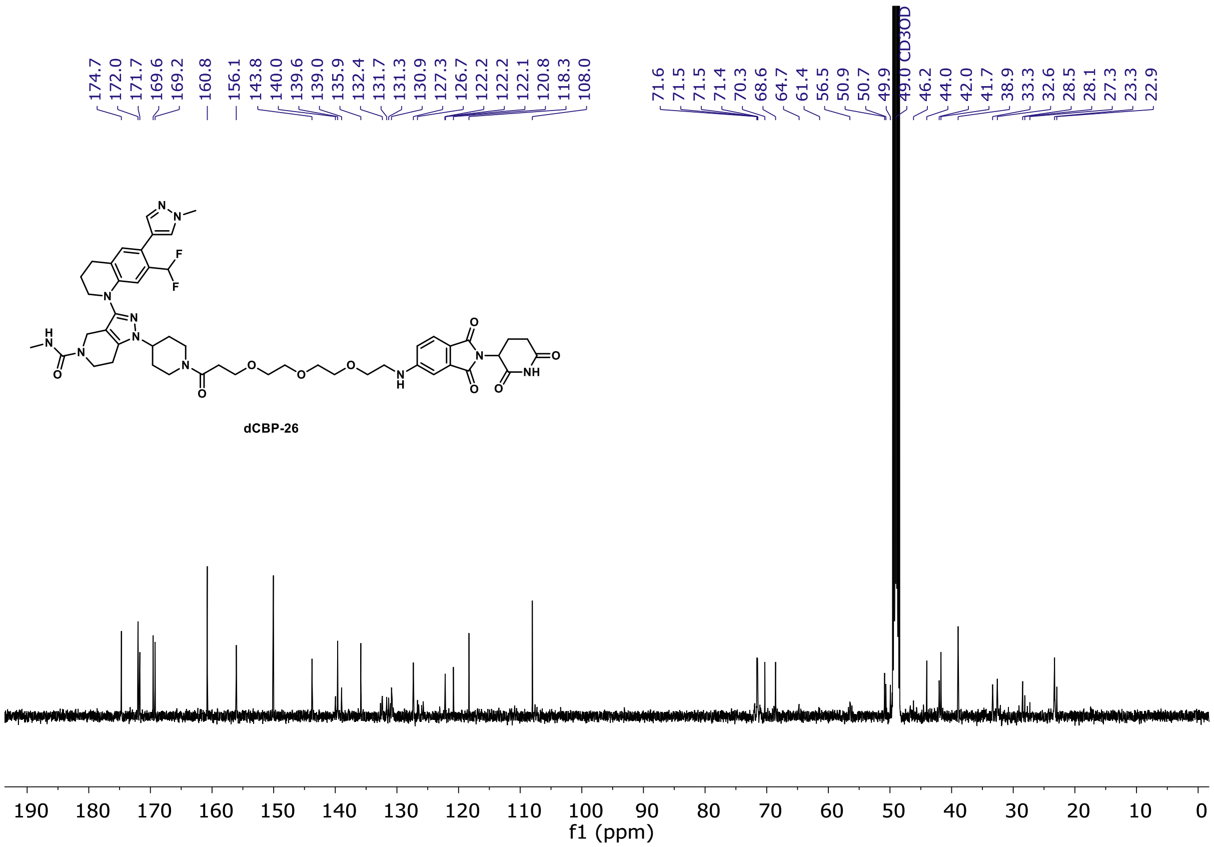
174.7 172.0 171.7 169.6 169.2 160.8 156.1 143.8 140.0 139.6 139.0 135.9 132.4 131.7 131.3 130.9 127.3 126.7 122.2 122.2 122.1 120.8 118.3 108.0 71.6 71.5 71.5 71.4 70.3 68.6 64.7 61.4 56.5 50.9 50.7 49.9 49.0 CD3OD 46.2 44.0 42.0 41.7 38.9 33.3 32.6 28.5 28.1 27.3 23.3 22.9
dCBP-26
190 180 170 160 150 140 130 120 110 100 90 80 70 60 50 40 30 20 10 0
f1 (ppm)

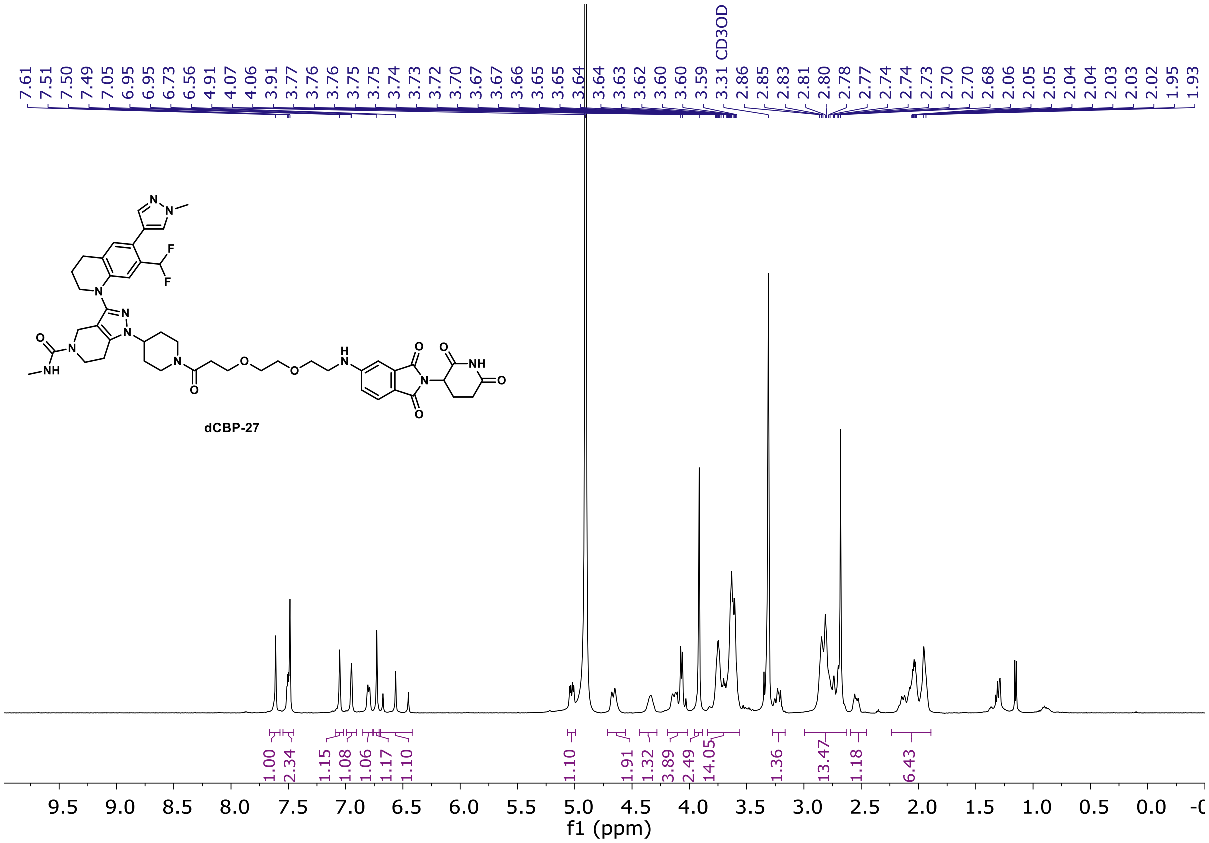
7.61 7.51 7.50 7.49 7.05 6.95 6.95 6.73 6.56 4.91 4.07 4.06 3.91 3.77 3.76 3.76 3.75 3.75 3.74 3.73 3.72 3.70 3.67 3.67 3.66 3.65 3.65 3.64 3.64 3.63 3.62 3.60 3.60 3.59 3.31 CD3OD 2.86 2.85 2.83 2.81 2.80 2.78 2.77 2.74 2.74 2.73 2.70 2.70 2.68 2.06 2.05 2.05 2.04 2.04 2.03 2.03 2.02 1.95 1.93
dCBP-27
1.00 2.34 1.15 1.08 1.06 1.17 1.10 1.10 1.91 1.32 3.89 2.49 14.05 1.36 13.47 1.18 6.43
9.5 9.0 8.5 8.0 7.5 7.0 6.5 6.0 5.5 5.0 4.5 4.0 3.5 3.0 2.5 2.0 1.5 1.0 0.5 0.0
f1 (ppm)

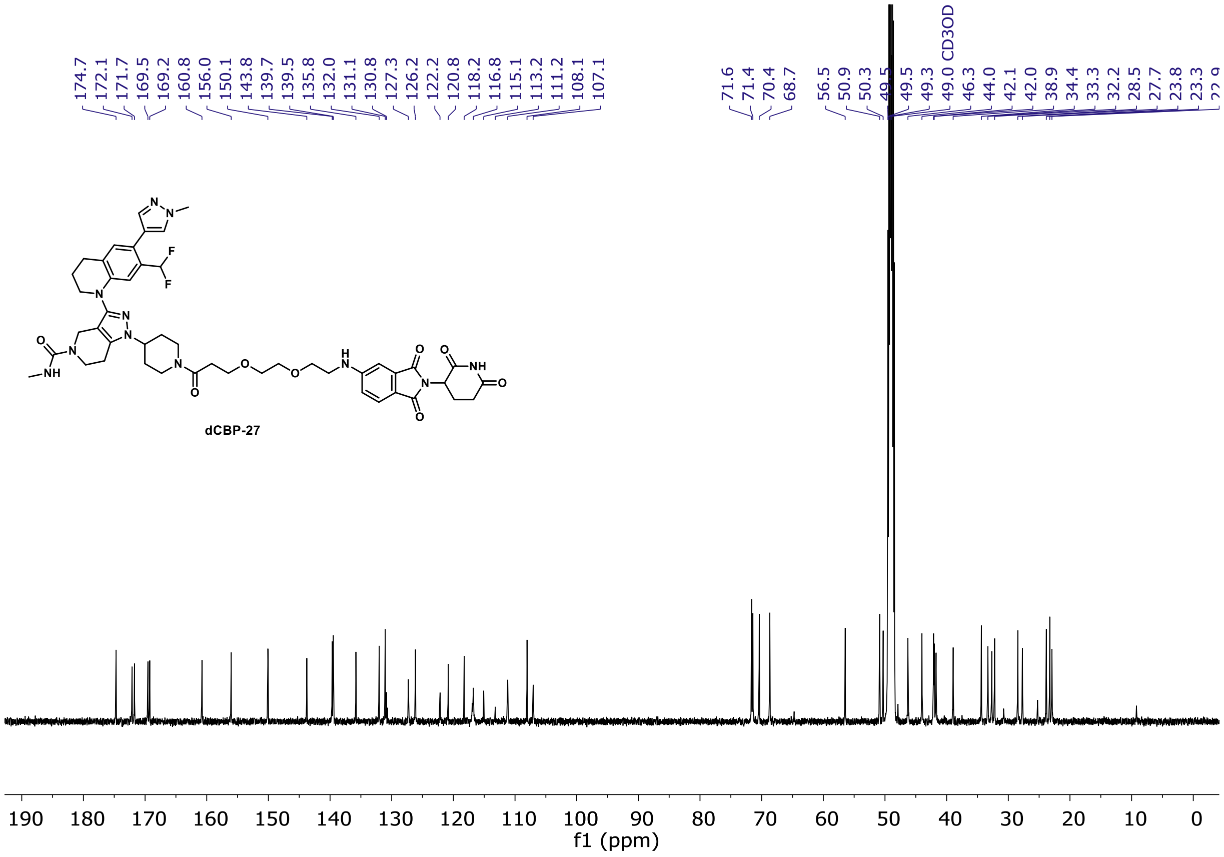
174.7 172.1 171.7 169.5 169.2 160.8 156.0 150.1 143.8 139.7 139.5 135.8 132.0 131.1 130.8 127.3 126.2 122.2 120.8 118.2 116.8 115.1 113.2 111.2 108.1 107.1
71.6 71.4 70.4 68.7 56.5 50.9 50.3 49.5 49.5 49.3 49.0 CD3OD 46.3 44.0 42.1 42.0 38.9 34.4 33.3 32.2 28.5 27.7 23.8 23.3
dCBP-27
190 180 170 160 150 140 130 120 110 100 90 80 70 60 50 40 30 20 10 0
f1 (ppm)

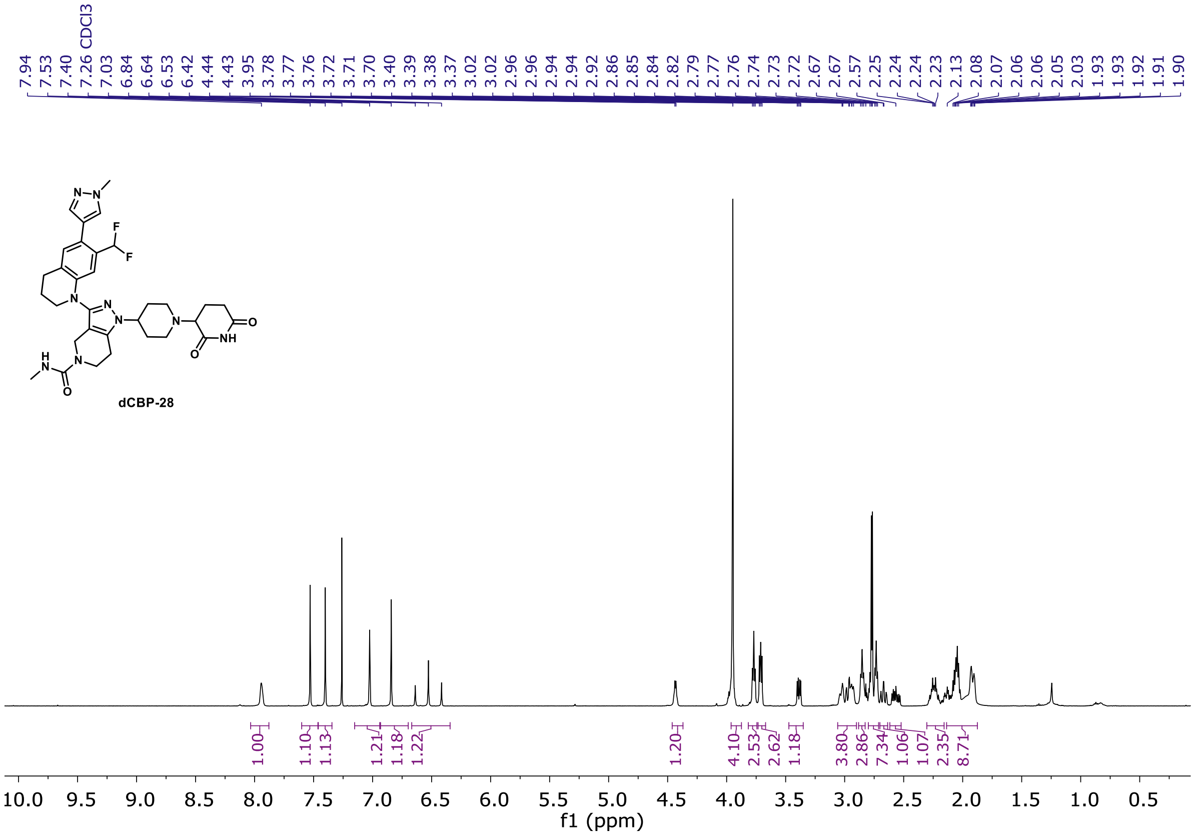
7.94 7.53 7.40 7.26 CDCl3 7.03 6.84 6.64 6.53 6.42 4.44 4.43 3.95 3.78 3.77 3.76 3.72 3.71 3.70 3.40 3.39 3.38 3.37 3.02 3.02 2.96 2.96 2.94 2.94 2.92 2.86 2.85 2.84 2.82 2.79 2.77 2.76 2.74 2.73 2.72 2.67 2.67 2.57 2.25 2.24 2.24 2.23 2.13 2.08 2.07 2.06 2.06 2.05 2.03 1.93 1.93 1.92 1.91 1.90
dCBP-28
1.00 1.10 1.13 1.21 1.18 1.22 1.20 4.10 2.53 2.62 1.18 3.80 2.86 7.34 1.06 1.07 2.35 8.71
10.0 9.5 9.0 8.5 8.0 7.5 7.0 6.5 6.0 5.5 5.0 4.5 4.0 3.5 3.0 2.5 2.0 1.5 1.0 0.5
f1 (ppm)

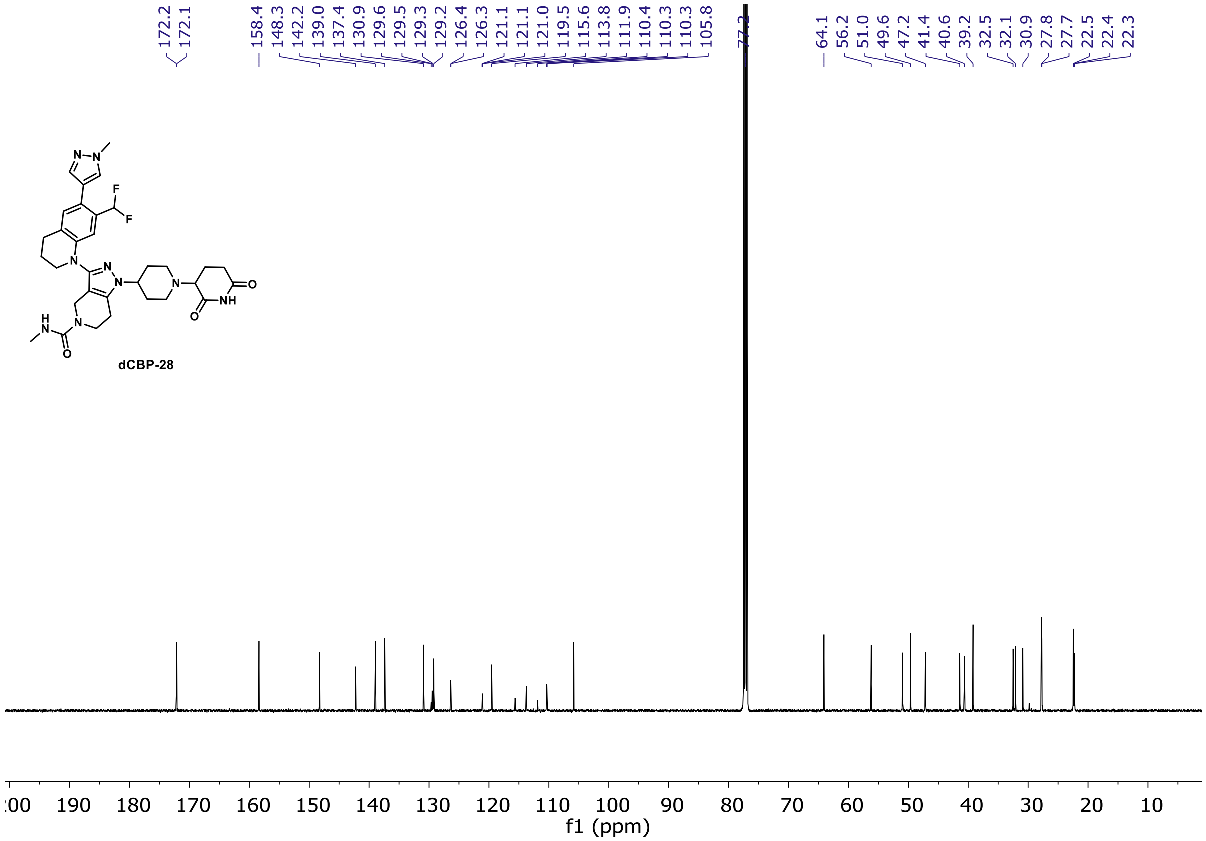
172.2 172.1 158.4 148.3 142.2 139.0 137.4 130.9 129.6 129.5 129.3 129.2 126.4 126.3 121.1 121.1 121.0 119.5 115.6 113.8 111.9 110.4 110.3 105.8 77.2 64.1 56.2 51.0 49.6 47.2 41.4 40.6 39.2 32.5 32.1 30.9 27.8 27.7 22.5 22.4 22.3
dCBP-28
190 180 170 160 150 140 130 120 110 100 90 80 70 60 50 40 30 20 10
f1 (ppm)

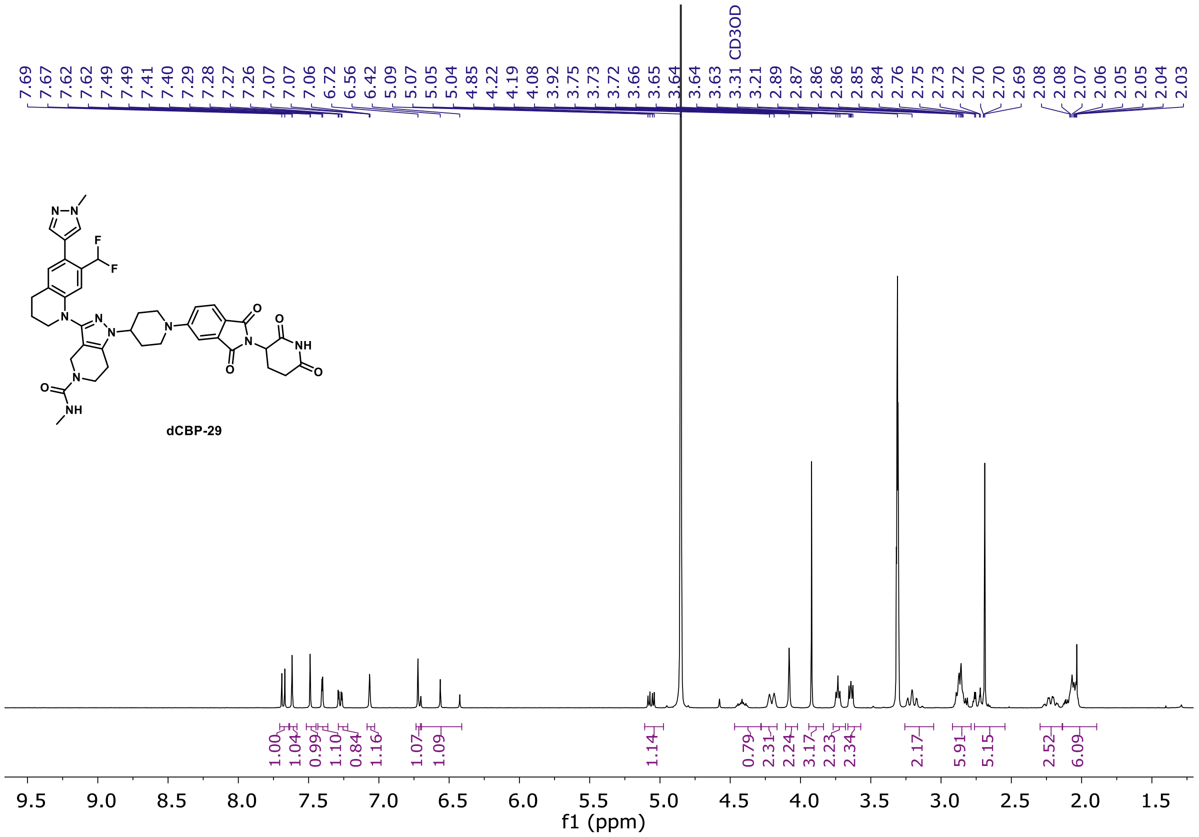
7.69 7.67 7.62 7.62 7.49 7.49 7.41 7.40 7.29 7.28 7.27 7.26 7.07 7.07 7.06 6.72 6.56 6.42 5.09 5.07 5.05 5.04 4.85 4.22 4.19 4.08 3.92 3.75 3.73 3.72 3.66 3.65 3.64 3.64 3.63 3.31 CD3OD 3.21 2.89 2.87 2.86 2.86 2.85 2.84 2.76 2.75 2.73 2.72 2.70 2.70 2.69 2.08 2.08 2.07 2.06 2.05 2.05 2.04 2.03
F
F
N
N
N
N
N
N
N
O
O
O
NH
O
O
NH
dCBP-29
1.00 1.04 0.99 1.10 0.84 1.16 1.07 1.09 1.14 0.79 2.31 2.24 3.17 2.23 2.34 2.17 5.91 5.15 2.52 6.09
9.5 9.0 8.5 8.0 7.5 7.0 6.5 6.0 5.5 5.0 4.5 4.0 3.5 3.0 2.5 2.0 1.5
f1 (ppm)

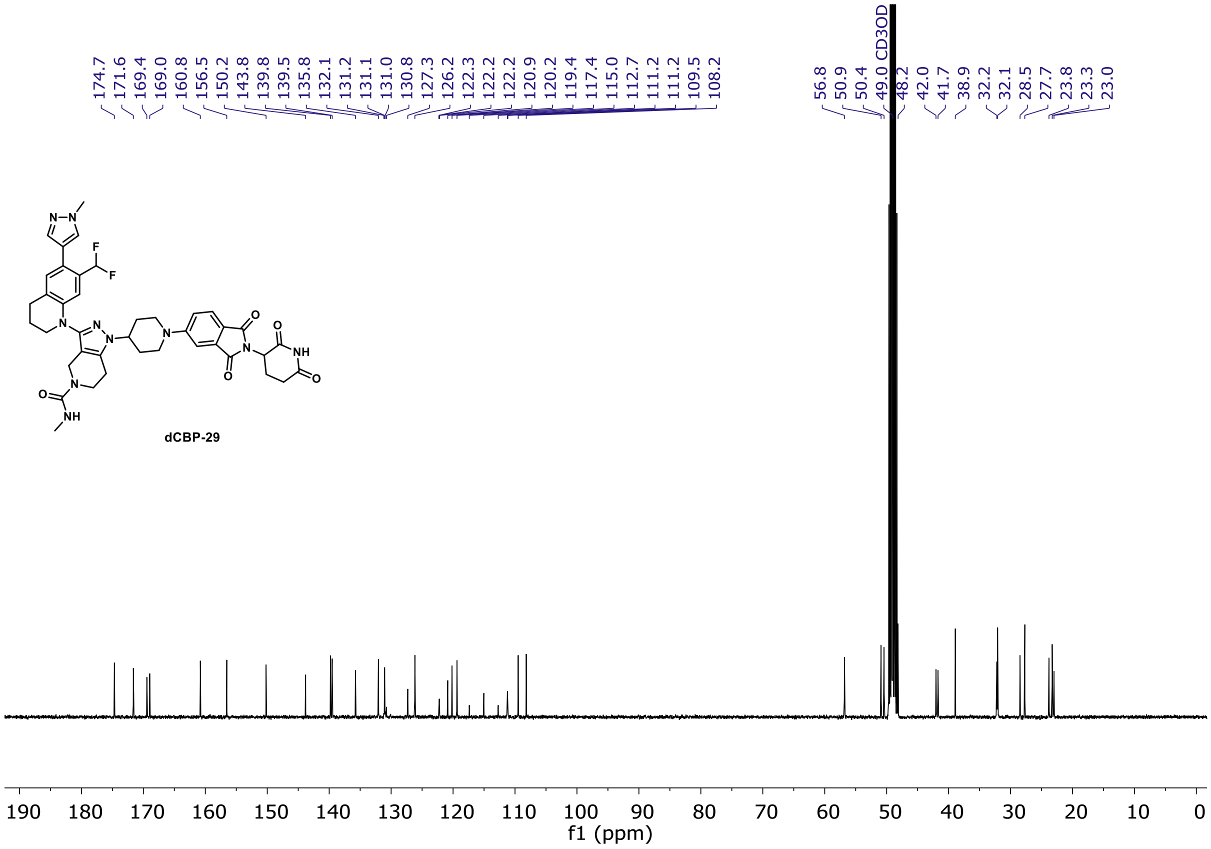
174.7 171.6 169.4 169.0 160.8 156.5 150.2 143.8 139.8 139.5 135.8 132.1 131.2 131.1 131.0 130.8 127.3 126.2 122.3 122.2 122.2 120.9 120.2 119.4 117.4 115.0 112.7 111.2 111.2 109.5 108.2
56.8 50.9 50.4 49.0 CD3OD 48.2 42.0 41.7 38.9 32.2 32.1 28.5 27.7 23.8 23.3 23.0
F
F
N
N
N
N
N
N
N
O
O
O
NH
O
O
NH
dCBP-29
190 180 170 160 150 140 130 120 110 100 90 80 70 60 50 40 30 20 10 0
f1 (ppm)

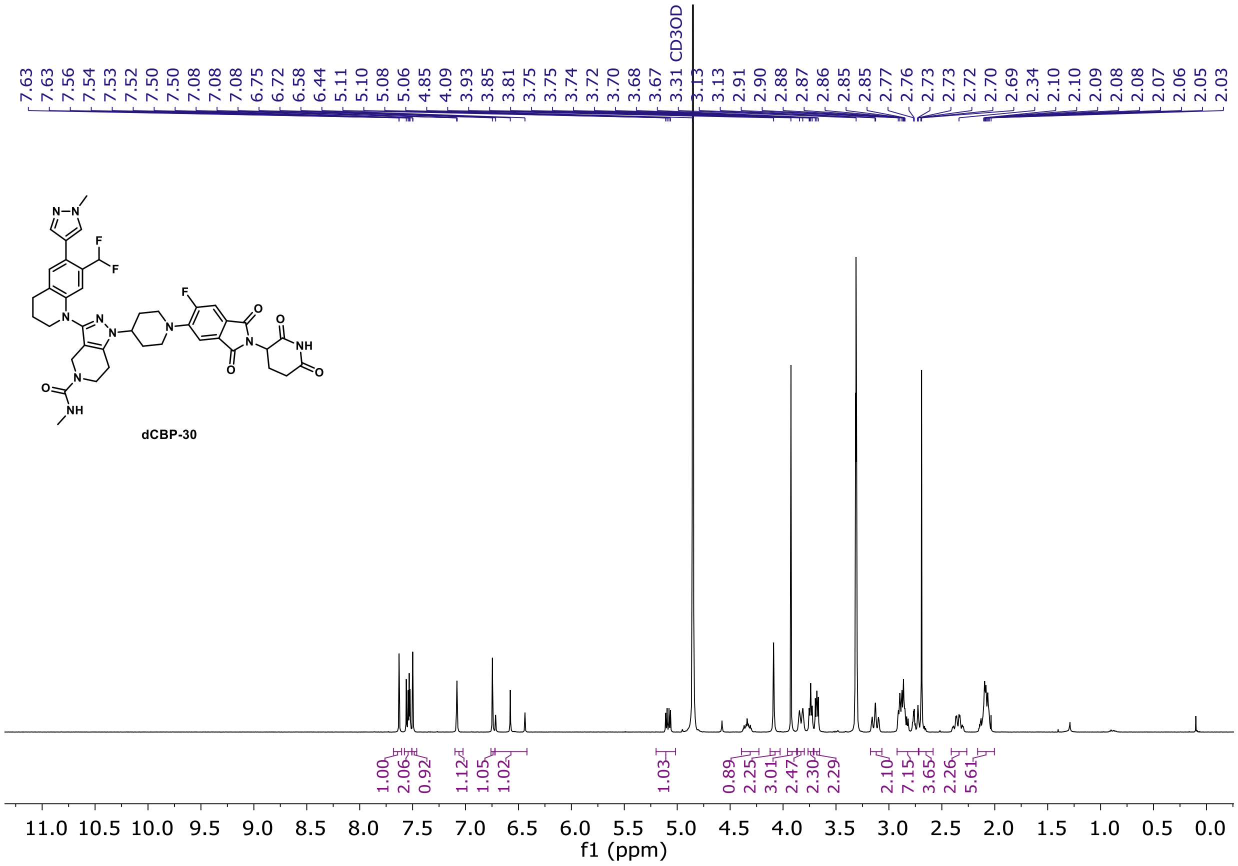
7.63 7.63 7.56 7.54 7.53 7.52 7.50 7.50 7.08 7.08 7.08 6.75 6.72 6.58 6.44 5.11 5.10 5.08 5.06 4.85 4.09 3.93 3.85 3.81 3.75 3.75 3.74 3.72 3.70 3.68 3.67 3.31 CD3OD 3.13 3.13 2.91 2.90 2.88 2.87 2.86 2.85 2.77 2.76 2.73 2.73 2.72 2.70 2.69 2.34 2.10 2.10 2.09 2.08 2.08 2.07 2.06 2.05 2.03
dCBP-30
1.00 2.06 0.92 1.12 1.05 1.02 1.03 0.89 2.25 3.01 2.47 2.30 2.29 2.10 7.15 3.65 2.26 5.61
11.0 10.5 10.0 9.5 9.0 8.5 8.0 7.5 7.0 6.5 6.0 5.5 5.0 4.5 4.0 3.5 3.0 2.5 2.0 1.5 1.0 0.5 0.0
f1 (ppm)

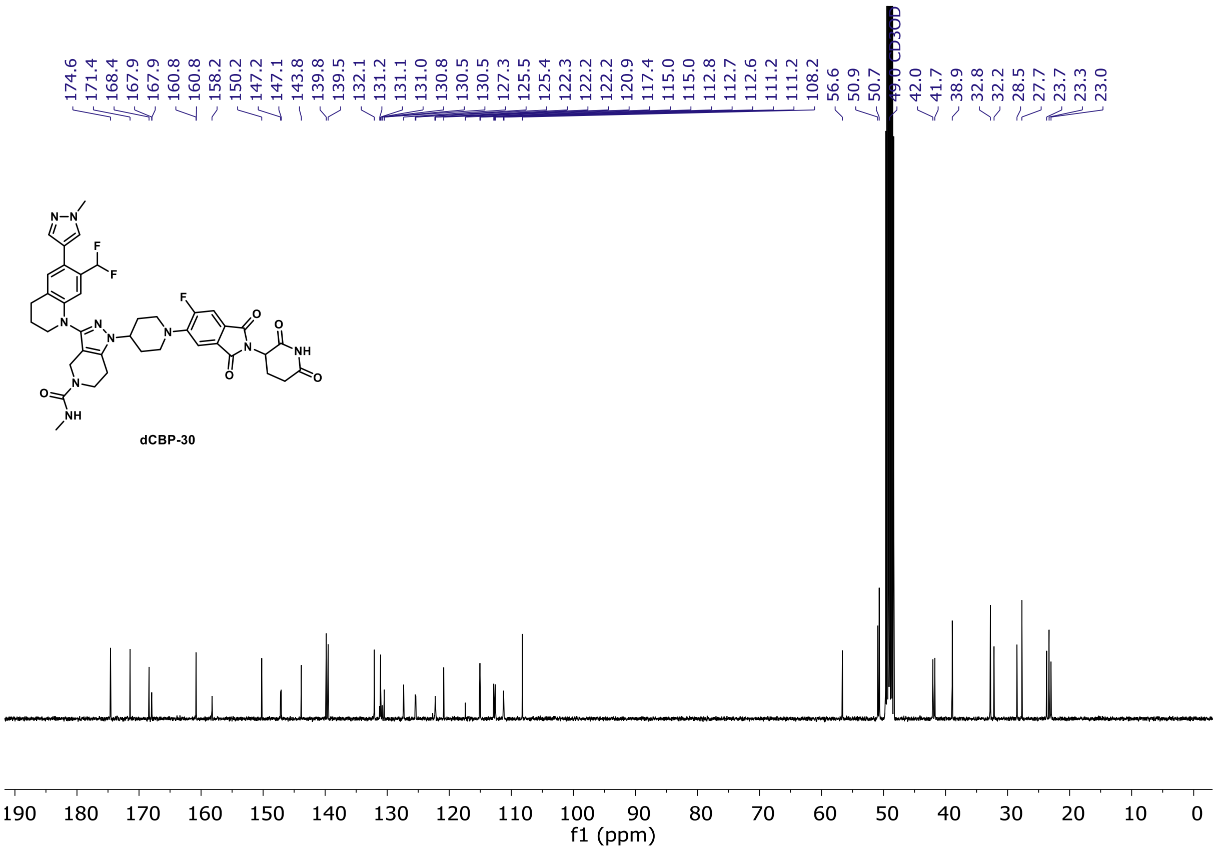
174.6 171.4 168.4 167.9 167.9 160.8 160.8 158.2 150.2 147.2 147.1 143.8 139.8 139.5 132.1 131.2 131.1 131.0 130.8 130.5 130.5 127.3 125.5 125.4 122.3 122.2 122.2 120.9 117.4 115.0 115.0 112.8 112.7 112.6 111.2 111.2 108.2 56.6 50.9 50.7 49.0 CD3OD 42.0 41.7 38.9 32.8 32.2 28.5 27.7 23.7 23.3 23.0
dCBP-30
190 180 170 160 150 140 130 120 110 100 90 80 70 60 50 40 30 20 10 0
f1 (ppm)

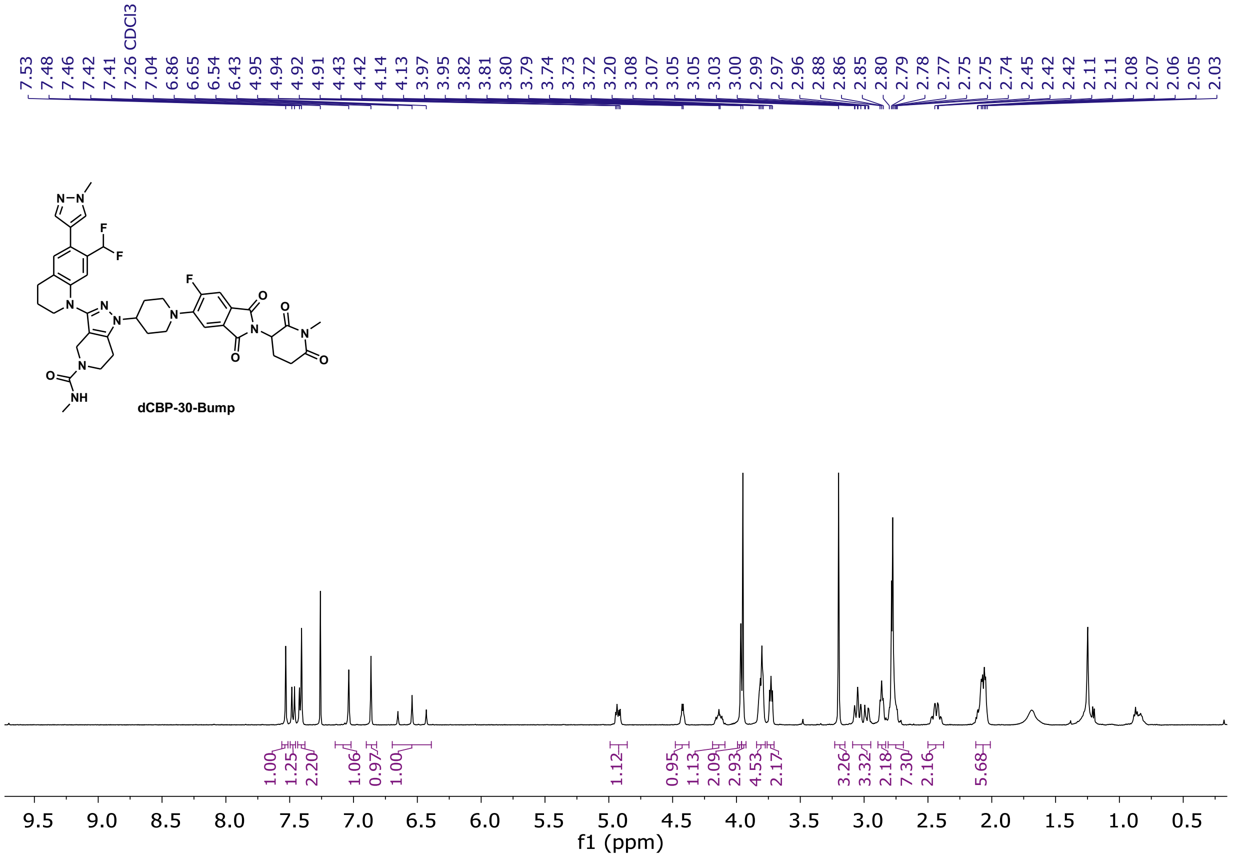
7.53 7.48 7.46 7.42 7.41 7.26 CDCl3 7.04 6.86 6.65 6.54 6.43 4.95 4.94 4.92 4.91 4.43 4.42 4.14 4.13 3.97 3.95 3.82 3.81 3.80 3.79 3.74 3.73 3.72 3.20 3.08 3.07 3.05 3.05 3.03 3.00 2.99 2.97 2.96 2.88 2.86 2.85 2.80 2.79 2.78 2.77 2.75 2.75 2.74 2.45 2.42 2.42 2.11 2.11 2.08 2.07 2.06 2.05 2.03
F
F
F
O
O
O
N
N
N
N
N
N
N
N
O
O
NH
dCBP-30-Bump
1.00 1.25 2.20 1.06 0.97 1.00 1.12 0.95 1.13 2.09 2.93 4.53 2.17 3.26 3.32 2.18 7.30 2.16 5.68
9.5 9.0 8.5 8.0 7.5 7.0 6.5 6.0 5.5 5.0 4.5 4.0 3.5 3.0 2.5 2.0 1.5 1.0 0.5
f1 (ppm)

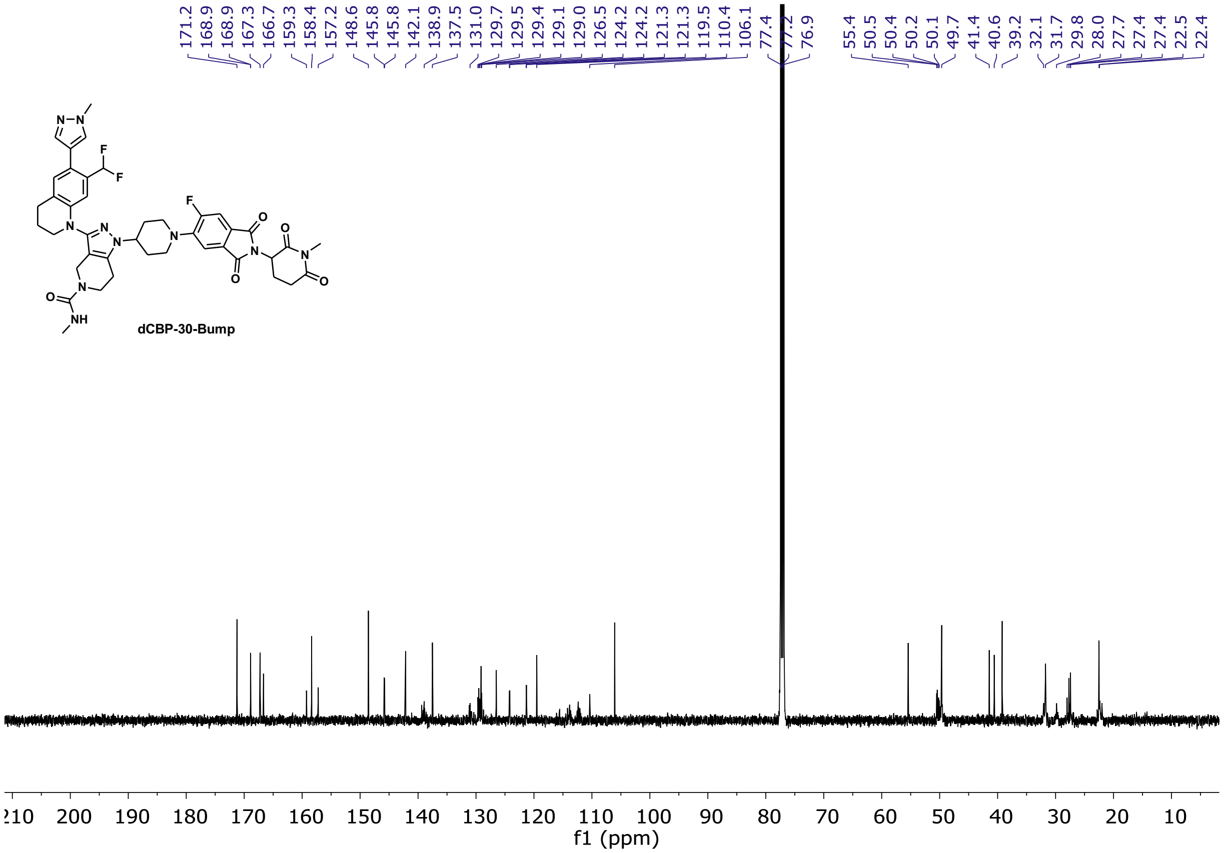
171.2 168.9 168.9 167.3 166.7 159.3 158.4 157.2 148.6 145.8 145.8 142.1 138.9 137.5 131.0 129.7 129.5 129.4 129.1 129.0 126.5 124.2 124.2 121.3 121.3 119.5 110.4 106.1 77.4 77.2 76.9 55.4 50.5 50.4 50.2 50.1 49.7 41.4 40.6 39.2 32.1 31.7 29.8 28.0 27.7 27.4 27.4 22.5 22.4
F
F
F
O
O
O
N
N
N
N
N
N
N
N
O
O
NH
dCBP-30-Bump
210 200 190 180 170 160 150 140 130 120 110 100 90 80 70 60 50 40 30 20 10
f1 (ppm)
