# Supplementary material for: An orally active dual CBP/p300 degrader targets core dependencies of multiple myeloma
Source: Cell Rep. Author manuscript; Available in PMC 2026 Jul 14. (PMC13366507; doi:10.1016/j.celrep.2026.117464)
Supplement: 2 [file NIHMS2190731-supplement-2.pdf]

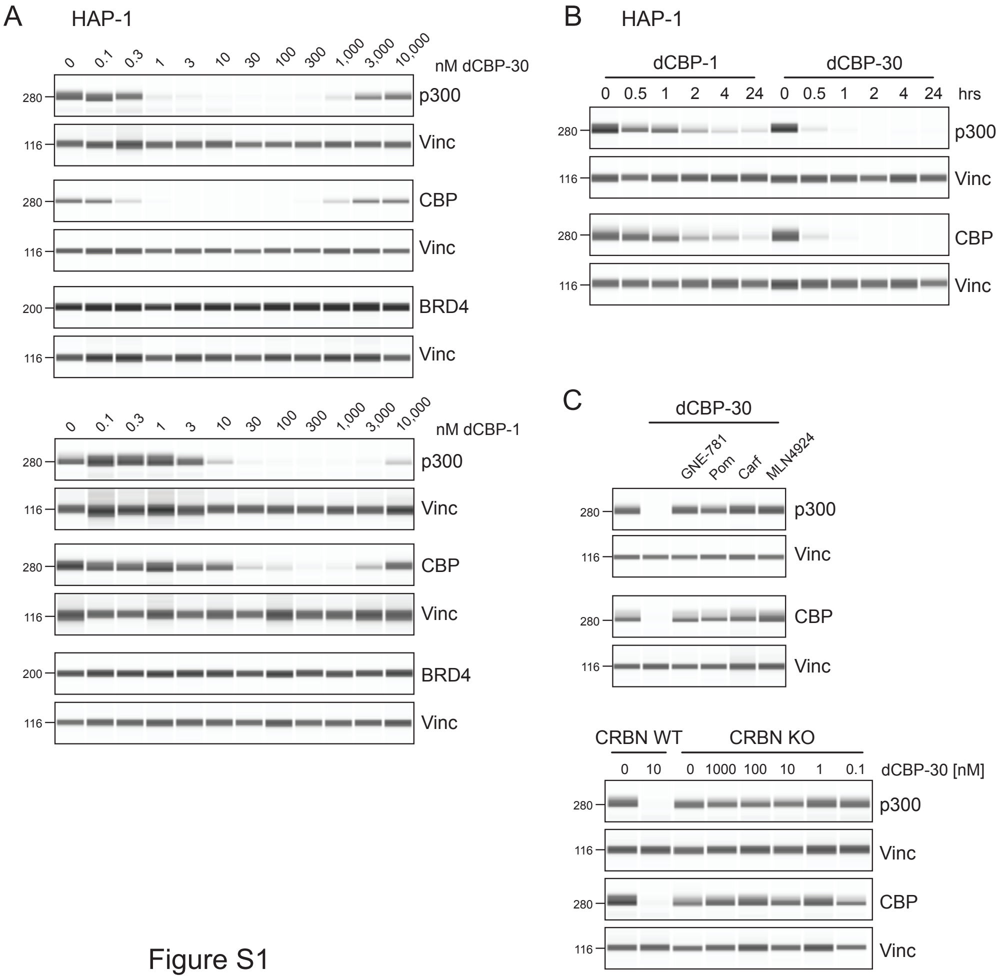

Figure S1

Figure S1. Endogenous CBP and p300 degradation in HAP1 cells, related to Figure 2.
(A) Immunoassay measurements of CBP, p300, and BRD4 levels with dCBP-30 (top) or dCBP-1 (bottom) dose-response treatments with 4 hours drug exposure in HAP1 cells; vinculin levels shown as loading controls. **(B)** Time course immunoassay measurements of CBP and p300 levels with dCBP-30 or dCBP-1 treatment over 24 hours in HAP1 cells, 10 nM. **(C)** CBP and p300 levels following 6 hours pre-treatment of GNE-781 (10 μM), pomalidomide (Pom, 10 μM), carfilzomib (Carf, 400 nM), MLN4924, (1 μM), followed by 2 hours treatment with dCBP-30 (10 nM) (top panel); CBP and p300 levels following 4 hours dCBP-30 treatment of wildtype and CRBN knockout HAP1 cells (bottom panel).

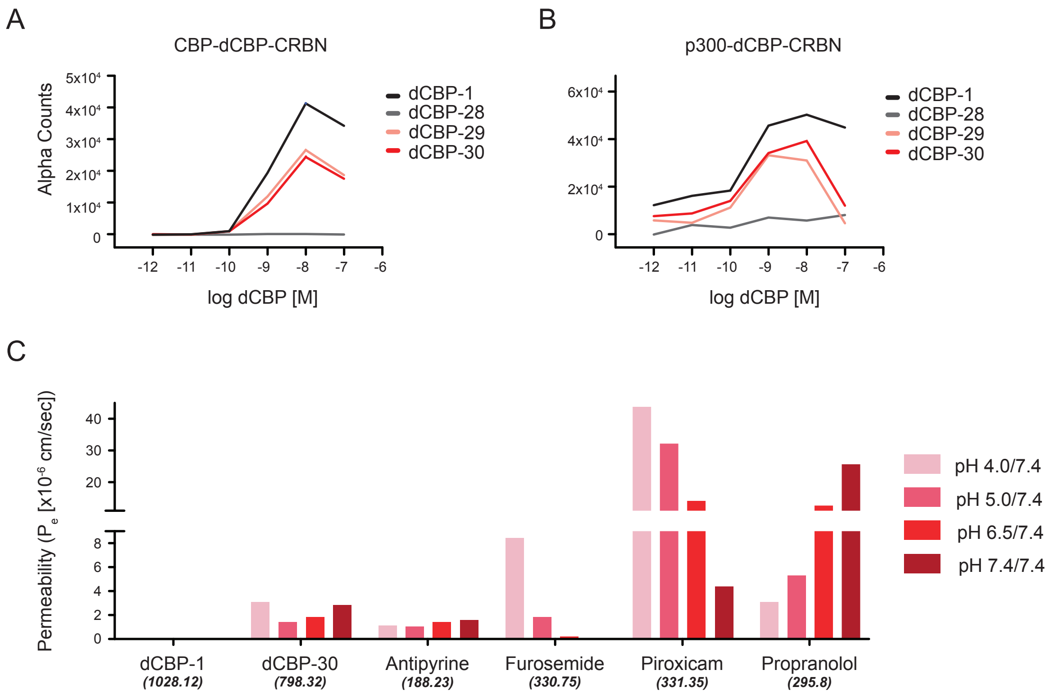

Figure S2

Figure S2. Ternary complex formation with dCBP-1, dCBP-28, dCBP-29, dCBP-30; PAMPA permeability analysis of dCBP-1 and dCBP-30, related to Figure 2.

(A) AlphaScreen ternary complex of CBP-dCBP-CRBN. **(B)** AlphaScreen ternary complex of CBP-p300-CRBN. Data represents the average of three replicate experiments; background signal was subtracted from each well. **(C)** PAMPA assay results comparing dCBP-1 and dCBP-30 at varying pH gradients. Antipyrine, furosemide, piroxicam, and propranolol are shown as controls. Molecular weight of each compound is listed below compound name.

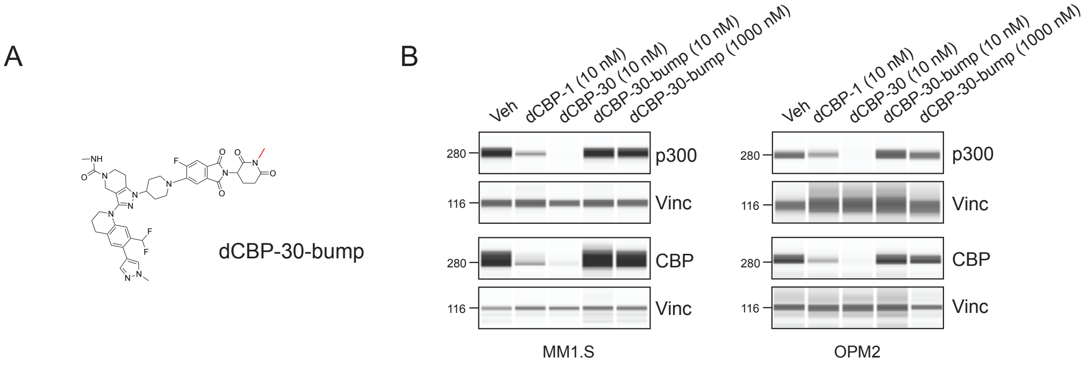

Figure S3

Figure S3. dCBP-30-bump is a control compound with no degradation activity, related to Figure 3.
(A) Structure of dCBP-30-bump. **(B)** Immunoassay measurements of p300 and CBP comparing dCBP-1, dCBP-30, and dCBP-30-bump, 4 hours treatment of MM1.S and OPM2 cells.

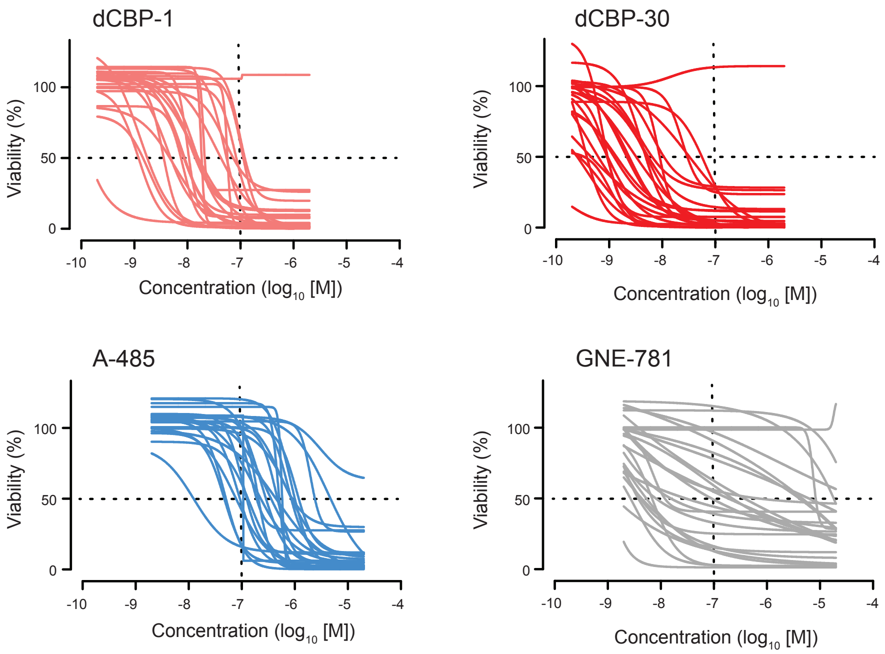

Figure S4

Figure S4. Dose response curves of MM cell lines treated with dCBP-1, dCBP-30, A-485, and GNE-781, related to Figure 4.
Curves fitted over 9-point dose response experiment using the average of two replicates for each cell line using GraphPad Prism (four parameter, variable slope). Twenty-five cell lines (listed in Figure 4A) were tested for each compound with five-day incubation.

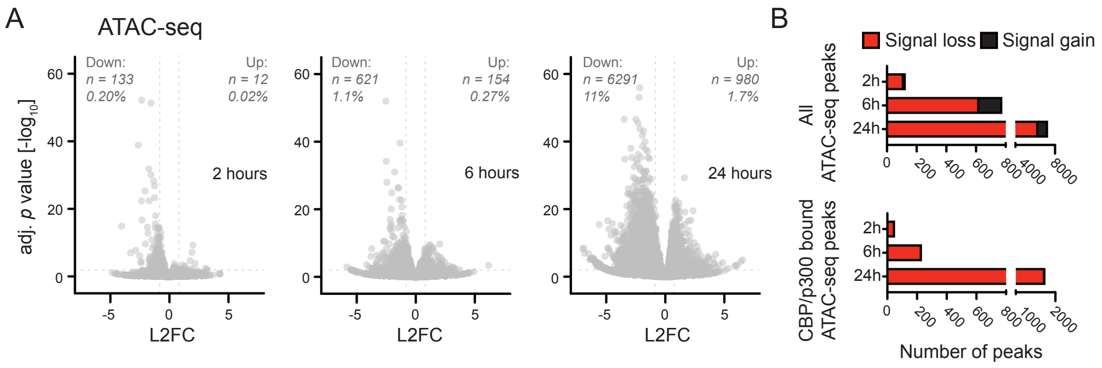

Figure S5

Figure S5. Quantification of chromatin accessibility changes in MM1.S cells following dCBP-30 treatment, related to Figure 5.

(A) Time-course volcano plots of chromatin accessibility changes as measured by ATAC-seq following dCBP-30 treatment (10 nM) in MM1.S cells. L2FC, Log_2 fold change. Cutoffs for gene quantification: *p* value > 0.1, L2FC > or < 1. **(B)** Quantification of all ATAC-seq peak changes (top) and at CBP/p300 bound regions (bottom).

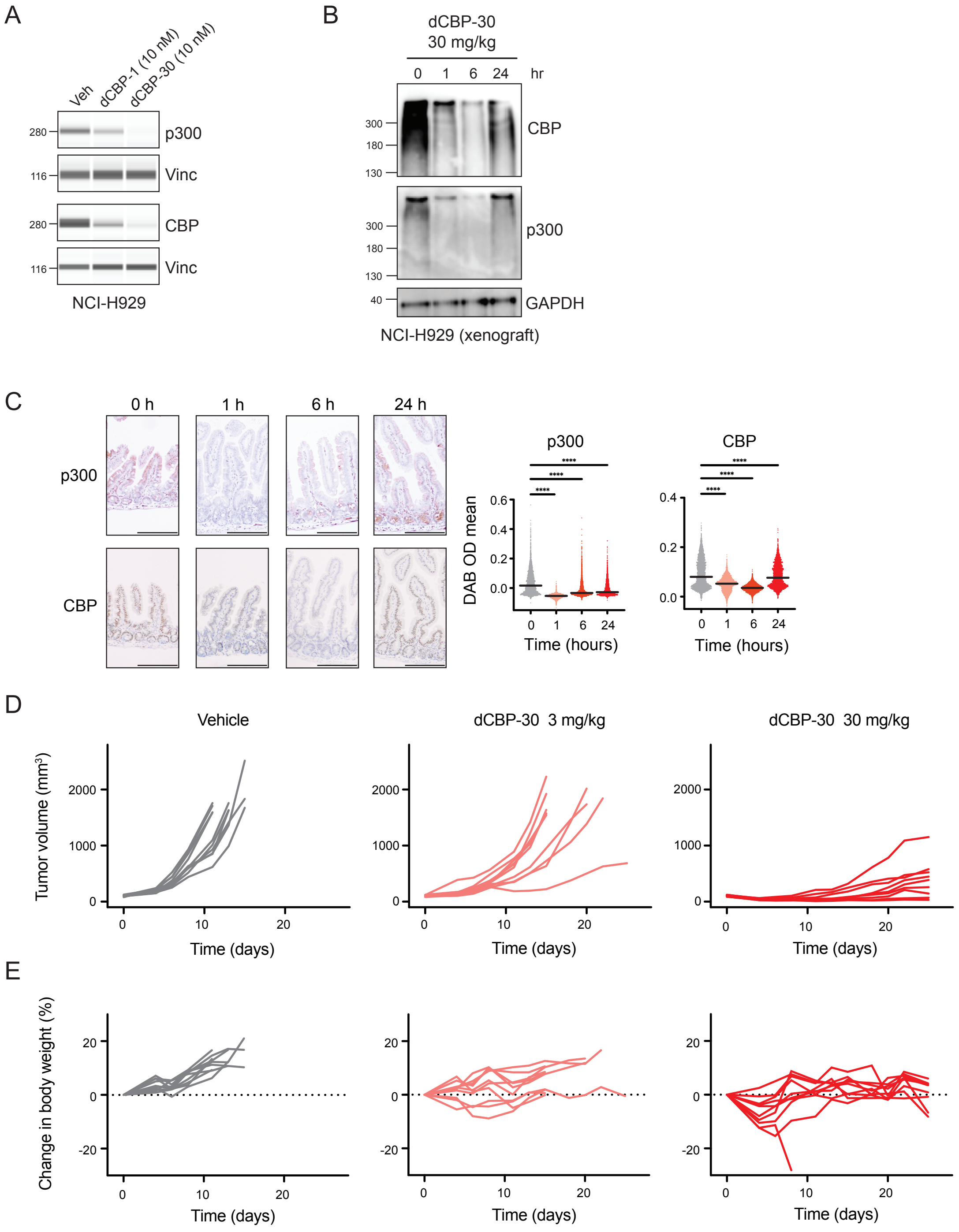
A
Veh
dCBP-1 (10 nM)
dCBP-30 (10 nM)
p300
Vinc
CBP
Vinc
NCI-H929
B
dCBP-30
30 mg/kg
0 1 6 24 hr
CBP
p300
GAPDH
NCI-H929 (xenograft)
C
0 h
1 h
6 h
24 h
p300
CBP
DAB OD mean
Time (hours)
D
Vehicle
dCBP-30 3 mg/kg
dCBP-30 30 mg/kg
Tumor volume (mm³)
Time (days)
E
Change in body weight (%)
Time (days)

Figure S6

Figure S6. Single agent *in vivo* activity of dCBP-30 in xenografted MM cells, related to Figure 6.
(A) Immunoassay measurements of CBP, p300, levels with dCBP-30 or dCBP-1 treatment of NCI-H929 cells with 4 hours drug exposure; vinculin (Vinc) levels shown as loading controls. **(B)** Immunoblot detection of CBP and p300 in formalin-fixed NCI-H929 xenografted tumors at time points indicated following a single dose of dCBP-30, 30 mg/kg. **(C)** Immunohistochemistry of CBP and p300 expression in mouse intestine at time points indicated following a single dose of dCBP-30 (30 mg/kg) in NSG mice harboring subcutaneously xenografted NCI-H929 cells (see Figure 6B for tumor staining). Representative images of tissue shown at left, quantification of staining intensity at right (n = 4000 randomly selected cells). ****, $p < 0.0001$, *ns* = not significant; scale bars = 50 μm; line on each plot represents median. **(D)** Tumor volume of individual NCI-H929 engrafted mice treated intermittently with 3 or 30 mg/kg dCBP-30, BID. **(E)** Individual mouse weights of dCBP-30 treated mice from (D).

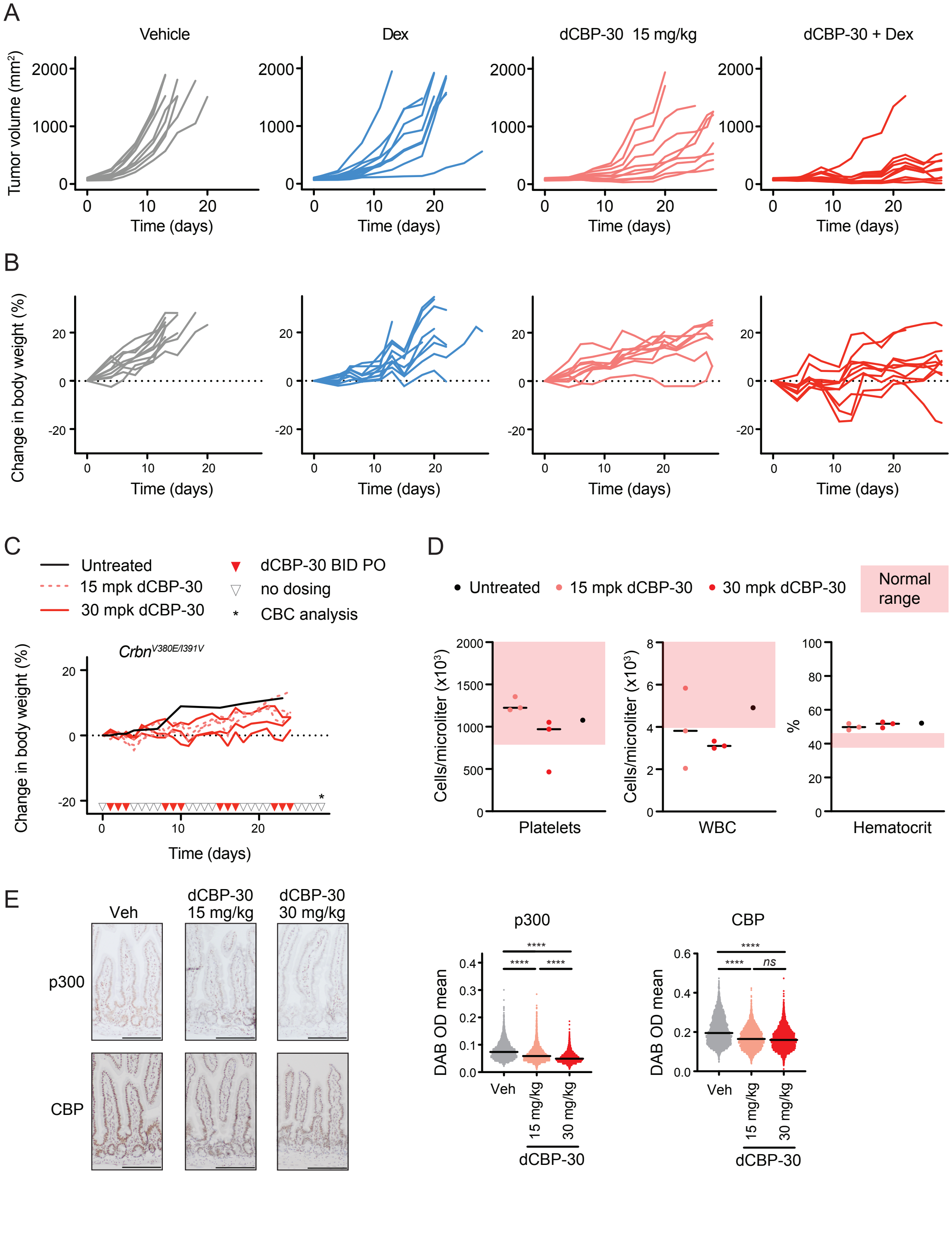
A
Vehicle
Dex
dCBP-30 15 mg/kg
dCBP-30 + Dex
Tumor volume (mm²)
Time (days)
B
Change in body weight (%)
Time (days)
C
Untreated
15 mpk dCBP-30
30 mpk dCBP-30
dCBP-30 BID PO
no dosing
CBC analysis
Crbn V380E/I391V
Change in body weight (%)
Time (days)
D
Untreated
15 mpk dCBP-30
30 mpk dCBP-30
Normal range
Cells/microliter (x10³)
Platelets
WBC
%
Hematocrit
E
Veh
dCBP-30 15 mg/kg
dCBP-30 30 mg/kg
p300
CBP
DAB OD mean
Veh
15 mg/kg
30 mg/kg
dCBP-30
****
ns

Figure S7

Figure S7. *In vivo* activity of dCBP-30 in combination with dexamethasone; effects of dCBP-30 in C57BL/6 *Crbn*$^{V380E/I391V}$ mice, related to Figure 7.

(A) Tumor volume of individual NCI-H929 engrafted mice treated intermittently with 15 mg/kg dCBP-30, BID and 1 mg/kg dexamethasone (Dex) twice weekly. **(B)** Individual weights of treated mice from (A). **(C)** Individual weights of C57BL/6 *Crbn*$^{V380E/I391V}$ mice treated with either 15 mg/kg or 30 mg/kg dCBP-30; one untreated mouse was compared with n = 3 of each dCBP-30 treated group. **(D)** Hematology analysis (platelets, white blood cells (WBC), hematocrit) of mice from (C) on day 28 (endpoint) of study comparing the dCBP-30 (15 mg/kg and 30 mg/kg, n = 3 for each group) with untreated control mouse (n = 1). Highlighted is the normal blood count range for C57BL/6 mice[51]; line on each plot represents median. **(E)** Immunohistochemistry of CBP and p300 expression in mouse intestine following a single dose of dCBP-30 (30 mg/kg or 15 mg/kg) in C57BL/6 *Crbn*$^{V380E/I391V}$ mice. Representative images of tissue shown at left, quantification of staining intensity at right (n = 4000 randomly selected cells). ****, $p < 0.0001$, *ns* = not significant; scale bars = 50 μm; line on each plot represents median.
